# Supplementary material for: The epidemiological impact of digital and manual contact tracing on the SARS-CoV-2 epidemic in the Netherlands: Empirical evidence
Source: PLOS Digit Health. 2023 Dec 29;2(12):e0000396. doi: 10.1371/journal.pdig.0000396 (PMC10756539; doi:10.1371/journal.pdig.0000396)
Supplement: S1 Text — (DOCX) [file pdig.0000396.s001.docx]

**The epidemiological impact of digital and manual contact tracing on the SARS-CoV-2 epidemic in the Netherlands: empirical evidence.**

**Authors:** Wianne Ter Haar, Jizzo Bosdriesz, Roderick P. Venekamp, Ewoud Schuit, Susan van den Hof, Wolfgang Ebbers, Mirjam Kretzschmar, Jan Kluijtmans, Carl Moons, Maarten Schim van der Loeff, Amy Matser, Janneke H. H. M. van de Wijgert

**Supplementary INTRODUCTION**

*Milestones in the Dutch SARS-CoV-2 epidemic during the data collection period*

The data collection period was from 1 June 2020 to 14 June 2021 and some important milestones related to SARS-CoV-2 governmental measures, testing policies, and manual contact tracing (MCT) phases took place in the Netherlands during this time period.^1^ The original Wuhan virus variant circulated from February to December 2020: the first confirmed case was announced on 27 February and the first death on 6 March.^2^ Travel and contact reduction measures were gradually introduced between 9 March and 28 April 2020 and gradually relaxed thereafter, with the peak of this first wave occurring on 8 April 2020. Public testing was introduced on 1 June 2020 (see below). A second wave occurred between late September 2020 and late January 2021. During this wave, the Wuhan virus variant was gradually replaced by the Alpha variant, which remained in circulation until mid-August 2021. A partial lockdown came into effect on 14 October 2020 and a strict lockdown (with a maximum of two visitors allowed per day, all non-essential shops closed, and all education online) on 15 December 2020. The Dutch COVID-19 vaccination campaign started on 6 January 2021. On 23 January 2021, a curfew was imposed from 9 pm until 4:30 am. Between 1 March and 5 June 2021, restrictions were gradually lifted as vaccination levels increased. After the data collection period, short epidemic waves due to the Delta variant occurred in July 2021 and October/November 2021, and a massive wave due to Delta and Omicron variants from December 2021 until April 2022.

SARS-CoV-2 testing at Public Health Service (PHS) sites became available for anyone with symptoms from 1 June 2020 onwards.^1,3,4^ Close contacts without symptoms were advised to get tested on the fifth day after their last contact with an index case between 1 December 2020 and 18 February 2021, and to get tested as soon as possible after exposure notification as well as on the fifth day since last contact from 18 February 2021 onwards. Commercial test sites catering to asymptomatic individuals that needed proof of a negative test in order to gain access to events or for travel were available from February 2021 until 23 March 2022. On 31 March 2021, the first SARS-CoV-2 rapid antigen self-tests became available in pharmacies and stores.^5^ Until February 2022, individuals with a positive self-test were asked to do a repeat test at a PHS test site. Testing at PHS sites was almost exclusively by PCR test throughout the epidemic. Commercial testing could be done by either PCR or antigen lateral flow test, depending on the access or travel requirements of the person requesting a test. Self-testing was exclusively by antigen lateral flow test. Results of PCR tests initially took a few days to become available but were available within two days later on in the epidemic. The sensitivity and specificity of these tests was very high for all virus variants. Later flow tests produced a result immediately but were less sensitive and specific, especially in asymptomatic individuals.^6,7^

Implementation of the MCT programme was phased regionally depending on regional infection pressure and MCT capacity.^4^ These phases were numbered one to five. Phase 1 consisted of professional notification of all contacts reported by each index case and complete surveillance data collection. We considered MCT phases 3-5 ‘scaled-down’ because in these phases, index cases were asked to notify their contacts themselves and only limited surveillance data was collected. In the period covering the PHS Amsterdam dataset (1 December 2020 - 31 May 2021), the MCT programme in Amsterdam was scaled down between 7 December 2020 and 13 January 2021 and again between 15 March and 19 May 2021. During the first RDT study (14 December 2020 - 6 February 2021), the MCT programme in Rotterdam was scaled down until 30 December 2020 and in West-Brabant until 6 January 2021. During the second RDT study (12 April - 18 June 2021), the MCT programmes in West-Brabant and Rotterdam (but not Zwolle) were scaled down between 12 April and 19 May 2021.

## *CoronaMelder smartphone application technical details*

The Dutch government passed the Temporary Act Notification Application COVID-19 (Tijdelijke Wet Notificatieapplicatie Covid-19 in Dutch) on 6 October 2020, and launched the national contact tracing app dubbed CoronaMelder on 10 October 2020.^8^ The app used the decentralized protocol of the Google/Apple Exposure Notification (GAEN) Application Programming Interface (API).^9^ GAEN was inspired by the Decentralized Privacy-Preserving Proximity Tracing (DP-3T) protocol, which was developed by an international group of technologists, engineers, epidemiologists, and legal experts.^10,11^ CoronaMelder was developed by a team commissioned by the Dutch Ministry of Health, Welfare and Sports in the summer of 2020.

The app generated a 16-byte random string called Temporary Exposure Key (TEK) approximately every 24 hours. TEKs were transformed into Rolling Proximity Identifiers (RPIs) using the hash function and the AES algorithm, and these RPIs were broadcast through Bluetooth Low Energy by the user’s smartphone. The RPIs changed every time the Bluetooth Low Energy randomised address changed (every 15 minutes on average). In this way, both TEKs and RPIs could not be traced back to individuals.^10^ RPIs of other CoronaMelder users, together with Received Signal Strength Indicators (a proxy for the distance between two smartphones) and the cumulative contact duration, were stored locally on the user’s phone for 14 days. Additionally, the user’s own TEKs were stored on the phone for 14 days. This duration of 14 days was based on the assumption that SARS-CoV-2 could potentially be transmitted over a period of 14 days after exposure. When smartphones with CoronaMelder in active mode came into contact, RPIs together with the indicators of distance and duration of contact were exchanged.

When MCT capacity was optimal (phase 1), PHS staff phoned all individuals who had tested positive at a test site and asked them if they were using CoronaMelder. If yes, they asked the user if they would like to share their unique PHS-key as shown in the app (Figure S1). If yes, the PHS employee added the date of symptoms onset (or of the positive test, if no symptoms were present) to this key. The app user subsequently received a notification (a blue pop-up button), which s/he could (voluntarily) press to upload their TEKs to the backend server. From 11 November 2021 onwards (which is after the three datasets used in this study had already been collected), the app user could upload their PHS-key themselves rather than having to contact the PHS service first.^12^ The backend server was managed by KPN, a Dutch telecommunications company, via CIBG, which is an implementing organization of the Dutch Ministry of Health, Welfare and Sports. After uploading, the TEKs were converted to Diagnostic Keys (DKs) and stored on the backend server for 14 days. Each time an app user’s smartphone connected to the server (approximately six times per day), the app downloaded the list of current DKs and their corresponding RPIs and compared them to the RPIs that were saved on the phone. If there was a match, the proximity, contact duration, and infectious window (based on symptom onset or positive test date) of the infected close contact were used to determine whether transmission may have occurred. If this was the case, the app user received an exposure notification (Figure S1), showing the date of this potential transmission and advice on what to do next. Once the app user had read the notification, it was automatically removed from their phone permanently.

*The exposure notification cascade*

For DCT (and MCT) to have epidemiological impact, all steps of the exposure notification cascade should be optimised. Figure 1 in the main manuscript visualises this cascade. It consists of two separate yet interconnected cascades: part A and part B. An individual enters the cascade after downloading the app (A1). Some individuals may drop out of the cascade after this step because they uninstall the app or have their Bluetooth Low Energy (always or intermittently) turned off. In A2, CoronaMelder is active and exchanging RPIs with other app users through Bluetooth Low Energy. App users spent most of their time in A1 and A2 with the app running in the background. At some point, an exposure notification could have been triggered by CoronaMelder (A3), but also by the MCT programme (B2), by inner circle notification (hearing via the inner circle that an exposure might have happened, either at the request of PHS staff when MCT was scaled down (B3) or spontaneously (A11)), or by self-referral (e.g. having COVID-19-like symptoms; A12). After a trigger, the general guidance throughout the epidemic (also included in CoronaMelder notifications) was to immediately go into quarantine (A5-6), to get tested (A7-9), and/or to request medical help if needed (A10). Exposed individuals were advised to stay in quarantine until receiving a negative test result or for a minimum of 10 days after the exposure (A6). They were advised to get tested as soon as possible after symptom onset; testing without symptoms became possible after 1 December 2020 (A7; see also above).

Individuals testing SARS-CoV-2-positive entered part B of the cascade. The app user who tested positive contacted, or was contacted by, a PHS employee (B1), who either conducted MCT (B2-3; depending on the MCT capacity at the time) and/or facilitated DCT (B3-4; this remained possible even if the MCT was scaled down). When the MCT programme was functioning optimally (phase 1), each person who tested positive was phoned by a PHS employee (B1) and this PHS employee also phoned the index cases’ identifiable close contacts (B2). However, when the MCT programme was scaled down, index cases were asked to warn their identifiable close contacts themselves (B3). Unidentifiable close contacts could only be warned via the DCT (B5) as described above.

**References**

1 National Institute of Public Health and the Environment. Tijdlijn van coronamaatregelen. 2022; published online March 16. https://www.rivm.nl/gedragsonderzoek/tijdlijn-maatregelen-covid (accessed Feb 5, 2023).

2 National Institute of Public Health and the Environment. Variants of the coronavirus SARS-CoV-2. 2022; published online March 16. https://www.rivm.nl/en/coronavirus-covid-19/virus/variants (accessed Feb 5, 2023).

3 National Institute of Public Health and the Environment. LCI-richtlijn COVID-19. Rijksinst. Voor Volksgezond. En Milieu. 2022; published online March 16. https://lci.rivm.nl/richtlijnen/covid-19 (accessed Feb 5, 2023).

4 Rijksoverheid.nl. GGD GHOR roadmap testen en traceren. https://www.rijksoverheid.nl/documenten/brieven/2020/10/14/roadmap-testen-en-traceren (accessed Feb 6, 2023).

5 Rijksoverheid.nl. Eerste zelftesten beschikbaar bij apotheken. 2021; published online March 31. https://www.rijksoverheid.nl/actueel/nieuws/2021/03/31/eerste-zelftesten-beschikbaar-bij-apotheken (accessed Feb 5, 2023).

6 Venekamp RP, Veldhuijzen IK, Moons KGM, *et al.* Detection of SARS-CoV-2 infection in the general population by three prevailing rapid antigen tests: cross-sectional diagnostic accuracy study. *BMC Med* 2022; 20: 97.

7 Schuit E, Veldhuijzen IK, Venekamp RP, *et al.* Diagnostic accuracy of rapid antigen tests in asymptomatic and presymptomatic close contacts of individuals with confirmed SARS-CoV-2 infection: cross sectional study. *BMJ* 2021; 374: n1676.

8 Ebbers W, Hooft L, van der Laan LN, Metting E. Evaluatie CoronaMelder: een overzicht na 9 maanden. 2021; published online May 28. https://www.rijksoverheid.nl/documenten/publicaties/2021/05/28/rapporten-evaluatie-coronamelder-9-maanden (accessed Feb 5, 2023).

9 Ministry of General Affairs. Cryptographic framework and back-end security evaluation Dutch COVID-19 notification app. 2020; published online Aug 28. https://www.rijksoverheid.nl/documenten/rapporten/2020/08/28/cryptographic-framework-and-back-end-security-evaluation (accessed Feb 5, 2023).

10 De Winter B, Lute E, Dasselaar A, Frenken-Farag M. Duidingsrapportage CoronaMelder: Informatiebeveiliging en privacybescherming. 2020; published online Aug 28. https://www.rijksoverheid.nl/documenten/rapporten/2020/08/28/duidingsrapportage-coronamelder-informatiebeveiliging-en-privacybescherming-stand-van-zaken-lanceringsadvies (accessed Feb 5, 2023).

11 Martin T, Karopoulos G, Hernández-Ramos JL, Kambourakis G, Nai Fovino I. Demystifying COVID-19 digital contact tracing: A survey on frameworks and mobile apps. *Wirel Commun Mob Comput* 2020; 2020: e8851429.

12 Rijksoverheid.nl. Op Coronatest.nl meteen besmetting doorgeven via CoronaMelder. 2021; published online Oct 14. DOI:10/14/op-coronatest.nl-meteen-besmetting-doorgeven-via-coronamelder.
