# Supplementary material for: The epidemiological impact of digital and manual contact tracing on the SARS-CoV-2 epidemic in the Netherlands: Empirical evidence
Source: PLOS Digit Health. 2023 Dec 29;2(12):e0000396. doi: 10.1371/journal.pdig.0000396 (PMC10756539; doi:10.1371/journal.pdig.0000396)
Supplement: S2 Text — (DOCX) [file pdig.0000396.s002.docx]

**The epidemiological impact of digital and manual contact tracing on the SARS-CoV-2 epidemic in the Netherlands: empirical evidence.**

**Authors:** Wianne Ter Haar, Jizzo Bosdriesz, Roderick P. Venekamp, Ewoud Schuit, Susan van den Hof, Wolfgang Ebbers, Mirjam Kretzschmar, Jan Kluijtmans, Carl Moons, Maarten Schim van der Loeff, Amy Matser, Janneke H. H. M. van de Wijgert

**SUPPLEMENTARY METHODS**

*Description of data sources*

We could not use data collected via the CoronaMelder application itself due to the privacy sensitive configuration of the app. Instead, we used routinely collected public health data at the PHS Amsterdam and data from two SARS-CoV-2 rapid diagnostic test (RDT) accuracy studies that were conducted at various other PHS test sites in the Netherlands.^1,2^

PHS Amsterdam data was extracted from the national CoronIT, HPZone, and Osiris databases. CoronIT is a national database established by the Dutch Ministry of Health, Welfare, and Sports specifically for use during the SARS-CoV-2 pandemic. The CoronIT database includes test request data such as age, gender, postal code, reason for testing, having symptoms (and if yes, which ones) at the time of test request, the date/time of test appointment, the date/time of testing, and the test result. HPZone is the MCT database that is used by PHS MCT programmes throughout the country. The PHS Amsterdam HPZone data that we had access to for this study included whether an individual was a case or a contact (the status of contacts who subsequently tested positive changed from contact to case), exposure date, test result, and sociodemographic information. Osiris is the national surveillance database of the National Institute of Public Health and the Environment where all notifiable infections (including SARS-CoV-2) are reported. The only Osiris variable that we used was the level of exposure of those contacted via the MCT program (household contact, close contact of long duration, close contact of short duration, and other contact). At the PHS Amsterdam, for each individual who tested positive, HPZone and Osiris data were entered at the same time by the same employee.

The PHS Amsterdam dataset containing only CoronIT data between 1 December 2020 and 31 May 2021 included 562,159 tests (nt) by 372,545 individuals (ni) (Figure S2). For the exposure-testing interval analyses, CoronIT data were merged with HPZone and Osiris data because exposure dates were only available in HPZone and levels of exposure only in Osiris. This subset of the PHS Amsterdam dataset (referred to as the PHS MCT subset because all individuals in this dataset had been part of the MCT programme as either a case or a contact or both) included 20,647 exposure-testing intervals (n_e-t_) by 20,355 individuals (n_i_) (Figure S2). The merging process was as follows. HPZone data was selected based on whether an exposure date (ne) was present. A subset was made for the period in which the exposure took place two weeks before 1 December 2020 (17 November 2020) and before 31 March 2021 due to data availability. Duplicate entries were removed. Next, the Osiris variable for level of exposure was added using personal identifiers for merging with HPZone. Duplicate entries based on personal identifiers and degree of contact were removed. The CoronIT dataset between 1 December 2020 and 31 March 2021 was used to add the test date (t), again using personal identifiers for merging. Some individuals had multiple exposure- testing intervals (ne-t). For example, one individual was registered as a contact in HPZone at two different time points and got tested a total of 6 times across the duration of the study period. For this person, 12 exposure-testing intervals were present in the combined dataset. After merging, the combined dataset was cleaned. Entries in which the exposure date was after the testing date were removed. When multiple test-dates and one exposure date were present, the most relevant test-date was selected and the others removed. For example, when two test dates were far apart, only the test date closest to the exposure date was kept. When multiple tests were done soon after one exposure, only the first test-date after the exposure was kept. Furthermore, when exposure-testing intervals were more than 14 days, the test-date was set to missing. We assumed that in these cases, another reason than the exposure recorded in the database likely triggered the testing. Finally, entries with an unknown exposure-testing interval were removed from the dataset because the reason for testing was not available for these cases.

The RDT studies were previously published and the logistics summarised in the main manuscript.^1,2^ We used data from the RDT study-specific questionnaires (Figure S3). The first RDT study dataset contained 4,126 individuals (Figure S4). The second RDT study dataset contained 7,925 individuals but date of last exposure, and thereby an exposure-testing interval, was only available for 3,172 individuals (Figure S4). The participants had already completed the routine PHS questionnaire when booking a test online, or answered questions by PHS staff over the phone, but that routine data was not available to us for this study. The data from the two or three regions participating in each RDT study, respectively, were combined to form one dataset per study. In the first RDT study, the reason for testing question had four answer options (tick all that apply): notified via CoronaMelder, MCT, or index case, or testing at one’s own initiative because someone in the social circle tested positive (coded as ‘self’); the study included only individuals who were asymptomatic at test request and therefore did not ask about symptoms as a reason for testing. In the second RDT study, the reason for testing question had five main answer options (tick all that apply): having symptoms, having been in contact with someone who tested positive, the individual’s general practitioner (GP) advised testing, having travelled to an orange/red country, or other. Individuals who ticked the second option were additionally asked for a last date of contact with the index case, and for the following types of notification/contacts: CoronaMelder, MCT, index case, housemate, or testing at one’s own initiative (‘self’). The ‘self’ category in the first RDT study likely includes both the housemate and ‘self’ categories of the second RDT study.

*Supplementary statistical analyses*

In all datasets, individuals could report multiple reasons for testing, and we used a categorisation hierarchy to limit the number of reasons for testing categories. The hierarchy in the PHS Amsterdam dataset was DCT notification, MCT notification, having symptoms without notification, or unknown: all individuals who reported having received a CoronaMelder notification as a reason for testing were included in the DCT group, individuals who reported having been contacted by the MCT but did not report a CoronaMelder notification were included in the MCT group, and individuals who reported having symptoms as a reason for testing and not DCT or MCT were included in the symptoms group. The hierarchy in the first RDT study was DCT notification, MCT notification, having been notified by an index case, testing at one’s own initiative (self), and unknown; symptoms were never a reason for testing because only asymptomatic individuals were eligible. The hierarchy in the second RDT study was DCT notification, MCT notification, having been notified by an index case, having a housemate who tested positive, testing at one’s own initiative (self), having received an unknown type of notification, having symptoms without notification, another reason for testing, and an unknown reason for testing. We used the last date of exposure/contact, and the date a test sample was taken, to calculate the exposure-testing interval in all datasets.

Log-likelihood test statistics showed that each multivariable Weibull model for each dataset was a statistically significantly better fit than the null-model (p <.001). The event time ratio (ETR), which signifies the relative difference in time intervals, the 95% confidence interval (CI), and the p-value were reported.

**References**

1 Venekamp RP, Veldhuijzen IK, Moons KGM, *et al.* Detection of SARS-CoV-2 infection in the general population by three prevailing rapid antigen tests: cross-sectional diagnostic accuracy study. *BMC Med* 2022; 20: 97.

2 Schuit E, Veldhuijzen IK, Venekamp RP, *et al.* Diagnostic accuracy of rapid antigen tests in asymptomatic and presymptomatic close contacts of individuals with confirmed SARS-CoV-2 infection: cross sectional study. *BMJ* 2021; 374: n1676.
