# Supplementary material for: The epidemiological impact of digital and manual contact tracing on the SARS-CoV-2 epidemic in the Netherlands: Empirical evidence
Source: PLOS Digit Health. 2023 Dec 29;2(12):e0000396. doi: 10.1371/journal.pdig.0000396 (PMC10756539; doi:10.1371/journal.pdig.0000396)
Supplement: S3 Text — (DOCX) [file pdig.0000396.s003.docx]

**The epidemiological impact of digital and manual contact tracing on the SARS-CoV-2 epidemic in the Netherlands: empirical evidence.**

**Authors:** Wianne Ter Haar, Jizzo Bosdriesz, Roderick P. Venekamp, Ewoud Schuit, Susan van den Hof, Wolfgang Ebbers, Mirjam Kretzschmar, Jan Kluijtmans, Carl Moons, Maarten Schim van der Loeff, Amy Matser, Janneke H. H. M. van de Wijgert

**SUPPLEMENTARY RESULTS**

In all three datasets, age and gender distributions differed statistically significantly across the reasons for testing groups, but these differences were small and considered not meaningful (Tables S1-3). Among individuals testing because of a DCT or MCT notification in the first RDT study, the proportions testing in West Brabant were statistically significantly higher than the proportions in the overall group; this was the other way around for Rotterdam (Table S2). In both regions, the MCT program was scaled down for approximately the first month of the two-month data collection period. Among individuals testing because of a MCT notification in the second RDT study, the proportion testing in Zwolle was statistically significantly higher, and in West-Brabant lower, than the proportions in the overall group (Table S3). During this study period, the MCT program was only scaled down in West-Brabant and Rotterdam, and not in Zwolle.

Among people who had a positive SARS-CoV-2 PCR test, the median PCR cycle threshold (Ct) value as a proxy of SARS-CoV-2 viral load ranged from 23.4 to 27.2 in the first RDT study (Table S2), and from 20.9 to 27.1 in the second RDT study, across reasons for testing (Table S3). In both studies, the median Ct values were lowest in the DCT group and highest in the unknown reasons for testing groups, but these differences did not reach significance in the first RDT study. The second RDT dataset additionally included data on COVID-19 vaccination status and having had a previous SARS-CoV-2 infection. The proportions of both variables were differentially distributed across reasons for testing (reaching significance for the former only), but the numbers were small (Table S3).

In the Weibull regression models of the PHS Amsterdam MCT subset, being aged 15-59 years was associated with a statistically significantly shorter exposure-testing interval than being in the youngest or oldest age groups, and male gender with a marginally longer interval (Table S4). In the first RDT study, those aged 60 or older had a statistically significantly longer mean interval than those in the 16-29 years group, but age was not associated with interval in the second RDT study (Tables S5-6). In both RDT studies, gender was not associated with interval in any of the time-to-event models.

The tobit regression models showed similar results as the Weibull models for the PHS Amsterdam MCT subset and the second RDT study (Tables S7 and S9). For the first RDT study, the results of the Weibull and tobit models did show some differences (Table S8). The Weibull model was considered a better fit because the data were not censored, but the data in this dataset were more skewed (testing peaked on day five after exposure due to the testing policy for asymptomatic close contacts at that time) than the data in the other two datasets.
