# Supplementary material for: The epidemiological impact of digital and manual contact tracing on the SARS-CoV-2 epidemic in the Netherlands: Empirical evidence
Source: PLOS Digit Health. 2023 Dec 29;2(12):e0000396. doi: 10.1371/journal.pdig.0000396 (PMC10756539; doi:10.1371/journal.pdig.0000396)
Supplement: S1 Fig — (DOCX) [file pdig.0000396.s004.docx]

**Figure S1: CoronaMelder smartphone application**

| 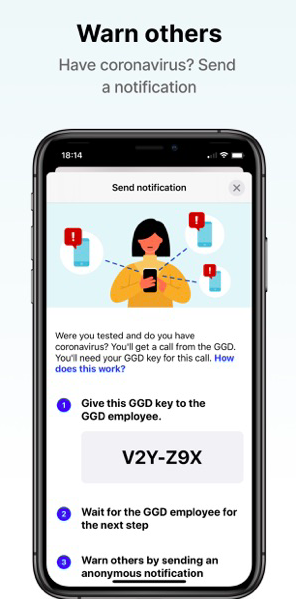 | 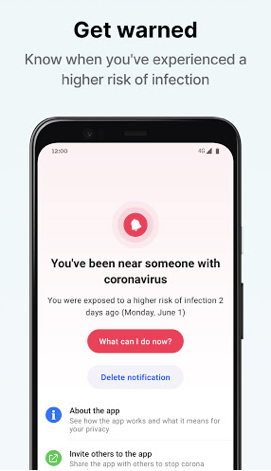 |
| --- | --- |
| Unique Public Health Service Key that would appear in the CoronaMelder app after the user tested SARS-CoV-2 positive | CoronaMelder exposure notification |

**Source:** De Winter et al, 2020. *Duidingsrapportage CoronaMelder: Informatiebeveiliging en privacybescherming* (report in Dutch language). Available at: https://www.rijksoverheid.nl/documenten/rapporten/ 2020/08/28/duidingsrapportage-coronamelder-informatiebeveiliging-en-privacybescherming-stand-van-zaken-lanceringsadvies (accessed Feb 5, 2023).
