# Supplementary material for: The epidemiological impact of digital and manual contact tracing on the SARS-CoV-2 epidemic in the Netherlands: Empirical evidence
Source: PLOS Digit Health. 2023 Dec 29;2(12):e0000396. doi: 10.1371/journal.pdig.0000396 (PMC10756539; doi:10.1371/journal.pdig.0000396)
Supplement: S2 Fig — (DOCX) [file pdig.0000396.s005.docx]

## Figure S2: Participant flow diagram PHS Amsterdam datasets


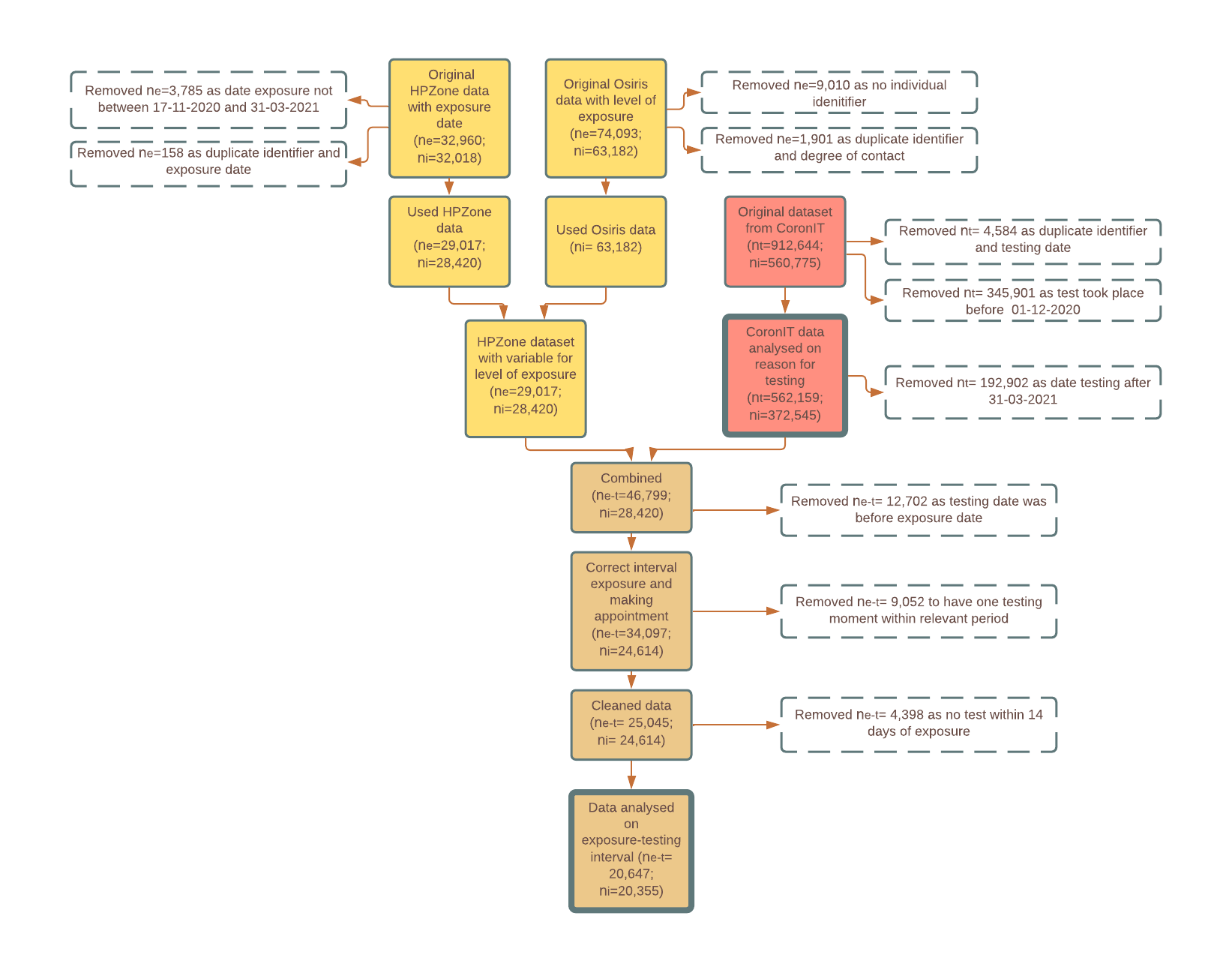
 Abbreviations: n_e_= exposure, n_t_= test, n_e-t_= interval exposure to testing, n_i_= individual

## 
