# Supplementary material for: The epidemiological impact of digital and manual contact tracing on the SARS-CoV-2 epidemic in the Netherlands: Empirical evidence
Source: PLOS Digit Health. 2023 Dec 29;2(12):e0000396. doi: 10.1371/journal.pdig.0000396 (PMC10756539; doi:10.1371/journal.pdig.0000396)
Supplement: S3 Fig — (DOCX) [file pdig.0000396.s006.docx]

## Figure S3: Questionnaires used in the first RDT and second RDT studies

| **A: First RDT study (asymptomatic individuals)^1^** | **B. Second RDT study^2^** |
| --- | --- |
| 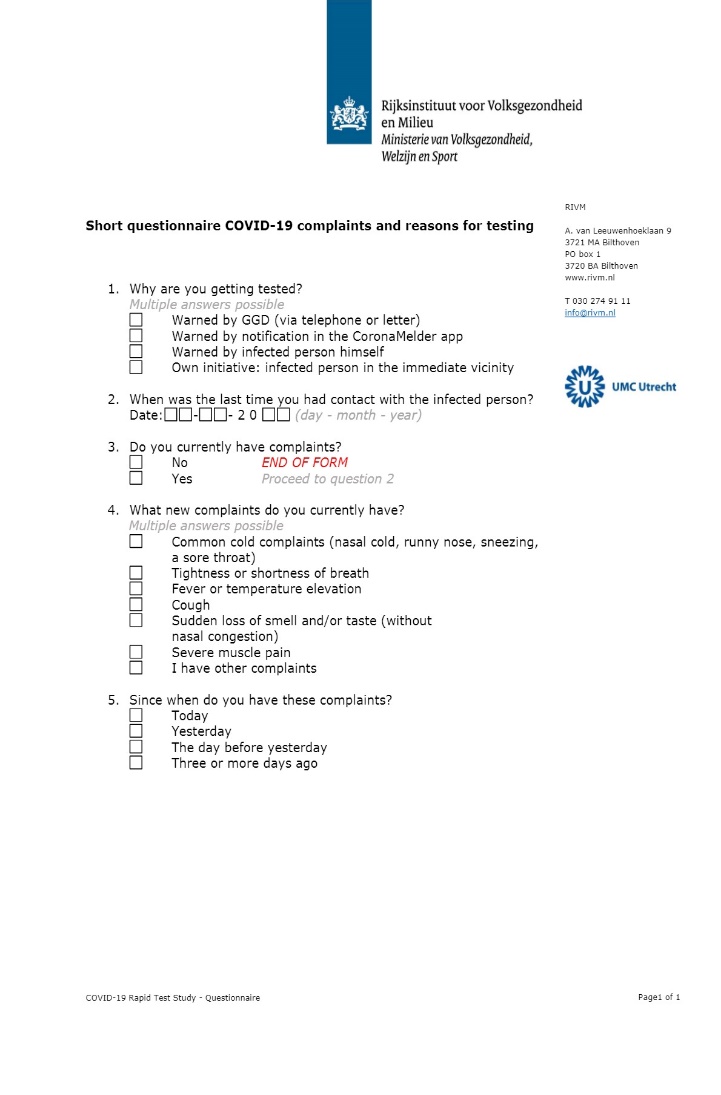 | 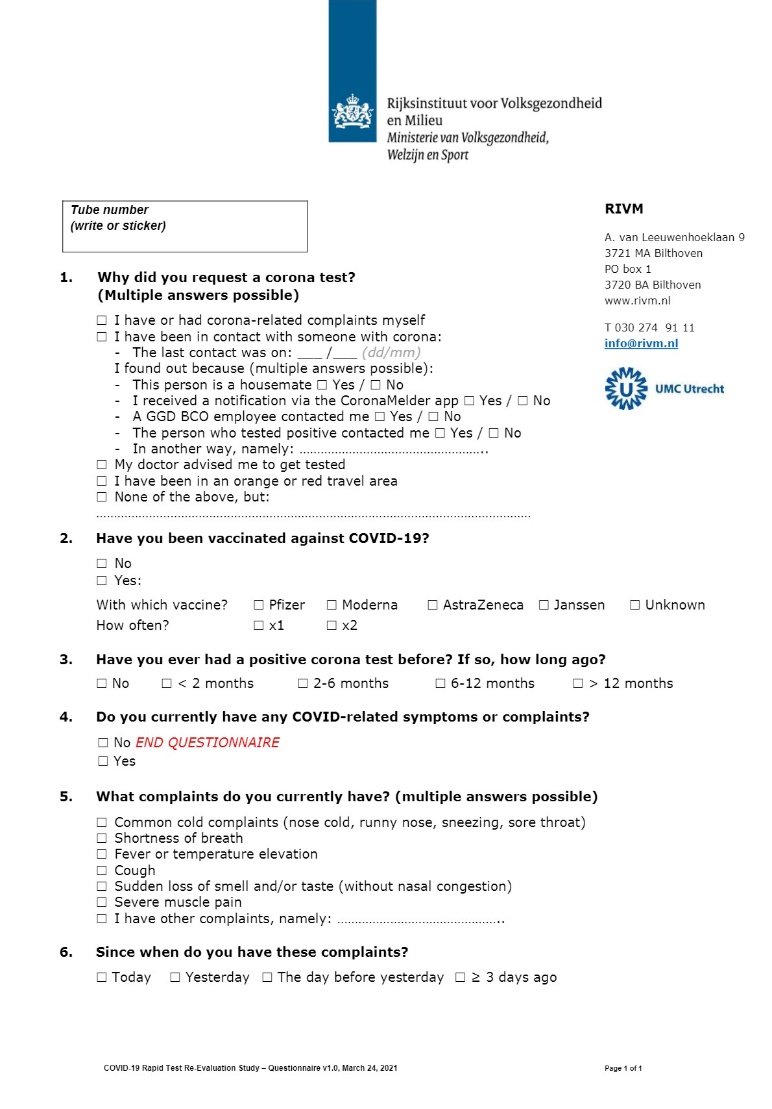 |

Abbreviations: RDT=rapid diagnostic test

1. The study period is 14 December 2020- 6 February 2021.
2. The study period is 12 April- 14 June 2021.
