# Supplementary material for: The epidemiological impact of digital and manual contact tracing on the SARS-CoV-2 epidemic in the Netherlands: Empirical evidence
Source: PLOS Digit Health. 2023 Dec 29;2(12):e0000396. doi: 10.1371/journal.pdig.0000396 (PMC10756539; doi:10.1371/journal.pdig.0000396)
Supplement: S4 Fig — (DOCX) [file pdig.0000396.s007.docx]

## Figure S4: Participant flow diagrams first and second RDT studies

| **A: First RDT study (asymptomatic close contacts)^1^** |
| --- |
| 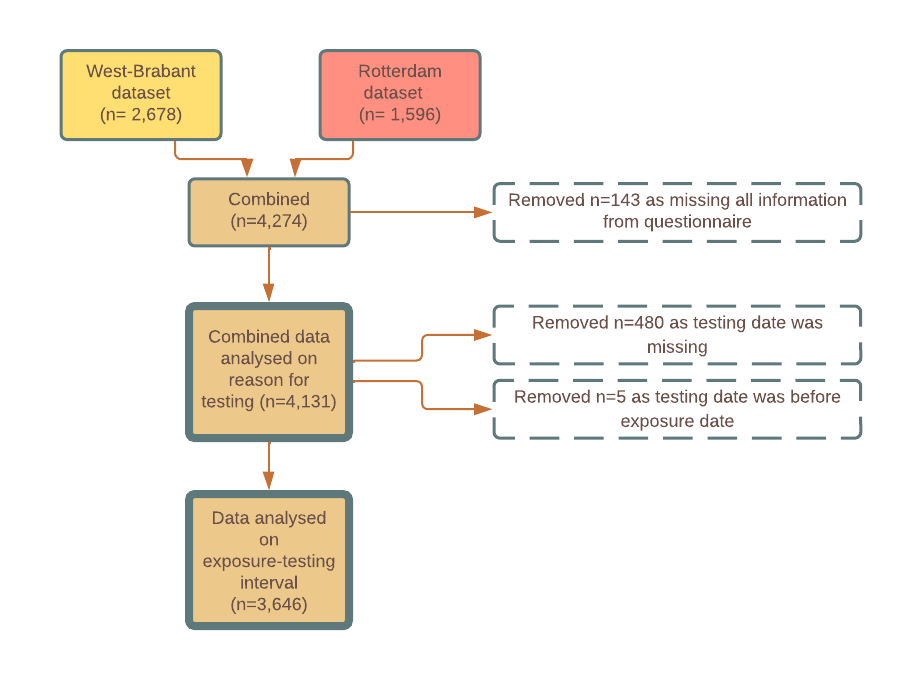 |
| **B. Second RDT study^2^** |
| 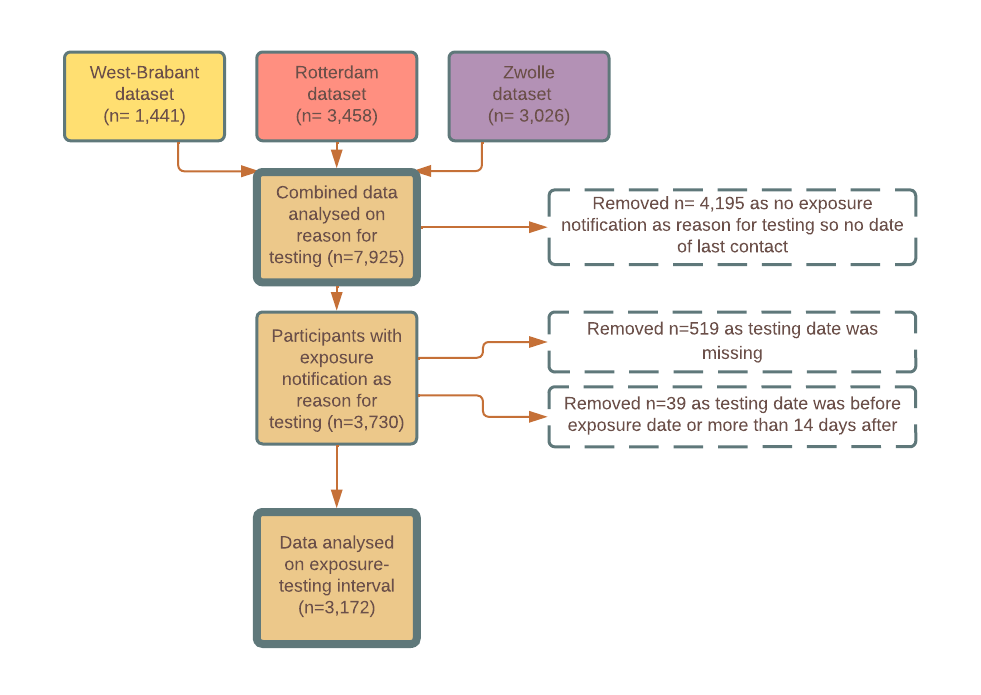 |

Abbreviations: RDT=rapid diagnostic test.

1. The study period was 14 December 2020 - 6 February 2021.
2. The study period was 12 April - 14 June 2021. Only participants who reported a close contact were asked the date of last exposure, and some dates were missing or incorrectly reported (e.g. before the testing date or more than 14 days after the testing date).
