# Supplementary material for: The epidemiological impact of digital and manual contact tracing on the SARS-CoV-2 epidemic in the Netherlands: Empirical evidence
Source: PLOS Digit Health. 2023 Dec 29;2(12):e0000396. doi: 10.1371/journal.pdig.0000396 (PMC10756539; doi:10.1371/journal.pdig.0000396)
Supplement: S1 Table — (DOCX) [file pdig.0000396.s008.docx]

## Table S1: Test population characteristics by reason for testing – PHS Amsterdam

|  | **DCT**  n_t_= 8,231  (1.46%) | **MCT**  n_t_= 27,513  (4.89 %) | **Symptoms**  n_t_= 394,408  (70.16%) | **Unknown**  n_t_= 132,007  (23.48%) | **Total^1^**  n_t_= 562,159 | **p-value^2^** |
| --- | --- | --- | --- | --- | --- | --- |
| **Median age in years^3^** *(IQR)*  *[Range]* | 35  (27- 51)  [0-91] | 27  (12- 45)  [0-100] | 33  (24- 45)  [0-99] | 31  (21- 47)  [0-98] | 32  (23- 46)  [0-100] | <0.01 |
| **Gender^3^**  *Female,* n_t_ *(%)* | 4,467 (54.33) | 14,627 (53.25) | 218,795 (55.57) | 68,994 (52.44) | 306,883 (54.70) | <0.01 |
| **Municipality^3^**  *Amsterdam,* n_t_ *(%)*  *Aalsmeer,* n_t_ *(%)*  *Amstelveen,* n_t_ *(%)*  *Diemen,* n_t_ *(%)*  *Ouder-Amstel,* n_t_ *(%)*  *Uithorn,* n_t_ *(%)* | 6,782 (82.80)  232 (2.83)  604 (7.37)  224 (2.73)  127 (1.55)  222 (2.71) | 21,339 (77.77)  1,339 (4.88)  2,491 (9.08)  781 (2.85)  519 (1.89)  971 (3.54) | 329,544 (84.00)  9,912 (2.53)  28,238 (7.20)  10,204 (2.60)  5,167 (1.32)  9,232 (2.35) | 100,280 (76.25)  5,760 (4.38)  13,612 (10.35)  4,763 (3.62)  2,335 (1.78)  4,764 (3.62) | 457,945 (81.86)  17,243 (3.08)  44,945 (8.03)  15,972 (2.85)  8,148 (1.46)  15,189 (2.72) | <0.01 |
| **Symptoms**  *Yes,* n_t_ *(%)* | 2,940 (35.72) | 8,619 (31.33) | 394,408 (100) | 0 (0) | 405,967 (72.22) | <0.01 |
| **Test result^4^**  *Positive,* n_t_ *(%)* | 498 (6.09) | 4,821 (17.62) | 36,308 (9.26) | 9,895 (7.54) | 51,522 (9.22) | <0.01 |

Abbreviations: DCT=digital contact tracing; MCT=manual contact tracing; IQR=interquartile range

1. Includes 562,159 tests (n_t_) by 372,545 individuals (n_i_) between 1 December 2020 – 31 May 2021. The reason for testing categories are based on a hierarchy as explained in the methods. Missing values for test result (n_t_ = 3,480), age (n_t_ = 196), gender (n_t_ = 1,130), and municipality (n_t_ = 2,717).
2. Pearson’s Chi-squared for categorical variables and Kruskal-Wallis for continuous variables to determine differential distribution across reasons for testing. For further analysis in case of a statistically significant difference, see methods.
3. Some differences between groups were statistically significant but these differences were considered to be not meaningful.
4. The test positivity percentage was statistically significantly lower among those testing after a DCT notification or for an unknown reason, and higher after a MCT notification.
