# Supplementary material for: The epidemiological impact of digital and manual contact tracing on the SARS-CoV-2 epidemic in the Netherlands: Empirical evidence
Source: PLOS Digit Health. 2023 Dec 29;2(12):e0000396. doi: 10.1371/journal.pdig.0000396 (PMC10756539; doi:10.1371/journal.pdig.0000396)
Supplement: S2 Table — (DOCX) [file pdig.0000396.s009.docx]

## Table S2: Test population characteristics by reason for testing – first RDT study (asymptomatic close contacts)

|  | **DCT**  (n= 293, 7.09%) | **MCT**  (n= 507, 12.27%) | **Index**  (n= 2,409, 58.32%) | **Self**  (n= 479, 11.60%) | **Unknown**  (n= 443, 10.72%) | **Total^1^**  (n= 4,131) | **p^2^** |
| --- | --- | --- | --- | --- | --- | --- | --- |
| **Median age in years^3^** *(IQR)*  *[Range]* | 50  (35- 61)  [16-82] | 45  (28- 57)  [16-86] | 42  (27- 56)  [16-96] | 48  (32- 58)  [16-89] | 42  (27- 56)  [16-94] | 44  (28- 57)  [16-96] | <0.01 |
| **Gender**  *Female, n (%)* | 140 (47.95) | 245 (48.32) | 1,208 (50.38) | 219 (46.01) | 236 (53.39) | 2,048 (49.77) | 0.19 |
| **Test region^4^**  *West-Brabant*  *Rotterdam* | 206 (70.55)  86 (29.45) | 362 (71.40)  145 (28.60) | 1,286 (53.54)  1,116 (46.46) | 361 (75.68)  116 (24.32) | 328 (74.04)  115 (25.96) | 2,543 (61.71)  1,578 (38.29) | <0.01 |
| **Symptoms^5^**  *Yes, n (%)* | 13 (4.47) | 24 (4.75) | 205 (8.57) | 70 (14.71) | 65 (14.71) | 377 (9.18) | <0.01 |
| **Test result^6^**  *Positive, n (%)* | 10 (3.41) | 50 (9.86) | 196 (8.14) | 53 (11.06) | 40 (9.03) | 349 (8.45) | <0.01 |
| ***Median Ct-values^,7^***  *(IQR)*  *Ct≤30, n (%)*  *Ct>30, n (%)* | 23.42  (21.91- 28.47)  8 (80.00)  2 (20.00) | 25.10  (20.22- 30.27)  37 (74.00)  13 (26.00) | 24.11  (20.74-32.24)  139 (70.92)  57 (29.08) | 25.10  (21.24-28.87)  41 (77.36)  12 (22.64) | 27.17  (22.60-32.20)  23 (57.50)  17 (42.50) | 24.90  (20.99-31.32)  248 (71.06)  101 (28.94) | 0.45 |

Abbreviations: Ct=Cycle threshold; DCT=digital contact tracing; Index=a person who tested SARS-CoV-2 positive; IQR=interquartile range; MCT=manual contact tracing; Self=testing at one’s own initiative.

1. Includes 4,131 tests by 4,131 participants between 14 December 2020- 6 February 2021. The reason for testing categories are based on a hierarchy as explained in the methods. Missing values for symptoms (n= 25), age (n= 10), gender (n= 16), and test location (n= 10).
2. Pearson’s Chi-squared for categorical variables and Kruskal-Wallis for continuous variables to determine differential distribution across reasons for testing. For further analysis in case of a statistically significant difference, see methods.
3. Some of the median ages were statistically significantly different between groups but these differences were considered to be not meaningful.
4. Among individuals testing because of an index notification, the proportion testing in West Brabant was statistically significantly lower as compared to the group average. This proportion was statistically significantly higher for all other reasons for testing. The reverse was true for Rotterdam.
5. Symptoms were present in a statistically significantly lower percentage of those testing after a DCT or MCT notification and a statistically significantly higher percentage in the Self and Unknown reason groups.
6. Test positivity was statistically significantly lower among those testing after a DCT notification.
7. Only the Ct-value of participants (n=349) with a positive test result were included (by definition, the Ct value is 45 in those testing negative). The Ct 30 cut-off is often used as a proxy of infectiousness.
