# Supplementary material for: The epidemiological impact of digital and manual contact tracing on the SARS-CoV-2 epidemic in the Netherlands: Empirical evidence
Source: PLOS Digit Health. 2023 Dec 29;2(12):e0000396. doi: 10.1371/journal.pdig.0000396 (PMC10756539; doi:10.1371/journal.pdig.0000396)
Supplement: S3 Table — (DOCX) [file pdig.0000396.s010.docx]

## Table S3: Test population characteristics by reason for testing – second RDT study

|  | **DCT**  (n= 192;  2.42%) | **MCT**  (n= 152;  1.92%) | **Index**  (n= 1,495;  18.86%) | **Housemate**  (n= 880  11.10%) | **Self**  (n= 240  3.03%) | **Unknown notification**  (n= 771; 9.73%) | **Symptoms**  (n= 3,586; 45.25%) | **Other**  (n=315  3.97%) | **Unknown reason**  (n=294; 3.71%) | **Total^1^**  (n=7,925) | **p-value^2^** |
| --- | --- | --- | --- | --- | --- | --- | --- | --- | --- | --- | --- |
| **Median age in years^3^** *(IQR)*  *[Range]* | 40  (26- 51)  [18- 84] | 33  (24- 53)  [16-78] | 31  (24- 46)  [16-82] | 33  (23- 50)  [16-82] | 38  (26- 51)  [16-73] | 37  (25- 54)  [16-91] | 37  (28- 48)  [16-88] | 46  (31- 62)  [17-84] | 43  (29- 60)  [17-84] | 36  (26- 50)  [16-91] | <0.01 |
| **Gender^3^**  *Female,* n_t_ *(%)* | 101 (53.16) | 82 (54.30) | 756 (50.74) | 463 (52.67) | 138 (57.50) | 362 (47.07) | 1,914 (53.54) | 137 (43.49) | 143 (48.81) | 4,096 (51.83) | <0.01 |
| **Test region^4^**  *West-Brabant*  *Rotterdam*  *Zwolle* | 51 (26.56)  88 (45.83)  53 (27.60) | 8 (5.26)  43 (28.29)  101 (66.45) | 380 (25.42)  687 (45.95)  428 (28.63) | 211 (23.98)  436 (49.55)  233 (26.48) | 72 (30.00)  92 (38.33)  76 (31.67) | 230 (29.83)  405 (52.53)  136 (17.64) | 371 (10.35)  1464 (40.83)  1751 (48.83) | 55 (17.46)  128 (40.63)  132 (41.90) | 63 (21.43)  115 (39.12) 116 (39.46) | 1,441 (18.18)  3,458 (43.63)  3,026 (38.18) | <0.01 |
| **Symptoms^5^**  *Yes,* n *(%)* | 38 (19.79) | 43 (28.29) | 471 (31.59) | 287 (32.76) | 73 (30.80) | 242 (31.59) | 3,586 (100.0) | 130 (41.53) | 62 (55.36) | 4,932 (62.23) | <0.01 |
| **Test result^6^**  *Positive,* n *(%)* | 7 (3.65) | 11 (7.24) | 140 (9.36) | 201 (22.84) | 22 (9.17) | 105 (13.62) | 241 (6.72) | 17 (5.40) | 29 (9.86) | 773 (9.75) | <0.01 |
| ***Median Ct-values^7^***  *(IQR)*  *Ct≤30, n (%)*  *Ct>30, n (%)* | 20.89  (20.05-24.07)  7 (100.00)  0 (0) | 22.00  (18.70-27.20)  9 (100.00)  0 (0) | 23.64  (20.79-29.57)  103 (76.87)  31 (23.13) | 23.14  (20.50-29.00)  161 (82.56)  32 (17.44) | 22.69  (19.15-28.53)  19 (86.36)  3 (13.64) | 22.80  (20.60-27.74)  84 (81.55)  19 (18.45) | 21.96  (19.70-25.85)  206 (88.79)  26 (11.21) | 27.13  (22.40-34.40)  11 (64.71)  6 (35.29) | 26.22  (22.22-31.02)  19 (67.86)  9 (32.14) | 22.95  (20.30-27.90)  619 (82.86)  128 (17.14) | <0.01 |
| **Vaccinated^3^**  *Yes,* n *(%)* | 34 (17.71) | 36 (23.68) | 128 (8.56) | 87 (9.91) | 32 (13.33) | 105 (13.64) | 414 (11.55) | 67 (21.27) | 37 (33.64) | 940 (12.15) | <0.01 |
| **Prior infection**  *Yes,* n *(%)* | 13 (6.77) | 17 (11.18) | 150 (10.06) | 72 (8.23) | 14 (5.86) | 68 (8.91) | 292 (8.16) | 36 (11.50) | 14 (12.61) | 676 (8.76) | 0.07 |

Abbreviations: Ct=Cycle threshold; DCT=digital contact tracing; Index=a person who tested SARS-CoV-2 positive; IQR=interquartile range; MCT=manual contact tracing; Self=testing at one’s own initiative.

1. Includes 7,925 tests by 7,925 participants between 12 April- 14 June 2021. The reason for testing categories are based on a hierarchy as explained in the methods. Missing values for symptoms (n=200), Ct rounds (n=26), age (n=19), gender (n=23), vaccination status (n=190), and prior SARS-CoV-2 infection (n= 210).
2. Pearson’s Chi-squared for categorical and Kruskal-Wallis for continuous variables to determine differential distribution across reasons for testing. For further analysis in case of a statistically significant difference, see methods.
3. Some differences between the groups were statistically significant but these differences were considered to be not meaningful.
4. Among individuals testing because of a MCT notification, the proportion testing in Zwolle was statistically significantly higher, and in West-Brabant lower. Among individuals testing because of an index notification, the proportion testing in Zwolle was statistically significantly lower and in West-Brabant higher. Among individuals testing because of a housemate or unknown notification, the proportion testing in Zwolle was statistically significantly lower and in Rotterdam and Brabant higher. Among individuals testing because of having symptoms, the proportion testing in Zwolle was statistically significantly higher and in Rotterdam and Brabant lower.
5. Includes either symptoms as reason for testing or presence of symptoms. The statistically significant difference among the groups is not meaningful due to the group testing based on symptoms.
6. Test positivity was statistically significantly higher after a notification from a housemate or an unknown notification, and lower among those testing based on symptoms.
7. Only the Ct-value of participants with a positive test result were included (by definition, the Ct value is 45 in those testing negative). The Ct 30 cut-off is often used as a proxy of infectiousness. The median Ct-value was significantly different in the Index group compared to the Symptoms group but this difference was considered not meaningful.
