# Supplementary material for: The epidemiological impact of digital and manual contact tracing on the SARS-CoV-2 epidemic in the Netherlands: Empirical evidence
Source: PLOS Digit Health. 2023 Dec 29;2(12):e0000396. doi: 10.1371/journal.pdig.0000396 (PMC10756539; doi:10.1371/journal.pdig.0000396)
Supplement: S4 Table — (DOCX) [file pdig.0000396.s011.docx]

## Table S4: Mean (SD) interval in days between first exposure and testing – PHS Amsterdam MCT subset

|  | **DCT**  (n_e-t_= 297;  1.44%) | **n_e-t_ included**  **(%)** | **MCT**  (n_e-t_= 6,624;  32.08%) | **n_e-t_ included**  **(%)** | **Symptoms**  (n_e-t_= 6,684;  32.37%) | **n_e-t_ included**  **(%)** | **Unknown**  (n_e-t_= 7,042; 34.11%) | **n_e-t_ included**  **(%)** | **Total^1^**  (n_e-t_=20,647) | **Total n_e-t_ included**  **(%)** |
| --- | --- | --- | --- | --- | --- | --- | --- | --- | --- | --- |
| **All:** *Mean (SD)* | 4.22 (2.71) |  | 4.49 (2.36) |  | 3.09 (2.71) |  | 4.11 (2.47) |  | 3.91 (2.59) |  |
| **Age in years:** *0-14*  *15-29*  *30-44*  *45-59*  *60+* | 5.22 (4.29)  4.12 (2.19)  4.04 (2.76)  4.13 (2.97)  4.65 (2.78) | 9 (3)  90 (30)  77 (26)  75 (25)  46 (15) | 4.54 (2.52)  4.54 (2.21)  4.41 (2.41)  4.42 (2.34)  4.57 (2.23) | 1,801 (27)  1,601 (24)  1,232 (19)  1,398 (21)  592 (9) | 3.42 (2.91)  3.06 (2.53)  2.96 (2.71)  2.98 (2.92)  3.35 (2.71) | 840 (13)  2,616 (39)  1,400 (21)  1,308 (20)  520 (8) | 4.42 (2.39)  3.93 (2.40)  4.04 (2.54)  3.87 (2.53)  4.37 (2.54) | 1,870 (27)  1,941 (28)  1,163 (17)  1,346 (19)  722 (10) | 4.29 (2.58)  3.72 (2.48)  3.77 (2.65)  3.78 (2.67)  4.16 (2.55) | 4,520 (22)  6,248 (30)  3,872 (19)  4,127 (20)  1,880 (9) |
| **Gender:** *Female*  *Male* | 4.17 (2.62)  4.28 (2.83) | 162 (55) 135 (45) | 4.44 (2.35)  4.55 (2.38) | 3,487 (53)  3,125 (47) | 3.05 (2.65)  3.13 (2.79) | 3,694 (55)  2,977 (45) | 4.11 (2.43)  4.13 (2.51) | 3,587 (51)  3,433 (49) | 3.86 (2.55)  3.96 (2.63) | 10,930 (53)  9,670 (47) |
| **Municipality:**  *Amsterdam*  *Aalsmeer*  *Amstelveen*  *Diemen*  *Ouder-Amstel*  *Uithorn* | 4.28 (2.75)  4.76 (2.19)  3.66 (2.33)  2.50 (1.52)  4.38 (3.54)  4.42 (3.13) | 218 (73)  17 (6)  29 (10)  6 (2)  8 (3)  19 (6) | 4.54 (2.38)  4.61 (2.17)  4.15 (2.25)  4.84 (2.40)  3.95 (2.16)  4.31 (2.53) | 5,091 (77) 323 (5)  635 (10)  220 (3)  129 (2)  226 (3) | 3.12 (2.72)  2.43 (2.46)  3.02 (2.64)  3.02 (2.65)  3.51 (2.92)  2.83 (2.95) | 5,497 (82)  181 (3)  528 (8)  196 (3)  107 (2)  175 (3) | 4.14 (2.45)  3.71 (2.62)  4.18 (2.44)  3.88 (2.55)  3.90 (2.12)  4.28 (2.82) | 5,461 (78)  291 (4)  680 (10)  246 (3)  129 (2)  235 (3) | 3.92 (2.6)  3.81 (2.54)  3.83(2.49)  3.93(2.63)  3.82(2.42)3.91(2.83) | 16,267 (79)  812 (4)  1,872 (9)  668 (3)  373 (2)  655 (3) |
| **Type of contact:^2^**  *Household*  *Close, long*  *Close, short*  *Other contact*  *Case* | 4.41 (3.41)  4.78 (1.59)  4.67 (1.37)  3.00 (1.41)  3.33 (2.61) | 109 (37) 93 (32)  6 (2)  2 (1)  85 (29) | 4.54 (2.58)  5.16 (1.51)  5.22 (1.51)  5.29 (0.99)  3.59 (2.51) | 2,982 (45)  1,920 (29)  68 (1)  14 (0)  1,622 (25) | 2.78 (2.93)  3.89 (2.21)  3.90 (1.96)  2.69 (2.02)  2.70 (2.75) | 2,254 (34)  1,939 (29)  72 (1)  13 (0)  2,382 (36) | 3.88 (2.74)  4.88 (1.70)  5.35 (1.36)  4.85 (1.82)  3.18 (2.67) | 2,825 (40)  2,583 (37)  89 (1)  13 (0)  1,507 (21) | 3.83 (2.84)  4.67 (1.89) 4.85 (1.72)  4.24 (1.96)  3.09 (2.68) | 8,170 (40)  6,535 (32)  235 (1)  42 (0)  5,596 (27) |
| **Symptoms:** *No*  *Yes* | 4.98 (2.53)  3.40 (2.67) | 154 (52)  143 (48) | 4.85 (2.15)  3.55 (2.62) | 4,796 (72)  1,828 (28) | ---  3.09 (2.71) | 0 (0)  6,684 (100) | 4.11 (2.47)  --- | 7,042 (100)  0 (0) | 4.42 (2.38)  3.19 (2.70) | 11,992 (58)  8,655 (42) |
| **Test result:** *Negative*  *Positive* | 4.45 (2.74)  3.55 (2.53) | 222 (75)  75 (25) | 4.72 (2.28)  3.71 (2.49) | 5,104 (77)  1,493 (23) | 3.17 (2.68)  2.92 (2.79) | 4,552 (68)  2,107 (32) | 4.23 (2.41)  3.54 (2.66) | 5,790 (83)  1,226 (17) | 4.09 (2.53)  3.33 (2.69) | 15,668 (76)  4,901 (24) |

Abbreviations: DCT=digital contact tracing; MCT=manual contact tracing; SD= standard deviation

1. Includes 20,647 exposure- testing intervals (n_e-t_) by 20,355 individuals (n_i_) between 1 December 2020- 31 March 2021. Missing values for type of contact (n_e-t_= 69), test result (n_e-t_= 78), and gender (n_e-t_= 47).
2. “Close” is defined as within 1.5 meters of an infectious person; “long” as more than 15 minutes; “short” as 15 minutes or less but with high intensity (e.g. coughing in someone’s face, kissing); “household” as a close contact within the same residence; and “other” as any other contact with an infectious person.
