# Supplementary material for: The epidemiological impact of digital and manual contact tracing on the SARS-CoV-2 epidemic in the Netherlands: Empirical evidence
Source: PLOS Digit Health. 2023 Dec 29;2(12):e0000396. doi: 10.1371/journal.pdig.0000396 (PMC10756539; doi:10.1371/journal.pdig.0000396)
Supplement: S5 Table — (DOCX) [file pdig.0000396.s012.docx]

## Table S5: Mean (SD) interval in days between last exposure and testing – first RDT study (asymptomatic close contacts)

|  | **DCT**  (n= 285;  7.82%) | **n_e-t_ included**  **(%)** | **MCT**  (n= 503;  13.80%) | **n_e-t_ included**  **(%)** | **Index**  (n= 2,389;  65.52%) | **n_e-t_ included**  **(%)** | **Self**  (n= 444;  12.18% | **n_e-t_ included**  **(%)** | **Unknown**  (n= 25;  0.69%) | **n_e-t_ included**  **(%)** | **Total^1^**  (n= 3,646) | **n_e-t_ included**  **(%)** |
| --- | --- | --- | --- | --- | --- | --- | --- | --- | --- | --- | --- | --- |
| **All**  *Mean (SD)* | 5.17 (1.39) |  | 5.09 (1.44) |  | 4.96 (1.34) |  | 4.49 (2.20) |  | 4.16 (2.67) |  | 4.93 (1.51) |  |
| **Age in years**  *16-29*  *30-44*  *45-59*  *60+* | 5.14 (1.54)  5.15 (0.90)  5.02 (1.31)  5.39 (1.75) | 42 (15)  71 (25)  95 (33)  76 (27) | 5.12 (1.35)  5.00 (1.33)  5.07 (1.45)  5.17 (1.68) | 135 (27)  114 (23)  151 (30)  103 (20) | 4.91 (1.25)  5.10 (1.36)  4.83 (1.38)  5.06 (1.37) | 704 (30)  565 (24)  672 (28)  441 (19) | 4.87 (2.03)  4.29 (2.18)  4.51 (2.36)  4.27 (2.13) | 98 (22)  91 (21)  160 (36)  94 (21) | 4.29 (3.30)  4.33 (1.15)  4.60 (2.88)  3.80 (2.74) | 7 (28)  3 (12)  5 (20)  10 (40) | 4.94 (1.39)  5.00 (1.46)  4.83 (1.58)  4.99 (1.63) | 986 (27)  844 (23)  1,083 (30)  724 (20) |
| **Gender**  *Female*  *Male* | 5.31 (1.22)  5.05 (1.54) | 137 (48)  147 (52) | 5.12 (1.39)  5.05 (1.49) | 245 (49)  258 (51) | 4.99 (1.27)  4.93 (1.41) | 1,194 (50)  1,184 (50) | 4.41 (2.26)  4.55 (2.16) | 204 (46)  238 (54) | 3.56 (2.79)  4.50 (2.63) | 9 (36)  16 (64) | 4.96 (1.46)  4.90 (1.57) | 1,789 (49)  1,843 (51) |
| **Test location**  *West-Brabant*  *Rotterdam* | 5.17 (1.58)  5.19 (0.82) | 199 (70)  85 (30) | 5.15 (1.54)  4.92 (1.14) | 358 (71)  145 (29) | 4.94 (1.49)  4.98 (1.15) | 1,272 (53)  1,110 (47) | 4.38 (2.39)  4.85 (1.47) | 336 (76)  107 (24) | 4.59 (2.50)  1.00 (1.73) | 22 (88)  3 (12) | 4.90 (1.70)  4.97 (1.17) | 2,187 (60)  1,450 (40) |
| **Symptoms**  *No*  *Yes* | 5.19 (1.42)  4.85 (0.90) | 270 (95)  13 (5) | 5.09 (1.46)  4.91 (1.12) | 479 (95)  23 (5) | 5.00 (1.25)  4.58 (2.01) | 2,174 (92)  199 (8) | 4.51 (2.12)  4.35 (2.73) | 388 (88)  55 (12) | 4.71 (2.37)  1.67 (2.89) | 21 (88)  3 (12) | 4.97 (1.44)  4.54 (2.10) | 3,332 (92)  293 (8) |
| **Test result**  *Negative*  *Positive* | 5.15 (1.41)  5.70 (0.95) | 275 (96)  10 (4) | 5.13 (1.29)  4.70 (2.39) | 453 (90)  50 (10) | 4.99 (1.30)  4.57 (1.72) | 2,195 (92)  194 (8) | 4.53 (2.18)  4.16 (2.37) | 395 (89)  49 (11) | 4.13 (2.78)  4.50 (0.71) | 23 (92)  2 (8) | 4.96 (1.46)  4.56 (1.95) | 3,341 (92)  305 (8) |

Abbreviations: DCT=digital contact tracing; Index=a person who tested SARS-CoV-2 positive; MCT=manual contact tracing; SD=standard deviation; Self=testing at one’s own initiative.

1. Includes 3,646 exposure-test intervals in 3,646 participants between 14 December 2020- 6 February 2021. Only participants who reported a close contact were asked the date of last exposure and dates are missing (n=480) or not logical (before testing date or more than 14 days after testing, n=5). Additional missing values for symptoms (n=21), age (n=9), gender (n=14), and test location (n=9).
