# Supplementary material for: The epidemiological impact of digital and manual contact tracing on the SARS-CoV-2 epidemic in the Netherlands: Empirical evidence
Source: PLOS Digit Health. 2023 Dec 29;2(12):e0000396. doi: 10.1371/journal.pdig.0000396 (PMC10756539; doi:10.1371/journal.pdig.0000396)
Supplement: S6 Table — (DOCX) [file pdig.0000396.s013.docx]

## Table S6: Mean (SD) interval in days between last exposure and testing – second RDT study

|  | **DCT**  (n= *153;*  *4.82*%) | **n**  **(%)** | **MCT**  (n= 139;  4.38%) | **n**  **(%)** | **Index** (n=1,419;  44.74%) | **n**  **(%)** | **Housemate**  (n=795;  25.06%) | **n**  **(%)** | **Self**  (n=147;  4.63%) | **n**  **(%)** | **Unknown** (n=519; 16.36%) | **n**  **(%)** | **Total^1^**  (n=3,172) | **Total n**  **(%)** |
| --- | --- | --- | --- | --- | --- | --- | --- | --- | --- | --- | --- | --- | --- | --- |
| **All**  *Mean (SD)* | 4.84 (1.59) |  | 5.11 (1.68) |  | 4.70 (1.52) |  | 3.12 (2.53) |  | 4.94 (1.90) |  | 4.47 (1.93) |  | 4.30 (2.04) |  |
| **Symptoms**  *No*  *Yes* | 4.74 (1.43)  5.22 (2.09) | 121 (79)  32 (21) | 4.94 (1.10)  5.64 (2.78) | 106 (76)  33 (24) | 4.75 (1.42)  4.60 (1.73) | 970 (69)  445 (31) | 3.44 (2.49)  2.47 (2.49) | 528 (67)  263 (33) | 5.15 (1.69)  4.62 (2.21) | 93 (64)  52 (36) | 4.66 (1.85)  4.03 (2.04) | 361 (70)  155 (30) | 4.44 (1.89)  4.00 (2.30) | 2,179 (69)  980 (31) |
| **Test result**  *Negative*  *Positive* | 4.83 (1.62)  5.14 (0.90) | 146 (95)  7 (5) | 5.06 (1.64)  5.88 (2.10) | 131 (94)  8 (6) | 4.72 (1.49)  4.53 (1.84) | 1,284 (90)  135 (10) | 3.29 (2.52)  2.54 (2.51) | 615 (77)  180 (23) | 4.95 (1.81)  4.87 (2.64) | 132 (90)  15 (10) | 4.63 (1.84)  3.39 (2.22) | 455 (88)  64 (12) | 4.42 (1.94)  3.52 (2.44) | 2,763 (87)  409 (13) |
| **Age in years**  *16-29*  *30-44*  *45-59*  *60+* | 4.81 (1.66)  4.75 (1.38)  5.03 (1.93)  4.82 (1.13) | 57 (37)  44 (29)  35 (23)  17 (11) | 4.84 (1.13)  6.29 (2.54)  4.35 (1.58)  5.17 (0.96) | 64 (46)  28 (20)  23 (17)  24 (17) | 4.58 (1.47)  4.87 (1.58)  4.76 (1.64)  4.71 (1.39) | 678 (48)  365 (26)  238 (17)  133 (9) | 3.52 (2.30)  2.29 (2.80)  3.13 (2.58)  2.64 (2.53) | 378 (48)  159 (20)  215 (27)  42 (5) | 5.16 (1.91)  4.89 (1.81)  4.59 (1.80)  5.09 (2.21) | 50 (34)  37 (25)  37 (25)  23 (16) | 4.52 (1.91)  4.16 (1.99)  4.51 (1.90)  5.04 (1.83) | 224 (43)  135 (26)  111 (21)  49 (9) | 4.34 (1.87)  4.26 (2.27)  4.18 (2.18)  4.54 (1.88) | 1,451 (46)  768 (24)  659 (21)  288 (9) |
| **Gender**  *Female*  *Male* | 4.96 (1.71)  4.70 (1.48) | 81 (54)  70 (46) | 5.09 (1.56)  5.13 (1.83) | 78 (56)  61 (44) | 4.77 (1.44)  4.63 (1.60) | 725 (51)  689 (49) | 3.12 (2.54)  3.13 (2.53) | 419 (53)  375 (47) | 4.93 (1.79)  4.95 (2.08) | 90 (61)  57 (39) | 4.65 (1.80)  4.32 (2.04) | 244 (47)  274 (53) | 4.36 (2.01)  4.24 (2.07) | 1,637 (52)  1,526 (48) |
| **Testing region**  *West-Brabant*  *Rotterdam*  *Zwolle* | 5.16 (1.00)  4.60 (1.92)  4.90 (1.48) | 43 (28)  68 (44)  42 (27) | 5.71 (2.93)  4.98 (1.87)  5.12 (1.46) | 7 (5)  42 (30)  90 (65) | 4.90 (1.13)  4.69 (1.68)  4.54 (1.55) | 362 (26)  651 (46)  406 (29) | 3.46 (2.64)  3.19 (2.47)  2.69 (2.49) | 196 (25)  384 (48)  215 (27) | 5.17 (1.22)  5.27 (2.19)  4.11 (1.89) | 47(32)  62 (42)  38 (26) | 4.60 (1.59)  4.50 (1.94)  4.19 (2.35) | 164 (32)  252 (49)  103 (20) | 4.53 (1.82)  4.29 (2.11)  4.11 (2.09) | 819 (26)  1,459 (46)  894 (28) |
| **Vaccinated**  *No*  *Yes* | 4.84 (1.57)  4.86 (1.75) | 131 (86)  22 (14) | 5.07 (1.83)  5.22 (1.01) | 107 (77)  32 (23) | 4.69 (1.52)  4.77 (1.57) | 1,303 (92)  116 (8) | 3.09 (2.51)  3.35 (2.77) | 716 (90)  78 (10) | 4.97 (1.97)  4.72 (1.36) | 129 (88)  18 (12) | 4.41 (1.92)  5.00 (1.99) | 461 (89)  58 (11) | 4.28 (2.03)  4.52 (2.06) | 2,847 (90)  324 (10) |
| **Prior infection**  *No*  *Yes* | 4.82 (1.59)  5.09 (1.64) | 142 (93)  11 (7) | 5.08 (1.53)  5.33 (2.66) | 124 (89)  15 (11) | 4.71 (1.54)  4.60 (1.40) | 1,272 (90)  143 (10) | 3.09 (2.55)  3.34 (2.18) | 725 (92)  67 (8) | 5.07 (1.83)  2.62 (1.77) | 138 (95)  8 (5) | 4.43 (1.93)  4.90 (1.93) | 470 (91)  48 (9) | 4.29 (2.05)  4.36 (1.90) | 2,871 (91)  292 (9) |

Abbreviations: DCT=digital contact tracing; Index=a person who tested SARS-CoV-2 positive; MCT=manual contact tracing; SD=standard deviation; Self=testing at one’s own initiative.

Includes 3,172 exposure-test intervals in 3,172 participants between 12 April- 14 June 2021. Only participants who reported a close contact were asked the date of last exposure and dates are missing (n=519) or not logical (before testing date or more than 14 days after testing, n=39). Additional missing values for symptoms (n=13), age (n= 6), gender (n=9), vaccination status (n=1), and prior SARS-CoV-2 infection (n= 9).
