# Supplementary material for: The epidemiological impact of digital and manual contact tracing on the SARS-CoV-2 epidemic in the Netherlands: Empirical evidence
Source: PLOS Digit Health. 2023 Dec 29;2(12):e0000396. doi: 10.1371/journal.pdig.0000396 (PMC10756539; doi:10.1371/journal.pdig.0000396)
Supplement: S7 Table — (DOCX) [file pdig.0000396.s014.docx]

# Table S7: Tobit regression model for exposure-test intervals – PHS Amsterdam MCT subset

|  | **Univariable analysis^1^ (n_e-t_= 20,647)** | | | **Multivariable analysis^1^ (n_e-t_= 20,647)** | | |
| --- | --- | --- | --- | --- | --- | --- |
|  | **Coefficient^2^** | **95% CI** | **p-value** | **Coefficient^2^** | **95% CI** | **p-value** |
| **Age in years:** *0-14*  *15-29*  *30-44*  *45-59*  *60+* | 0.60  *reference*  0.01  0.03  0.45 | 0.49-0.71  ---  -0.11-0.12  -0.09-0.15  0.30-0.61 | <0.01  ---  0.92  0.61  <0.01 | 0.32  *reference*  0.01  0.11  0.34 | 0.21-0.43  ---  -0.10-0.12  0.00-0.22  0.19-0.48 | <0.01  ---  0.85  0.05  <0.01 |
| **Gender:** *Female*  *Male* | *reference*  0.11 | ---  0.03-0.19 | ---  <0.01 | *reference*  0.07 | ---  -0.01-0.15 | ---  0.07 |
| **Municipality:**  Amsterdam  Surrounding area | *reference*  -0.09 | ---  -0.18-0.01 | ---  0.09 | *reference*  -0.13 | ---  -0.23-(-) 0.04 | ---  <0.01 |
| **Type of contact:^3^**  *Household*  *Close, long*  *Close, short*  *Other contact*  *Case* | *reference*  1.01  1.22  0.56  -0.84 | ---  0.92-1.10  0.85-1.59  -0.31-1.42  -0.94-(-)0.74 | ---  <0.01  <0.01  0.21  <0.01 | *reference*  1.00  1.22  0.61  -0.57 | *---*  0.91-1.09  0.86-1.58  -0.24-1.45  -0.66-(-)0.47 | ---  <0.01  <0.01  0.16  <0.01 |
| **DCT:** *No*  *Yes* | *reference*  0.36 | ---  0.03-0.70 | ---  0.03 | *reference*  0.48 | ---  0.15-0.80 | ---  <0.01 |
| **Symptoms***: No*  *Yes* | *reference*  -1.39 | ---  -1.47-(-)1.31 | ---  <0.01 | *reference*  -1.17 | ---  -1.25-(-)1.09 | ---  <0.01 |
| **Test result:** *Negative*  *Positive* | *Reference*  -0.89 | ---  -0.99-(-)0.80 | ---  <0.01 | *Not included* | --- | --- |

Abbreviations: CI=confidence interval; DCT=digital contact tracing; MCT=manual contact tracing.

1. Based on 20,647 exposure- testing intervals (n_e-t_) by 20,355 individuals (n_i_) between 1 December 2020- 31 March 2021. Missing values for type of contact (n_e-t_= 69), test result (n_e-t_= 78), and gender (n_e-t_= 47).
2. The tobit coefficient represents the change in exposure-test interval (in days) for each unit change of the dependent variable.
3. “Close” is defined as within 1.5 meters of an infectious person; “long” as more than 15 minutes; “short” as 15 minutes or less but with high intensity (e.g. coughing in someone’s face, kissing); “household” as a close contact within the same residence; and “other” as any other contact with an infectious person.
