# Supplementary material for: The epidemiological impact of digital and manual contact tracing on the SARS-CoV-2 epidemic in the Netherlands: Empirical evidence
Source: PLOS Digit Health. 2023 Dec 29;2(12):e0000396. doi: 10.1371/journal.pdig.0000396 (PMC10756539; doi:10.1371/journal.pdig.0000396)
Supplement: S8 Table — (DOCX) [file pdig.0000396.s015.docx]

## Table S8: Tobit regression model for the exposure-test intervals – first RDT study

|  | **Univariable analysis^1^ (n= 3,646)** | | | **Multivariable analysis^1^ (n= 3,646)** | | |
| --- | --- | --- | --- | --- | --- | --- |
|  | **Coefficient^2^** | **95% CI** | **p-value** | **Coefficient^2^** | **95% CI** | **p-value** |
| **Age in years***: 16-29*  *30-44*  *45-59*  *60+* | *reference*  0.06  -0.11  0.05 | *---*  -0.08-0.21  -0.25- 0.02  -0.10-0.20 | ---  0.40  0.11  0.53 | *reference*  0.04  -0.10  0.06 | ---  -0.10-0.19  -0.24-0.03  -0.09-0.21 | ---  0.54  0.14  0.46 |
| **Gender:** *Female*  *Male* | *reference*  -0.06 | ---  -0.16-0.05 | ---  0.28 | *reference*  -0.05 | ---  -0.15-0.05 | ---  0.32 |
| **Testing region:** *Brabant*  *Rotterdam* | *reference*  *0.08* | ---  -0.02-0.19 | ---  0.12 | *reference*  0.05 | ---  -0.06-0.15 | ---  0.37 |
| **DCT:** *No*  *Yes* | *reference*  0.27 | ---  0.08-0.46 | ---  <0.01 | *reference*  0.08 | *---*  -0.16-0.32 | ---  0.51 |
| **MCT:** *No*  *Yes* | *reference*  0.18 | ---  0.04-0.33 | ---  0.01 | *reference*  0.04 | ---  -0.14-0.22 | ---  0.64 |
| **Index:** *No*  *Yes* | *reference*  0.15 | ---  0.04-0.27 | ---  <0.01 | *reference*  -0.14 | ---  -0.34-0.07 | ---  0.19 |
| **Self:** *No*  *Yes* | *reference*  -0.56 | ---  -0.71-(-)0.41 | ---  <0.01 | *reference*  -0.63 | ---  -0.87-0.39 | ---  <0.01 |
| **Unknown** *No*  **Contact:** *Yes* | *reference*  -0.88 | ---  -1.49- (-)0.26 | ---  <0.01 | *reference*  -0.83 | ---  -1.49-(-)0.18 | ---  0.01 |
| **Symptoms:** *No*  *Yes* | *reference*  -0.45 | ---  -0.63-(-)0.26 | ---  <0.01 | *reference*  -0.36 | ---  -0.55-(-)0.17 | ---  <0.01 |
| **Test result***: Negative*  *Positive* | *reference*  -0.42 | *---*  -0.61-(-)0.24 | ---  <0.01 | *reference*  -0.36 | ---  -0.54-(-)0.18 | ---  <0.01 |

Abbreviations: CI= confidence interval; DCT=digital contact tracing; Index=a person who tested SARS-CoV-2 positive; MCT=manual contact tracing; Self=testing at one’s own initiative.

1. Includes 3,646 exposure-test intervals in 3,646 participants between 14 December 2020- 6 February 2021. Only participants who reported a close contact were asked the date of last exposure and dates are missing (n=480) or not logical (before testing date or more than 14 days after testing, n=5). Additional missing values for symptoms (n=21), age (n=9), gender (n=14), and test location (n=9).
2. The tobit coefficient represents the change in exposure-test interval (in days) for each unit change of the dependent variable.
