# Supplementary material for: The epidemiological impact of digital and manual contact tracing on the SARS-CoV-2 epidemic in the Netherlands: Empirical evidence
Source: PLOS Digit Health. 2023 Dec 29;2(12):e0000396. doi: 10.1371/journal.pdig.0000396 (PMC10756539; doi:10.1371/journal.pdig.0000396)
Supplement: S9 Table — (DOCX) [file pdig.0000396.s016.docx]

## Table S9: Tobit regression model for the exposure-test intervals – second RDT study

|  | **Univariable analysis^1^ (n=3,172)** | | | **Multivariable analysis^1^ (n=3,172)** | | |
| --- | --- | --- | --- | --- | --- | --- |
|  | **Coefficient^2^** | **95% CI** | **p-value** | **Coefficient^2^** | **95% CI** | **p-value** |
| **Age in years***: 16-29*  *30-44*  *45-59*  *60+* | *reference*  -0.13  -0.19  0.20 | ---  -0.32-0.06  -0.39-0.01  -0.08-0.48 | ---  0.17  0.06  0.16 | *reference*  -0.24  -0.11  -0.21 | ---  -0.41-(-)0.06  -0.30-0.08  -0.49-0.08 | ---  <0.01  0.27  0.16 |
| **Gender:** *Female*  *Male* | *reference*  -0.13 | ---  -0.28-0.03 | ---  0.11 | *reference*  -0.12 | ---  -0.26-0.02 | ---  0.10 |
| **Testing region:** *Brabant*  *Rotterdam*  *Zwolle* | *reference*  -0.24  -0.43 | ---  -0.42-(-)0.05  -0.64-(-)0.23 | ---  0.01  <0.01 | *reference*  -0.23  -0.55 | ---  -0.41-(-)0.06  -0.75-(-)0.35 | ---  <0.01  <0.01 |
| **DCT:** *No*  *Yes* | *reference*  0.38 | ---  0.07-0.69 | ---  0.02 | *reference*  0.41 | ---  0.10-0.71 | ---  <0.01 |
| **MCT:** *No*  *Yes* | *reference*  0.20 | ---  -0.07-0.46 | ---  0.15 | *reference*  0.67 | ---  0.41 0.92 | ---  <0.01 |
| **Index:** *No*  *Yes* | *reference*  0.52 | ---  0.37-0.68 | ---  <0.01 | *reference*  0.40 | ---  0.16-0.64 | ---  <0.01 |
| **Housemate:** *No*  *Yes* | *reference*  -1.78 | ---  -1.95-(-)1.62 | ---  <0.01 | *reference*  -1.67 | ---  -1.88-(-)1.46 | ---  <0.01 |
| **Self:** *No*  *Yes* | *reference*  0.70 | ---  0.34-1.06 | ---  <0.01 | *reference*  0.64 | ---  0.22-1.05 | ---  <0.01 |
| **Unknown** *No*  **Contact:** *Yes* | *reference*  0.22 | ---  0.01-0.42 | ---  0.04 | *reference*  0.11 | ---  -0.20-0.41 | ---  0.49 |
| **Symptoms:** *No*  *Yes* | *reference*  -0.50 | ---  -0.66-(-)0.33 | ---  <0.01 | *reference*  -0.32 | ---  -0.48-(-)0.16 | ---  <0.01 |
| **Test result***: Negative*  *Positive* | *reference*  -1.02 | ---  -1.24-(-)0.79 | ---  <0.01 | *reference*  -0.52 | ---  -0.74-(-)0.29 | ---  <0.01 |
| **Vaccinated:**  *No*  *Yes* | *reference*  0.24 | ---  -0.01-0.49 | ---  0.06 | *reference*  0.19 | ---  -0.07-0.45 | ---  0.15 |
| **Previous** *No*  **infection:** *Yes* | *reference*  0.09 | ---  -0.17-0.36 | ---  0.49 | *reference*  0.02 | ---  -0.23-0.26 | ---  0.88 |

Abbreviations: CI= confidence interval; DCT=digital contact tracing; Index=a person who tested SARS-CoV-2 positive; MCT=manual contact tracing; Self=testing at one’s own initiative.

1. Includes 3,172 exposure-test intervals in 3,172 participants between 12 April- 14 June 2021. Only participants who reported a close contact were asked the date of last exposure and dates are missing (n=519) or not logical (before testing date or more than 14 days after testing, n=39). Additional missing values for symptoms (n=13), age (n= 6), gender (n=9), vaccination status (n=1), and prior infection (n= 9).
2. The tobit coefficient represents the change in exposure-test interval (in days) for each unit increase of the dependent variable.
