# Supplementary material for: The epidemiological impact of digital and manual contact tracing on the SARS-CoV-2 epidemic in the Netherlands: Empirical evidence
Source: PLOS Digit Health. 2023 Dec 29;2(12):e0000396. doi: 10.1371/journal.pdig.0000396 (PMC10756539; doi:10.1371/journal.pdig.0000396)
Supplement: S1 File — (DOCX) [file pdig.0000396.s017.docx]

R-Script and Output

The Epidemiological impact of (digital) contact tracing on the SARS-CoV-2 pandemic

Wianne W.A. Ter Haar

## Public Health Service Amsterdam

> #Public Health Service of Amsterdam data

> Sys.setlocale("LC_TIME", "en_GB")

[1] "en_GB"

> #install packages

> library(readxl)

> library(dplyr)

> library(tidyverse)

> library(ggplot2)

> library(SurvRegCensCov)

> library(survival)

> library(eha)

> #import dataset

>coronit_data <- read_excel("~/Desktop/CM for paper/Data/GGD_Amsterdam/Export_coronit_AMS.xlsx")

>coronit <- coronit_data

>#postcode <- read.csv("~/Desktop/CM for paper/Data/GGD_Amsterdam/pc6_buurt_wijk_map.csv")

>gem2021 <- read.csv("~/Desktop/CM for paper/Data/GGD_Amsterdam/cbs_buurt_wijk_map/gem2021.csv", sep=";")

>pc6hnr20210801_gwb <- read.csv("~/Desktop/CM for paper/Data/GGD_Amsterdam/cbs_buurt_wijk_map/pc6hnr20210801_gwb.csv", sep=";")

>postcode <- left_join(pc6hnr20210801_gwb, gem2021, by = c("Gemeente2021" = "Gemcode2021"))

>print(length(unique(coronit$hash_acht_pc)))

> monitoring <- read_excel("~/Desktop/CM for paper/Data/GGD_Amsterdam/monitoring_contacten.xlsx")

> export_contacts <- read_excel("~/Desktop/CM for paper/Data/GGD_Amsterdam/Export_contacts_core.xlsx")

> #make new variable for day of testing

> coronit$day_testing<- as.Date(coronit$datum_monsterafname, origin= "1900-01-01")

> #remove duplicate entries

> coronit<- coronit %>%

+ distinct(hash_acht_pc, day_testing, .keep_all=TRUE)

> #improve variables for interpretation

> coronit$symptoms<- ifelse(coronit$aantal_klachten==0, "no", "yes")

> coronit$symptoms[coronit$symptoms == "no"] <- "0"

> coronit$symptoms[coronit$symptoms == "yes"] <- "100"

> coronit$symptoms<- as.numeric(coronit$symptoms)

> coronit$bco_melding[coronit$bco_melding == "1"] <- "10"

> coronit$bco_melding<- as.numeric(coronit$bco_melding)

> coronit$leeftijd_jaren<- ifelse(coronit$leeftijd_jaren >102, NA,coronit$leeftijd_jaren)

> coronit$uitslag<- ifelse(coronit$uitslag >10, NA,coronit$uitslag)

> coronit$geslacht<- ifelse(coronit$geslacht <1, NA,coronit$geslacht)

> #make new variable for reason for testing

> coronit$reason_testing <- coronit$bco_melding+ coronit$coronamelder_melding + coronit$symptoms

> coronit$reason_testing[coronit$reason_testing == "0"] <- "Unknown"

> coronit$reason_testing[coronit$reason_testing == "1"] <- "CoronaMelder"

> coronit$reason_testing[coronit$reason_testing == "10"] <- "BCO"

> coronit$reason_testing[coronit$reason_testing == "11"] <- "CoronaMelder & BCO"

> coronit$reason_testing[coronit$reason_testing == "100"] <- "Symptoms"

> coronit$reason_testing[coronit$reason_testing == "101"] <- "CoronaMelder & Symptoms"

> coronit$reason_testing[coronit$reason_testing == "110"] <- "BCO & Symptoms"

> coronit$reason_testing[coronit$reason_testing == "111"] <- "All reasons"

> #group hierarcy reasons for testing

> coronit$comb_reason_testing <- coronit$bco_melding+ coronit$coronamelder_melding + coronit$symptoms

> coronit$comb_reason_testing[coronit$comb_reason_testing == "0"] <- "Unknown"

> coronit$comb_reason_testing[coronit$comb_reason_testing == "1"] <- "DCT"

> coronit$comb_reason_testing[coronit$comb_reason_testing == "10"] <- "MCT"

> coronit$comb_reason_testing[coronit$comb_reason_testing == "11"] <- "DCT"

> coronit$comb_reason_testing[coronit$comb_reason_testing == "100"] <- "Symptoms"

> coronit$comb_reason_testing[coronit$comb_reason_testing == "101"] <- "DCT"

> coronit$comb_reason_testing[coronit$comb_reason_testing == "110"] <- "MCT"

> coronit$comb_reason_testing[coronit$comb_reason_testing == "111"] <- "DCT"

> #graph of reason for testing over time

> coronit$ID<- seq.int(nrow(coronit))

> grouped_reason_testing<- coronit %>%

+ group_by(comb_reason_testing, day_testing) %>%

+ summarise(uniqueid= n_distinct(ID))

`summarise()` has grouped output by 'comb_reason_testing'. You can override using the `.groups` argument.

> grouped_reason_testing<- transform(grouped_reason_testing, percent= ave(uniqueid, day_testing, FUN= prop.table))

> grouped_reason_testing$percent <- grouped_reason_testing$percent * 100

> ggplot(data = grouped_reason_testing, aes(x= day_testing, y=percent, color=fct_relevel(comb_reason_testing, "DCT", "MCT","Symptoms","Unknown"))) +

+ geom_line(size=1)+

+ ylim(0,100)+

+ scale_x_date(date_breaks = "2 months", date_labels = "%d %b. %Y") +

+ labs(y= "Percentage of total tests", x= "Testing date", color= "Reason for testing")+

+ scale_color_manual(values = c("deeppink", "darkblue", "orange", "dimgray")) +

+ geom_vline(xintercept = as.numeric(as.Date("2020-10-10")), size=1, color= "black", linetype= 4) +

+ geom_vline(xintercept = as.numeric(as.Date("2020-12-01")), size=1, color= "black", linetype= 4) +

+ theme(panel.background = element_rect(fill = "white"),

+ panel.grid.major = element_line(color = "lightgray", linetype = "dashed"),

+ axis.line = element_line(color = "white", size = 2),

+ legend.key = element_rect(fill = "white", size = 10),

+ legend.background = element_rect(fill = "white"),

+ legend.text = element_text(size = 14, color = "black"),

+ legend.title = element_text(size = 14, face = "bold"),

+ axis.text.x = element_text(size = 12, color = "black"),

+ axis.text.y = element_text(size = 12, color = "black"),

+ axis.title.x = element_text(size = 14, face = "bold"),

+ axis.title.y = element_text(size = 14, face = "bold"))

> #select subset of data from the period after 1 December until 31 May

> subset<- coronit %>%

+ filter(day_testing > as.Date("2020-11-30"))

> #add municipality to postcode

> postcode$pc4 <- stringi::stri_sub(postcode$PC6, 1, 4)

> postcode$gemeentenaam <- postcode$Gemeentenaam2021

> postcode <- subset(postcode, select = c(pc4, gemeentenaam))

> postcode <- postcode %>%

+ distinct()

> postcode <- postcode %>%

+ filter(gemeentenaam == "Amsterdam" | gemeentenaam == "Aalsmeer" | gemeentenaam == "Amstelveen" | gemeentenaam == "Diemen" | gemeentenaam == "Ouder-Amstel" | gemeentenaam == "Uithoorn")

> subset$pc4 <- as.character(subset$pc4)

> subset <- left_join(subset, postcode, by= "pc4")

> #number of missings per variable

> sum(is.na(subset$comb_reason_testing))

[1] 0

> sum(is.na(subset$symptoms))

[1] 0

> sum(is.na(subset$uitslag))

[1] 3480

> sum(is.na(subset$leeftijd_jaren))

[1] 196

> sum(is.na(subset$geslacht))

[1] 1130

> sum(is.na(subset$gemeentenaam))

[1] 49047

> #Descriptives of reason for testing

> subset$geslacht[subset$geslacht == "1"] <- "Males"

> subset$geslacht[subset$geslacht == "2"] <- "Females"

> subset$geslacht<- as.factor(subset$geslacht)

> subset$uitslag[subset$uitslag == "0"] <- "Negative"

> subset$uitslag[subset$uitslag == "10"] <- "Positive"

> subset$uitslag<- as.factor(subset$uitslag)

> subset$leeftijd_jaren<- as.numeric(subset$leeftijd_jaren)

> summary_table <- subset %>%

+ group_by(comb_reason_testing) %>%

+ summarise(

+ cases = n(),

+ prc_case = scales::percent(cases/562159, accuracy = 0.01L),

+ symp = sum(symptoms >= 10, na.rm = T),

+ tot_symp =sum(symptoms>= 0, na.rm= T),

+ prc_symp = scales::percent(symp/ tot_symp, accuracy = 0.01L),

+ pos_test_result = sum(uitslag == "Positive", na.rm = T),

+ tot_test_result =sum(uitslag== "Positive" | uitslag == "Negative", na.rm= T),

+ pos_prc_test_restult = scales::percent(pos_test_result/ tot_test_result, accuracy = 0.01L),

+ age_med = median(leeftijd_jaren, na.rm = T),

+ age_IQR = round(IQR(leeftijd_jaren, na.rm=T), digits = 2),

+ p25 = quantile(leeftijd_jaren, probs = .25, na.rm = T),

+ p75 = quantile(leeftijd_jaren, probs = .75, na.rm = T),

+ age_max = max(leeftijd_jaren, na.rm = T),

+ age_min = min(leeftijd_jaren, na.rm = T),

+ fgender = sum(geslacht == "Females", na.rm = T),

+ tot_gender = sum(geslacht== "Females" | geslacht == "Males", na.rm =T),

+ prc_fgender = scales::percent(fgender/ tot_gender, accuracy = 0.01L),

+ tot_loca = sum(gemeentenaam == "Amsterdam" | gemeentenaam == "Aalsmeer" | gemeentenaam == "Amstelveen" | gemeentenaam == "Diemen" | gemeentenaam == "Ouder-Amstel" | gemeentenaam == "Uithoorn", na.rm = T ),

+ AMS = sum(gemeentenaam == "Amsterdam" , na.rm = T),

+ prc_AMS = scales::percent(AMS/ tot_loca, accuracy = 0.01L),

+ AAL = sum(gemeentenaam == "Aalsmeer" , na.rm = T),

+ prc_AAL = scales::percent(AAL/ tot_loca, accuracy = 0.01L),

+ AVEEN = sum(gemeentenaam == "Amstelveen" , na.rm = T),

+ prc_AVEEN = scales::percent(AVEEN/ tot_loca, accuracy = 0.01L),

+ DIE = sum(gemeentenaam == "Diemen" , na.rm = T),

+ prc_DIE = scales::percent(DIE/ tot_loca, accuracy = 0.01L),

+ OUD = sum(gemeentenaam == "Ouder-Amstel" , na.rm = T),

+ prc_OUD = scales::percent(OUD/ tot_loca, accuracy = 0.01L),

+ UIT = sum(gemeentenaam == "Uithoorn" , na.rm = T),

+ prc_UIT = scales::percent(UIT/ tot_loca, accuracy = 0.01L), )

> summary_table <- subset %>%

+ summarise(

+ cases = n(),

+ prc_case = scales::percent(cases/562159 , accuracy = 0.01L),

+ symp = sum(symptoms >= 10, na.rm = T),

+ tot_symp =sum(symptoms>= 0, na.rm= T),

+ prc_symp = scales::percent(symp/ tot_symp, accuracy = 0.01L),

+ pos_test_result = sum(uitslag == "Positive", na.rm = T),

+ tot_test_result =sum(uitslag== "Positive" | uitslag == "Negative", na.rm= T),

+ pos_prc_test_restult = scales::percent(pos_test_result/ tot_test_result, accuracy = 0.01L),

+ age_med = median(leeftijd_jaren, na.rm = T),

+ age_IQR = round(IQR(leeftijd_jaren, na.rm=T), digits = 2),

+ p25 = quantile(leeftijd_jaren, probs = .25, na.rm = T),

+ p75 = quantile(leeftijd_jaren, probs = .75, na.rm = T),

+ fgender = sum(geslacht == "Females", na.rm = T),

+ tot_gender = sum(geslacht== "Females" | geslacht == "Males", na.rm =T),

+ prc_fgender = scales::percent(fgender/ tot_gender, accuracy = 0.01L),

+ tot_loca = sum(gemeentenaam == "Amsterdam" | gemeentenaam == "Aalsmeer" | gemeentenaam == "Amstelveen" | gemeentenaam == "Diemen" | gemeentenaam == "Ouder-Amstel" | gemeentenaam == "Uithoorn", na.rm = T ),

+ AMS = sum(gemeentenaam == "Amsterdam" , na.rm = T),

+ prc_AMS = scales::percent(AMS/ tot_loca, accuracy = 0.01L),

+ AAL = sum(gemeentenaam == "Aalsmeer" , na.rm = T),

+ prc_AAL = scales::percent(AAL/ tot_loca, accuracy = 0.01L),

+ AVEEN = sum(gemeentenaam == "Amstelveen" , na.rm = T),

+ prc_AVEEN = scales::percent(AVEEN/ tot_loca, accuracy = 0.01L),

+ DIE = sum(gemeentenaam == "Diemen" , na.rm = T),

+ prc_DIE = scales::percent(DIE/ tot_loca, accuracy = 0.01L),

+ OUD = sum(gemeentenaam == "Ouder-Amstel" , na.rm = T),

+ prc_OUD = scales::percent(OUD/ tot_loca, accuracy = 0.01L),

+ UIT = sum(gemeentenaam == "Uithoorn" , na.rm = T),

+ prc_UIT = scales::percent(UIT/ tot_loca, accuracy = 0.01L), )

> #stat analyses

> chisq.test(subset$comb_reason_testing, subset$symptoms)

Pearson's Chi-squared test

data: subset$comb_reason_testing and subset$symptoms

X-squared = 523241, df = 3, p-value < 2.2e-16

> chisq.test(subset$comb_reason_testing, subset$uitslag)

Pearson's Chi-squared test

data: subset$comb_reason_testing and subset$uitslag

X-squared = 2844.5, df = 3, p-value < 2.2e-16

> kruskal.test(leeftijd_jaren ~ comb_reason_testing, data = subset)

Kruskal-Wallis rank sum test

data: leeftijd_jaren by comb_reason_testing

Kruskal-Wallis chi-squared = 3969.5, df = 3, p-value < 2.2e-16

> chisq.test(subset$comb_reason_testing, subset$geslacht)

Pearson's Chi-squared test

data: subset$comb_reason_testing and subset$geslacht

X-squared = 415.41, df = 3, p-value < 2.2e-16

> chisq.test(subset$comb_reason_testing, subset$gemeentenaam)

Pearson's Chi-squared test

data: subset$comb_reason_testing and subset$gemeentenaam

X-squared = 4337.2, df = 15, p-value < 2.2e-16

> #ad-hoc analyses, perform pairwise comparisons with Bonferroni correction

> sig<- .05

> sig_age <- pairwise.t.test(subset$leeftijd_jaren, subset$comb_reason_testing,

+ p.adj = "bonferroni", pool.sd = FALSE)

> print(sig_age$p.value < 0.05)

DCT MCT Symptoms

MCT TRUE NA NA

Symptoms TRUE TRUE NA

Unknown TRUE TRUE TRUE

> sig_gend<- table(subset$comb_reason_testing, subset$geslacht)

> sigadj<-sig/(nrow(sig_gend)*ncol(sig_gend))

> qnorm(sigadj/2)

[1] -2.734369

> sig_test<- table(subset$comb_reason_testing, subset$uitslag)

> chisq.test(sig_test, correct= FALSE)$stdres

Negative Positive

DCT 9.875205 -9.875205

MCT -49.250192 49.250192

Symptoms -1.598319 1.598319

Unknown 24.000825 -24.000825

> chisq.test(sig_gend, correct= FALSE)$stdres

Females Males

DCT -0.6793008 0.6793008

MCT -4.9670078 4.9670078

Symptoms 19.9919987 -19.9919987

Unknown -18.8616393 18.8616393

> sig_loca<- table(subset$comb_reason_testing, subset$gemeentenaam)

> chisq.test(sig_loca, correct= FALSE)$stdres

Aalsmeer Amstelveen Amsterdam Diemen Ouder-Amstel Uithoorn

DCT -1.7046495 -2.1255228 2.3905518 -1.2030633 0.7486086 0.3874792

MCT 17.0839339 5.9165648 -17.1526981 -0.4610914 6.0653072 8.5343076

Symptoms -35.4359048 -34.0317649 61.6074869 -17.0586802 -12.2609467 -24.8182439

Unknown 29.9290866 34.2286511 -58.2706800 18.9431213 9.9014616 22.2669474

> #clean monitoring and export_contacts datasets. Create new variable for exposure date. Have one degree of contact per individual

> monitoring<- monitoring%>%

+ rename(exp_start = bls_start_dt,

+ exp_end = bls_eind_dt,

+ date_onset_symptoms= "Date of Onset")

> monitoring <- monitoring %>%

+ mutate(date_onset_symptoms = lubridate::ymd(date_onset_symptoms))

> monitoring <- monitoring %>%

+ mutate(exp_start = lubridate:: ymd(exp_start))

> monitoring <- monitoring %>%

+ mutate(exp_end = lubridate:: ymd(exp_end))

> monitoring$exposure <- with(monitoring, as.Date(ifelse(is.na(exp_start), exp_end, exp_start), origin= "1970-01-01"))

> monitoring<- monitoring %>%

+ filter(exposure > as.Date("2020-11-16"))

> monitoring<- monitoring %>%

+ filter(exposure < as.Date("2021-04-01"))

> monitoring<- monitoring %>%

+ distinct(hash_acht_pc, exposure, .keep_all=TRUE)

> export_contacts$hash_acht_pc[export_contacts$hash_acht_pc == ""] <- NA

> export_contacts<- export_contacts %>%

+ drop_na(hash_acht_pc)

> export_contacts<- export_contacts %>%

+ group_by(hash_acht_pc) %>%

+ arrange("Degree of Contact") %>%

+ slice(1)

> #make new dataset with degree of contact and exposure date

> two_join<- left_join(monitoring, export_contacts, by= "hash_acht_pc")

> #select variable to be kept in the dataset

> two_join<- two_join %>%

+ select(contact_type, hash_acht_pc, date_onset_symptoms, exp_start, exp_end, exposure, "Degree of Contact", Gender)

> #merge part of coronIT data with HPZone data

> updata<- subset %>%

+ filter(day_testing < as.Date("2021-04-01"))

> combined<- left_join(two_join, updata, by= "hash_acht_pc")

> #remove if test took place before exposure

> combined$int_exp_test<- difftime(combined$day_testing, combined$exp_end, units = "days")

> combined$int_exp_test<- as.numeric(combined$int_exp_test)

> combined$int_exp_test<- combined$int_exp_test %>% replace_na(100)

> combined<- combined%>%

+ filter(int_exp_test > -1 | is.na(int_exp_test))

> #make one test moment per exposure

> combined<- combined %>%

+ group_by(hash_acht_pc, exp_end) %>%

+ arrange(int_exp_test) %>%

+ slice(1)

> #if interval between exposure and testing is more than 14 days then the variable for interval is changed to NA

> combined<- combined %>%

+ mutate(int_exp_test = replace(int_exp_test, int_exp_test>14, NA))

> summary(combined$int_exp_test)

Min. 1st Qu. Median Mean 3rd Qu. Max. NA's

0.000 2.000 5.000 3.906 5.000 14.000 4398

> #remove if missing for interval exposure and testing as then reason for testing is also missing

> combined<- combined %>%

+ drop_na(int_exp_test)

> #number of missings per variable #if interval between exposure and testing is more than 14 days then there is a variable for reason for testing but not for when this test took place. If no test took place then no variable for reason for testing and also not for interval between exposure and testing.

> sum(is.na(combined$reason_testing))

[1] 0

> sum(is.na(combined$comb_reason_testing))

[1] 0

> sum(is.na(combined$symptoms))

[1] 0

> sum(is.na(combined$int_exp_test))

[1] 0

> sum(is.na(combined$uitslag))

[1] 78

> sum(is.na(combined$leeftijd_jaren))

[1] 0

> sum(is.na(combined$geslacht))

[1] 47

> sum(is.na(combined$gemeentenaam))

[1] 1794

> #Descriptives of interval for reason for testing

> combined$symptoms[combined$symptoms== "0"]<- "No"

> combined$symptoms[combined$symptoms== "100"]<- "Yes"

> combined$symptoms<- as.factor(combined$symptoms)

> combined$agegrp<-cut(combined$leeftijd_jaren, c(-1,14,29,44,59,100),

+ labels=c("0-14", "15-29", "30-44", "45-59", "60+") )

> combined$"Degree of Contact"[combined$"Degree of Contact" == "Onbekend"] <- NA

> levels(combined$"Degree of Contact")

NULL

> combined$deg_con<- ifelse(combined$contact_type=="Case","Case", combined$"Degree of Contact")

> combined$deg_con<- as.factor(combined$deg_con)

> combined$status<- 1

> summary_table <- combined %>%

+ group_by(comb_reason_testing) %>%

+ summarise(

+ cases = n(),

+ prc_case = scales::percent(cases/20647, accuracy = 0.01L),

+ all = round(mean(int_exp_test, na.rm = T), digits=2),

+ all_sd = round(sd(int_exp_test, na.rm = T), digits=2),

+ con_case = round(mean(int_exp_test [deg_con== "Case"], na.rm = T), digits = 2),

+ con_case_sd = round(sd(int_exp_test [deg_con== "Case"], na.rm = T), digits = 2),

+ con_huis = round(mean(int_exp_test [deg_con== "1"], na.rm = T), digits = 2),

+ con_huis_sd = round(sd(int_exp_test [deg_con== "1"], na.rm = T), digits = 2),

+ con_cllng = round(mean(int_exp_test [deg_con== "2"], na.rm = T), digits = 2),

+ con_cllng_sd = round(sd(int_exp_test [deg_con== "2"], na.rm = T), digits = 2),

+ con_clsh = round(mean(int_exp_test [deg_con== "3"], na.rm = T), digits = 2),

+ con_clsh_sd = round(sd(int_exp_test [deg_con== "3"], na.rm = T), digits = 2),

+ con_oth = round(mean(int_exp_test [deg_con== "4"], na.rm = T), digits = 2),

+ con_oth_sd = round(sd(int_exp_test [deg_con== "4"], na.rm = T), digits = 2),

+ no_symp= round(mean(int_exp_test [symptoms== "No"], na.rm = T), digits = 2),

+ no_sd= round(sd(int_exp_test [symptoms== "No"], na.rm = T), digits = 2),

+ yes_symp= round(mean(int_exp_test [symptoms== "Yes"], na.rm = T), digits = 2),

+ yes_sd= round(sd(int_exp_test [symptoms== "Yes"], na.rm = T), digits = 2),

+ neg_test= round(mean(int_exp_test [uitslag == "Negative"], na.rm = T), digits = 2),

+ neg_sd= round(sd(int_exp_test [uitslag == "Negative"], na.rm = T), digits = 2),

+ pos_test= round(mean(int_exp_test [uitslag== "Positive"], na.rm = T), digits = 2),

+ pos_sd= round(sd(int_exp_test [uitslag== "Positive"], na.rm = T), digits = 2),

+ age_0 = round(mean(int_exp_test [agegrp == "0-14"], na.rm = T), digits = 2),

+ age_0_sd = round(sd(int_exp_test [agegrp == "0-14"], na.rm = T), digits = 2),

+ age_1 = round(mean(int_exp_test [agegrp == "15-29"], na.rm = T), digits = 2),

+ age_1_sd = round(sd(int_exp_test [agegrp == "15-29"], na.rm = T), digits = 2),

+ age_2 = round(mean(int_exp_test [agegrp == "30-44"], na.rm = T), digits = 2),

+ age_2_sd = round(sd(int_exp_test [agegrp == "30-44"], na.rm = T), digits = 2),

+ age_3 = round(mean(int_exp_test [agegrp == "45-59"], na.rm = T), digits = 2),

+ age_3_sd = round(sd(int_exp_test [agegrp == "45-59"], na.rm = T), digits = 2),

+ age_4 = round(mean(int_exp_test [agegrp == "60+"], na.rm = T), digits = 2),

+ age_4_sd = round(sd(int_exp_test [agegrp == "60+"], na.rm = T), digits = 2),

+ female = round(mean(int_exp_test [geslacht== "Females"], na.rm = T), digits = 2),

+ female_sd= round(sd(int_exp_test [geslacht== "Females"], na.rm = T), digits = 2),

+ male= round(mean(int_exp_test [geslacht == "Males"], na.rm = T), digits = 2),

+ male_sd= round(sd(int_exp_test [geslacht == "Males"], na.rm = T), digits = 2),

+ AMS = round(mean(int_exp_test [gemeentenaam== "Amsterdam"], na.rm = T), digits = 2),

+ AMS_sd = round(sd(int_exp_test [gemeentenaam== "Amsterdam"], na.rm = T), digits = 2),

+ AAL = round(mean(int_exp_test [gemeentenaam== "Aalsmeer"], na.rm = T), digits = 2),

+ AAL_sd = round(sd(int_exp_test [gemeentenaam== "Aalsmeer"], na.rm = T), digits = 2),

+ VEEN = round(mean(int_exp_test [gemeentenaam== "Amstelveen"], na.rm = T), digits = 2),

+ VEEN_sd = round(sd(int_exp_test [gemeentenaam== "Amstelveen"], na.rm = T), digits = 2),

+ DIE = round(mean(int_exp_test [gemeentenaam== "Diemen"], na.rm = T), digits = 2),

+ DIE_sd = round(sd(int_exp_test [gemeentenaam== "Diemen"], na.rm = T), digits = 2),

+ OUD = round(mean(int_exp_test [gemeentenaam== "Ouder-Amstel"], na.rm = T), digits = 2),

+ OUD_sd = round(sd(int_exp_test [gemeentenaam== "Ouder-Amstel"], na.rm = T), digits = 2),

+ UIT = round(mean(int_exp_test [gemeentenaam== "Uithoorn"], na.rm = T), digits = 2),

+ UIT_sd = round(sd(int_exp_test [gemeentenaam== "Uithoorn"], na.rm = T), digits = 2),)

> summary_table <- combined %>%

+ group_by(comb_reason_testing) %>%

+ summarise(

+ cases = n(),

+ prc_case = scales::percent(cases/20647 , accuracy = 0.01L),

+ EXP= sum(deg_con== "1"| deg_con== "2"| deg_con== "3"| deg_con== "4"| deg_con== "5" | deg_con== "Case", na.rm = T),

+ con_case = sum(deg_con== "Case", na.rm = T),

+ con_case_pc = scales::percent (con_case/EXP),

+ con_huis = sum(deg_con== "1", na.rm = T),

+ con_huis_pc = scales::percent (con_huis/EXP),

+ con_cllng = sum(deg_con== "2", na.rm = T),

+ con_cllng_pc = scales::percent (con_cllng/EXP),

+ con_clsh = sum(deg_con== "3", na.rm = T),

+ con_clsh_pc = scales::percent (con_clsh/EXP),

+ con_oth = sum(deg_con== "4", na.rm = T),

+ con_oth_pc = scales::percent (con_oth/EXP),

+ tot_symp =sum(symptoms== "Yes" | symptoms== "No", na.rm= T),

+ no_symp = sum(symptoms== "No", na.rm = T),

+ prc_no = scales::percent(no_symp/ tot_symp),

+ yes_symp = sum(symptoms== "Yes", na.rm = T),

+ prc_yes = scales::percent(yes_symp/ tot_symp),

+ tot_test_result =sum(uitslag== "Positive" | uitslag == "Negative", na.rm= T),

+ neg_test_result = sum(uitslag == "Negative", na.rm = T),

+ neg_prc_test_restult = scales::percent(neg_test_result/ tot_test_result,),

+ pos_test_result = sum(uitslag== "Positive", na.rm = T),

+ pos_prc_test_restult = scales::percent(pos_test_result/ tot_test_result,),

+ allage= sum(leeftijd_jaren>= 0, na.rm = T),

+ age_0 = sum(agegrp == "0-14", na.rm = T),

+ age_0_pc = scales::percent (age_0/allage),

+ age_1 = sum(agegrp == "15-29", na.rm = T),

+ age_1_pc = scales::percent (age_1/allage),

+ age_2 = sum(agegrp == "30-44", na.rm = T),

+ age_2_pc = scales::percent (age_2/allage),

+ age_3 = sum(agegrp == "45-59", na.rm = T),

+ age_3_pc = scales::percent (age_3/allage),

+ age_4 = sum(agegrp == "60+", na.rm = T),

+ age_4_pc = scales::percent (age_4/allage),

+ totgender= sum(geslacht== "Females" | geslacht == "Males", na.rm=T),

+ female =sum(geslacht== "Females", na.rm = T),

+ female_pc = scales::percent(female/ totgender),

+ male = sum(geslacht == "Males", na.rm=T),

+ male_pc= scales::percent(male/ totgender),

+ MUN = sum(gemeentenaam== "Amsterdam"| gemeentenaam== "Aalsmeer"| gemeentenaam== "Amstelveen"| gemeentenaam== "Diemen" | gemeentenaam== "Ouder-Amstel"| gemeentenaam== "Uithoorn", na.rm = T),

+ AMS =sum(gemeentenaam== "Amsterdam", na.rm = T),

+ AMS_pc = scales::percent (AMS/MUN),

+ AAL =sum(gemeentenaam== "Aalsmeer", na.rm = T),

+ AAl_pc = scales::percent (AAL/MUN),

+ VEEN =sum(gemeentenaam== "Amstelveen", na.rm = T),

+ VEEN_pc = scales::percent (VEEN/MUN),

+ DIE =sum(gemeentenaam== "Diemen", na.rm = T),

+ DIE_pc = scales::percent (DIE/MUN),

+ OUD =sum(gemeentenaam== "Ouder-Amstel", na.rm = T),

+ OUD_pc = scales::percent (OUD/MUN),

+ UIT =sum(gemeentenaam== "Uithoorn", na.rm = T),

+ UIT_pc = scales::percent (UIT/MUN), )

> #releveling data to set reference group

> combined$AMS_or_subburb<- combined$gemeentenaam

> combined$AMS_or_subburb<- recode(combined$AMS_or_subburb, Aalsmeer= "subburb")

> combined$AMS_or_subburb<- recode(combined$AMS_or_subburb, Amstelveen= "subburb")

> combined$AMS_or_subburb<- recode(combined$AMS_or_subburb, Diemen= "subburb" )

> combined$AMS_or_subburb<- recode(combined$AMS_or_subburb, 'Ouder-Amstel'= "subburb")

> combined$AMS_or_subburb<- recode(combined$AMS_or_subburb, Uithoorn= "subburb")

> #combined$AMS_or_subburb <- relevel(combined$AMS_or_subburb, ref = "Amsterdam")

> combined$comb_reason_testing<- as.factor(combined$comb_reason_testing)

> combined$comb_reason_testing <- relevel(combined$comb_reason_testing, ref = 2)

> combined$notif_CM<- ifelse(combined$coronamelder_melding==1, "Yes", "No")

> combined$notif_BCO<- ifelse(combined$bco_melding==10, "Yes", "No")

> combined$deg_con<- relevel(combined$deg_con, ref= 1)

> combined$symptoms<- relevel(combined$symptoms, ref= "No")

> combined$uitslag <- relevel(combined$uitslag, ref = "Negative")

> combined$agegrp <- relevel(combined$agegrp, ref = 2)

> combined$geslacht <- relevel(combined$geslacht, ref = "Females")

> combined$int_exp_test_plus1<- combined$int_exp_test + 1

> #univariate analysis

> occ<-survreg(Surv(int_exp_test_plus1, status)~ deg_con,

+ data= combined, dist='weibull')

> summary(occ)

Call:

survreg(formula = Surv(int_exp_test_plus1, status) ~ deg_con,

data = combined, dist = "weibull")

Value Std. Error z p

(Intercept) 1.58205 0.00702 225.28 < 2e-16

deg_conCategory 1: huishoudcontact 0.13389 0.00891 15.02 < 2e-16

deg_conCategory 2a: nauw contact, lang 0.20179 0.00931 21.68 < 2e-16

deg_conCategory 2b: nauw contact, kort 0.23718 0.03316 7.15 8.5e-13

deg_conCategory 3: overig contact 0.12749 0.07729 1.65 0.099

Log(scale) -0.67206 0.00554 -121.23 < 2e-16

Scale= 0.511

Weibull distribution

Loglik(model)= -47746.6 Loglik(intercept only)= -47988.9

Chisq= 484.58 on 4 degrees of freedom, p= 1.4e-103

Number of Newton-Raphson Iterations: 7

n=20578 (69 observations deleted due to missingness)

> ConvertWeibull(occ,conf.level = 0.95)

$vars

Estimate SE

lambda 0.04513616 0.001100994

gamma 1.95826079 0.010856228

deg_conCategory 1: huishoudcontact -0.26219755 0.017467038

deg_conCategory 2a: nauw contact, lang -0.39515194 0.018092920

deg_conCategory 2b: nauw contact, kort -0.46445186 0.064892304

deg_conCategory 3: overig contact -0.24965777 0.151348662

$HR

HR LB UB

deg_conCategory 1: huishoudcontact 0.7693590 0.7434660 0.7961539

deg_conCategory 2a: nauw contact, lang 0.6735777 0.6501102 0.6978923

deg_conCategory 2b: nauw contact, kort 0.6284795 0.5534198 0.7137195

deg_conCategory 3: overig contact 0.7790674 0.5790910 1.0481012

$ETR

ETR LB UB

deg_conCategory 1: huishoudcontact 1.143271 1.1234739 1.163416

deg_conCategory 2a: nauw contact, lang 1.223588 1.2014699 1.246112

deg_conCategory 2b: nauw contact, kort 1.267664 1.1878976 1.352786

deg_conCategory 3: overig contact 1.135973 0.9762826 1.321784

> occ<-survreg(Surv(int_exp_test_plus1, status)~ notif_CM,

+ data= combined, dist='weibull')

> summary(occ)

Call:

survreg(formula = Surv(int_exp_test_plus1, status) ~ notif_CM,

data = combined, dist = "weibull")

Value Std. Error z p

(Intercept) 1.70799 0.00376 454.55 <2e-16

notif_CMYes 0.05894 0.02987 1.97 0.049

Log(scale) -0.67115 0.00557 -120.46 <2e-16

Scale= 0.511

Weibull distribution

Loglik(model)= -48153.3 Loglik(intercept only)= -48155.3

Chisq= 4.04 on 1 degrees of freedom, p= 0.044

Number of Newton-Raphson Iterations: 7

n= 20647

> ConvertWeibull(occ,conf.level = 0.95)

$vars

Estimate SE

lambda 0.03537854 0.0007776773

gamma 1.95647784 0.0109010161

notif_CMYes -0.11530525 0.0584513453

$HR

HR LB UB

notif_CMYes 0.8910941 0.7946387 0.9992576

$ETR

ETR LB UB

notif_CMYes 1.060706 1.000383 1.124667

> occ<-survreg(Surv(int_exp_test_plus1, status)~ notif_BCO,

+ data= combined, dist='weibull')

> summary(occ)

Call:

survreg(formula = Surv(int_exp_test_plus1, status) ~ notif_BCO,

data = combined, dist = "weibull")

Value Std. Error z p

(Intercept) 1.66708 0.00452 369.1 <2e-16

notif_BCOYes 0.11976 0.00760 15.8 <2e-16

Log(scale) -0.67284 0.00555 -121.3 <2e-16

Scale= 0.51

Weibull distribution

Loglik(model)= -48027.3 Loglik(intercept only)= -48155.3

Chisq= 256.06 on 1 degrees of freedom, p= 1.2e-57

Number of Newton-Raphson Iterations: 7

n= 20647

> ConvertWeibull(occ,conf.level = 0.95)

$vars

Estimate SE

lambda 0.03811512 0.0008519364

gamma 1.95980133 0.0108725223

notif_BCOYes -0.23471058 0.0148548340

$HR

HR LB UB

notif_BCOYes 0.7907997 0.7681075 0.8141622

$ETR

ETR LB UB

notif_BCOYes 1.127229 1.110572 1.144136

> occ<-survreg(Surv(int_exp_test_plus1, status)~ symptoms,

+ data= combined, dist='weibull')

> summary(occ)

Call:

survreg(formula = Surv(int_exp_test_plus1, status) ~ symptoms,

data = combined, dist = "weibull")

Value Std. Error z p

(Intercept) 1.77544 0.00473 375.8 <2e-16

symptomsYes -0.17450 0.00724 -24.1 <2e-16

Log(scale) -0.67361 0.00551 -122.3 <2e-16

Scale= 0.51

Weibull distribution

Loglik(model)= -47867.7 Loglik(intercept only)= -48155.3

Chisq= 575.27 on 1 degrees of freedom, p= 4e-127

Number of Newton-Raphson Iterations: 7

n= 20647

> ConvertWeibull(occ,conf.level = 0.95)

$vars

Estimate SE

lambda 0.03074003 0.0006973109

gamma 1.96131376 0.0108061428

symptomsYes 0.34225853 0.0141069713

$HR

HR LB UB

symptomsYes 1.408124 1.369724 1.447601

$ETR

ETR LB UB

symptomsYes 0.8398729 0.8280428 0.851872

> occ<-survreg(Surv(int_exp_test_plus1, status)~ uitslag,

+ data= combined, dist='weibull')

> summary(occ)

Call:

survreg(formula = Surv(int_exp_test_plus1, status) ~ uitslag,

data = combined, dist = "weibull")

Value Std. Error z p

(Intercept) 1.73336 0.00421 412.1 <2e-16

uitslagPositive -0.11146 0.00837 -13.3 <2e-16

Log(scale) -0.67217 0.00557 -120.7 <2e-16

Scale= 0.511

Weibull distribution

Loglik(model)= -47884.9 Loglik(intercept only)= -47970.5

Chisq= 171.03 on 1 degrees of freedom, p= 4.4e-39

Number of Newton-Raphson Iterations: 7

n=20569 (78 observations deleted due to missingness)

> ConvertWeibull(occ,conf.level = 0.95)

$vars

Estimate SE

lambda 0.03354861 0.0007508925

gamma 1.95848397 0.0109047878

uitslagPositive 0.21829440 0.0163688654

$HR

HR LB UB

uitslagPositive 1.243953 1.204678 1.284509

$ETR

ETR LB UB

uitslagPositive 0.8945264 0.8799705 0.909323

> occ<-survreg(Surv(int_exp_test_plus1, status)~ agegrp,

+ data= combined, dist='weibull')

> summary(occ)

Call:

survreg(formula = Surv(int_exp_test_plus1, status) ~ agegrp,

data = combined, dist = "weibull")

Value Std. Error z p

(Intercept) 1.67049 0.00655 254.97 < 2e-16

agegrp0-14 0.09806 0.00996 9.85 < 2e-16

agegrp30-44 0.02110 0.01043 2.02 0.043

agegrp45-59 0.02541 0.01023 2.48 0.013

agegrp60+ 0.07570 0.01342 5.64 1.7e-08

Log(scale) -0.67327 0.00557 -120.93 < 2e-16

Scale= 0.51

Weibull distribution

Loglik(model)= -48097.3 Loglik(intercept only)= -48155.3

Chisq= 116.07 on 4 degrees of freedom, p= 3.7e-24

Number of Newton-Raphson Iterations: 7

n= 20647

> ConvertWeibull(occ,conf.level = 0.95)

$vars

Estimate SE

lambda 0.03780816 0.0009095313

gamma 1.96064088 0.0109158849

agegrp0-14 -0.19226997 0.0195419897

agegrp30-44 -0.04137655 0.0204567695

agegrp45-59 -0.04981824 0.0200651609

agegrp60+ -0.14841888 0.0263116083

$HR

HR LB UB

agegrp0-14 0.8250841 0.7940796 0.8572991

agegrp30-44 0.9594678 0.9217594 0.9987188

agegrp45-59 0.9514023 0.9147127 0.9895636

agegrp60+ 0.8620699 0.8187400 0.9076930

$ETR

ETR LB UB

agegrp0-14 1.103034 1.081710 1.124780

agegrp30-44 1.021328 1.000657 1.042425

agegrp45-59 1.025735 1.005370 1.046512

agegrp60+ 1.078638 1.050642 1.107380

> occ<-survreg(Surv(int_exp_test_plus1, status)~ geslacht,

+ data= combined, dist='weibull')

> summary(occ)

Call:

survreg(formula = Surv(int_exp_test_plus1, status) ~ geslacht,

data = combined, dist = "weibull")

Value Std. Error z p

(Intercept) 1.69775 0.00502 338.51 <2e-16

geslachtMales 0.02330 0.00713 3.27 0.0011

Log(scale) -0.67130 0.00558 -120.34 <2e-16

Scale= 0.511

Weibull distribution

Loglik(model)= -48038.4 Loglik(intercept only)= -48043.7

Chisq= 10.67 on 1 degrees of freedom, p= 0.0011

Number of Newton-Raphson Iterations: 7

n=20600 (47 observations deleted due to missingness)

> ConvertWeibull(occ,conf.level = 0.95)

$vars

Estimate SE

lambda 0.03607636 0.0008235593

gamma 1.95677104 0.0109150767

geslachtMales -0.04559608 0.0139640068

$HR

HR LB UB

geslachtMales 0.9554278 0.9296333 0.981938

$ETR

ETR LB UB

geslachtMales 1.023575 1.009362 1.037989

> occ<-survreg(Surv(int_exp_test_plus1, status)~ AMS_or_subburb,

+ data= combined, dist='weibull')

> summary(occ)

Call:

survreg(formula = Surv(int_exp_test_plus1, status) ~ AMS_or_subburb,

data = combined, dist = "weibull")

Value Std. Error z p

(Intercept) 1.71310 0.00434 394.30 <2e-16

AMS_or_subburbsubburb -0.01132 0.00907 -1.25 0.21

Log(scale) -0.67419 0.00584 -115.54 <2e-16

Scale= 0.51

Weibull distribution

Loglik(model)= -43972.5 Loglik(intercept only)= -43973.2

Chisq= 1.55 on 1 degrees of freedom, p= 0.21

Number of Newton-Raphson Iterations: 7

n=18853 (1794 observations deleted due to missingness)

> ConvertWeibull(occ,conf.level = 0.95)

$vars

Estimate SE

lambda 0.03467107 0.0008118411

gamma 1.96243608 0.0114511797

AMS_or_subburbsubburb 0.02220843 0.0178075803

$HR

HR LB UB

AMS_or_subburbsubburb 1.022457 0.9873864 1.058773

$ETR

ETR LB UB

AMS_or_subburbsubburb 0.988747 0.9713179 1.006489

> #multivariate analysis with Weibull distribution

> occ<-survreg(Surv(int_exp_test_plus1, status)~ deg_con + notif_CM + symptoms + agegrp + geslacht + AMS_or_subburb ,

+ data= combined, dist='weibull')

> summary(occ)

Call:

survreg(formula = Surv(int_exp_test_plus1, status) ~ deg_con +

notif_CM + symptoms + agegrp + geslacht + AMS_or_subburb,

data = combined, dist = "weibull")

Value Std. Error z p

(Intercept) 1.63295 0.01128 144.73 < 2e-16

deg_conCategory 1: huishoudcontact 0.09238 0.00960 9.63 < 2e-16

deg_conCategory 2a: nauw contact, lang 0.16567 0.00989 16.75 < 2e-16

deg_conCategory 2b: nauw contact, kort 0.21298 0.03471 6.14 8.4e-10

deg_conCategory 3: overig contact 0.09001 0.08056 1.12 0.2638

notif_CMYes 0.08311 0.03179 2.61 0.0089

symptomsYes -0.14756 0.00791 -18.65 < 2e-16

agegrp0-14 0.06439 0.01050 6.13 8.7e-10

agegrp30-44 0.01481 0.01095 1.35 0.1761

agegrp45-59 0.03168 0.01079 2.94 0.0033

agegrp60+ 0.06380 0.01400 4.56 5.2e-06

geslachtMales 0.01613 0.00745 2.17 0.0303

AMS_or_subburbsubburb -0.02196 0.00910 -2.41 0.0159

Log(scale) -0.67895 0.00576 -117.83 < 2e-16

Scale= 0.507

Weibull distribution

Loglik(model)= -43269.7 Loglik(intercept only)= -43720.4

Chisq= 901.35 on 12 degrees of freedom, p= 3e-185

Number of Newton-Raphson Iterations: 7

n=18749 (1898 observations deleted due to missingness)

> ConvertWeibull(occ,conf.level = 0.95)

$vars

Estimate SE

lambda 0.03996044 0.001218753

gamma 1.97180462 0.011361853

deg_conCategory 1: huishoudcontact -0.18214969 0.018938636

deg_conCategory 2a: nauw contact, lang -0.32666925 0.019406067

deg_conCategory 2b: nauw contact, kort -0.41996171 0.068403245

deg_conCategory 3: overig contact -0.17749115 0.158836030

notif_CMYes -0.16388639 0.062683729

symptomsYes 0.29096426 0.015530410

agegrp0-14 -0.12696919 0.020720090

agegrp30-44 -0.02919853 0.021586212

agegrp45-59 -0.06245938 0.021279470

agegrp60+ -0.12580126 0.027615633

geslachtMales -0.03181482 0.014686927

AMS_or_subburbsubburb 0.04329554 0.017950368

$HR

HR LB UB

deg_conCategory 1: huishoudcontact 0.8334766 0.8031059 0.8649958

deg_conCategory 2a: nauw contact, lang 0.7213223 0.6944019 0.7492863

deg_conCategory 2b: nauw contact, kort 0.6570720 0.5746296 0.7513424

deg_conCategory 3: overig contact 0.8373684 0.6133595 1.1431890

notif_CMYes 0.8488385 0.7507037 0.9598017

symptomsYes 1.3377168 1.2976114 1.3790616

agegrp0-14 0.8807608 0.8457091 0.9172653

agegrp30-44 0.9712236 0.9309900 1.0131960

agegrp45-59 0.9394512 0.9010754 0.9794615

agegrp60+ 0.8817901 0.8353313 0.9308328

geslachtMales 0.9686860 0.9411990 0.9969756

AMS_or_subburbsubburb 1.0442465 1.0081465 1.0816391

$ETR

ETR LB UB

deg_conCategory 1: huishoudcontact 1.0967784 1.0763426 1.1176022

deg_conCategory 2a: nauw contact, lang 1.1801838 1.1575214 1.2032899

deg_conCategory 2b: nauw contact, kort 1.2373641 1.1559924 1.3244638

deg_conCategory 3: overig contact 1.0941902 0.9343756 1.2813394

notif_CMYes 1.0866667 1.0210311 1.1565215

symptomsYes 0.8628086 0.8495284 0.8762964

agegrp0-14 1.0665108 1.0447856 1.0886877

agegrp30-44 1.0149182 0.9933756 1.0369279

agegrp45-59 1.0321833 1.0105850 1.0542432

agegrp60+ 1.0658793 1.0370271 1.0955342

geslachtMales 1.0162657 1.0015388 1.0312092

AMS_or_subburbsubburb 0.9782820 0.9609828 0.9958926

> #Weibull analysis among contacts

> contacts <- combined %>%

+ dplyr::filter(contact_type== "Contact")

> occ<-survreg(Surv(int_exp_test_plus1, status)~ deg_con + notif_CM + symptoms + agegrp + geslacht + AMS_or_subburb,

+ data= contacts, dist='weibull')

> summary(occ)

Call:

survreg(formula = Surv(int_exp_test_plus1, status) ~ deg_con +

notif_CM + symptoms + agegrp + geslacht + AMS_or_subburb,

data = contacts, dist = "weibull")

Value Std. Error z p

(Intercept) 1.03552 0.07261 14.26 < 2e-16

deg_conCategory 1: huishoudcontact 0.69837 0.07231 9.66 < 2e-16

deg_conCategory 2a: nauw contact, lang 0.75706 0.07233 10.47 < 2e-16

deg_conCategory 2b: nauw contact, kort 0.80229 0.07826 10.25 < 2e-16

deg_conCategory 3: overig contact 0.67674 0.00000 Inf < 2e-16

notif_CMYes 0.10042 0.03382 2.97 0.0030

symptomsYes -0.14495 0.00839 -17.28 < 2e-16

agegrp0-14 0.08356 0.01080 7.73 1.0e-14

agegrp30-44 0.05014 0.01148 4.37 1.3e-05

agegrp45-59 0.04524 0.01149 3.94 8.2e-05

agegrp60+ 0.07010 0.01539 4.56 5.2e-06

geslachtMales 0.02128 0.00784 2.71 0.0067

AMS_or_subburbsubburb -0.02301 0.00959 -2.40 0.0165

Log(scale) -0.78568 0.00673 -116.74 < 2e-16

Scale= 0.456

Weibull distribution

Loglik(model)= -31412.3 Loglik(intercept only)= -31643.7

Chisq= 462.73 on 12 degrees of freedom, p= 1.9e-91

Number of Newton-Raphson Iterations: 8

n=13672 (1379 observations deleted due to missingness)

> ConvertWeibull(occ,conf.level = 0.95)

$vars

Estimate SE

lambda 0.10312189 0.01651165

gamma 2.19390763 0.01476547

deg_conCategory 1: huishoudcontact -1.53216758 0.15912423

deg_conCategory 2a: nauw contact, lang -1.66091333 0.15907413

deg_conCategory 2b: nauw contact, kort -1.76014204 0.17209349

deg_conCategory 3: overig contact -1.48471453 0.00999245

notif_CMYes -0.22030452 0.07420524

symptomsYes 0.31800064 0.01826994

agegrp0-14 -0.18332358 0.02373352

agegrp30-44 -0.11000987 0.02520214

agegrp45-59 -0.09924323 0.02520566

agegrp60+ -0.15380026 0.03378113

geslachtMales -0.04667926 0.01721032

AMS_or_subburbsubburb 0.05047212 0.02104891

$HR

HR LB UB

deg_conCategory 1: huishoudcontact 0.2160668 0.1581763 0.2951446

deg_conCategory 2a: nauw contact, lang 0.1899654 0.1390818 0.2594649

deg_conCategory 2b: nauw contact, kort 0.1720204 0.1227704 0.2410273

deg_conCategory 3: overig contact 0.2265670 0.2221729 0.2310480

notif_CMYes 0.8022745 0.6936803 0.9278688

symptomsYes 1.3743771 1.3260336 1.4244832

agegrp0-14 0.8324987 0.7946604 0.8721388

agegrp30-44 0.8958253 0.8526508 0.9411859

agegrp45-59 0.9055224 0.8618747 0.9513806

agegrp60+ 0.8574433 0.8025107 0.9161360

geslachtMales 0.9543935 0.9227371 0.9871358

AMS_or_subburbsubburb 1.0517675 1.0092596 1.0960658

$ETR

ETR LB UB

deg_conCategory 1: huishoudcontact 2.010481 1.744818 2.3165923

deg_conCategory 2a: nauw contact, lang 2.131993 1.850201 2.4567027

deg_conCategory 2b: nauw contact, kort 2.230635 1.913425 2.6004321

deg_conCategory 3: overig contact 1.967462 1.967462 1.9674619

notif_CMYes 1.105631 1.034727 1.1813945

symptomsYes 0.865068 0.850966 0.8794037

agegrp0-14 1.087151 1.064370 1.1104187

agegrp30-44 1.051422 1.028026 1.0753502

agegrp45-59 1.046275 1.022983 1.0700964

agegrp60+ 1.072619 1.040748 1.1054661

geslachtMales 1.021505 1.005923 1.0373277

AMS_or_subburbsubburb 0.977257 0.959055 0.9958045

> #Weibull analysis among asymptomatic

> day_2 <- combined %>%

+ dplyr::filter(symptoms== "No")

> day_2$deg_con <- droplevels(day_2$deg_con)

> occ<-survreg(Surv(int_exp_test_plus1, status)~ deg_con + notif_CM + agegrp + geslacht + AMS_or_subburb,

+ data= day_2, dist='weibull')

> summary(occ)

Call:

survreg(formula = Surv(int_exp_test_plus1, status) ~ deg_con +

notif_CM + agegrp + geslacht + AMS_or_subburb, data = day_2,

dist = "weibull")

Value Std. Error z p

(Intercept) 1.66066 0.01218 136.39 < 2e-16

deg_conCategory 1: huishoudcontact 0.12537 0.01103 11.37 < 2e-16

deg_conCategory 2a: nauw contact, lang 0.13783 0.01130 12.19 < 2e-16

deg_conCategory 2b: nauw contact, kort 0.17593 0.03565 4.94 8.0e-07

deg_conCategory 3: overig contact 0.14071 0.08781 1.60 0.1091

notif_CMYes 0.11136 0.03555 3.13 0.0017

agegrp0-14 0.05977 0.01088 5.49 4.0e-08

agegrp30-44 0.02935 0.01235 2.38 0.0175

agegrp45-59 0.01301 0.01192 1.09 0.2749

agegrp60+ 0.06317 0.01496 4.22 2.4e-05

geslachtMales 0.01307 0.00796 1.64 0.1007

AMS_or_subburbsubburb -0.01413 0.00946 -1.49 0.1354

Log(scale) -0.87094 0.00751 -115.92 < 2e-16

Scale= 0.419

Weibull distribution

Loglik(model)= -25444.3 Loglik(intercept only)= -25550.4

Chisq= 212.16 on 11 degrees of freedom, p= 2.2e-39

Number of Newton-Raphson Iterations: 7

n=11134 (858 observations deleted due to missingness)

> ConvertWeibull(occ,conf.level = 0.95)

$vars

Estimate SE

lambda 0.01891920 0.0008463003

gamma 2.38916159 0.0179501006

deg_conCategory 1: huishoudcontact -0.29953625 0.0263877206

deg_conCategory 2a: nauw contact, lang -0.32930850 0.0268310879

deg_conCategory 2b: nauw contact, kort -0.42032620 0.0850963211

deg_conCategory 3: overig contact -0.33618262 0.2097691747

notif_CMYes -0.26605271 0.0849661803

agegrp0-14 -0.14279240 0.0260195086

agegrp30-44 -0.07012263 0.0295117996

agegrp45-59 -0.03108024 0.0284679029

agegrp60+ -0.15092484 0.0357682697

geslachtMales -0.03121917 0.0190238805

AMS_or_subburbsubburb 0.03375802 0.0226090458

$HR

HR LB UB

deg_conCategory 1: huishoudcontact 0.7411619 0.7038041 0.7805026

deg_conCategory 2a: nauw contact, lang 0.7194210 0.6825657 0.7582664

deg_conCategory 2b: nauw contact, kort 0.6568325 0.5559305 0.7760483

deg_conCategory 3: overig contact 0.7144926 0.4736329 1.0778382

notif_CMYes 0.7663987 0.6488308 0.9052700

agegrp0-14 0.8669340 0.8238311 0.9122921

agegrp30-44 0.9322795 0.8798844 0.9877945

agegrp45-59 0.9693978 0.9167905 1.0250238

agegrp60+ 0.8599123 0.8016931 0.9223594

geslachtMales 0.9692631 0.9337885 1.0060854

AMS_or_subburbsubburb 1.0343343 0.9895006 1.0811993

$ETR

ETR LB UB

deg_conCategory 1: huishoudcontact 1.1335711 1.1093287 1.158343

deg_conCategory 2a: nauw contact, lang 1.1477854 1.1226352 1.173499

deg_conCategory 2b: nauw contact, kort 1.1923551 1.1118899 1.278643

deg_conCategory 3: overig contact 1.1510926 0.9690912 1.367275

notif_CMYes 1.1177952 1.0425526 1.198468

agegrp0-14 1.0615889 1.0391866 1.084474

agegrp30-44 1.0297853 1.0051582 1.055016

agegrp45-59 1.0130938 0.9897084 1.037032

agegrp60+ 1.0652086 1.0344197 1.096914

geslachtMales 1.0131527 0.9974658 1.029086

AMS_or_subburbsubburb 0.9859697 0.9678533 1.004425

> sum(is.na(day_2$uitslag))

[1] 47

> sum(is.na(day_2$geslacht))

[1] 28

> #Linear model with tobit

> occ <- vglm(int_exp_test ~ deg_con,

+ family= tobit(Lower = 0, Upper = 14), data = combined)

> summary(occ)

Call:

vglm(formula = int_exp_test ~ deg_con, family = tobit(Lower = 0,

Upper = 14), data = combined)

Coefficients:

Estimate Std. Error z value Pr(>|z|)

(Intercept):1 2.780349 0.038530 72.160 < 2e-16 ***

(Intercept):2 1.040754 0.005336 195.029 < 2e-16 ***

deg_conCategory 1: huishoudcontact 0.826430 0.050062 16.508 < 2e-16 ***

deg_conCategory 2a: nauw contact, lang 1.838932 0.051802 35.499 < 2e-16 ***

deg_conCategory 2b: nauw contact, kort 2.120702 0.184362 11.503 < 2e-16 ***

deg_conCategory 3: overig contact 1.344170 0.430968 3.119 0.00181 **

---

Signif. codes: 0 ‘***’ 0.001 ‘**’ 0.01 ‘*’ 0.05 ‘.’ 0.1 ‘ ’ 1

Names of linear predictors: mu, loglink(sd)

Log-likelihood: -47117.17 on 41150 degrees of freedom

Number of Fisher scoring iterations: 5

No Hauck-Donner effect found in any of the estimates

> b <- coef(occ)

> se <- sqrt(diag(vcov(occ)))

> cbind(LL = b - qnorm(0.975) * se, UL = b + qnorm(0.975) * se)

LL UL

(Intercept):1 2.7048308 2.8558676

(Intercept):2 1.0302945 1.0512128

deg_conCategory 1: huishoudcontact 0.7283102 0.9245497

deg_conCategory 2a: nauw contact, lang 1.7374020 1.9404627

deg_conCategory 2b: nauw contact, kort 1.7593590 2.4820442

deg_conCategory 3: overig contact 0.4994874 2.1888526

> occ <- vglm(int_exp_test ~ notif_CM,

+ family= tobit(Lower = 0, Upper = 14), data = combined)

> summary(occ)

Call:

vglm(formula = int_exp_test ~ notif_CM, family = tobit(Lower = 0,

Upper = 14), data = combined)

Coefficients:

Estimate Std. Error z value Pr(>|z|)

(Intercept):1 3.726834 0.020673 180.273 <2e-16 ***

(Intercept):2 1.071492 0.005331 201.009 <2e-16 ***

notif_CMYes 0.362601 0.171760 2.111 0.0348 *

---

Signif. codes: 0 ‘***’ 0.001 ‘**’ 0.01 ‘*’ 0.05 ‘.’ 0.1 ‘ ’ 1

Names of linear predictors: mu, loglink(sd)

Log-likelihood: -47927.72 on 41291 degrees of freedom

Number of Fisher scoring iterations: 5

No Hauck-Donner effect found in any of the estimates

> b <- coef(occ)

> se <- sqrt(diag(vcov(occ)))

> cbind(LL = b - qnorm(0.975) * se, UL = b + qnorm(0.975) * se)

LL UL

(Intercept):1 3.68631461 3.7673525

(Intercept):2 1.06104429 1.0819397

notif_CMYes 0.02595826 0.6992442

> occ <- vglm(int_exp_test ~ symptoms,

+ family= tobit(Lower = 0, Upper = 14), data = combined)

> summary(occ)

Call:

vglm(formula = int_exp_test ~ symptoms, family = tobit(Lower = 0,

Upper = 14), data = combined)

Coefficients:

Estimate Std. Error z value Pr(>|z|)

(Intercept):1 4.315479 0.026029 165.79 <2e-16 ***

(Intercept):2 1.042420 0.005326 195.74 <2e-16 ***

symptomsYes -1.390079 0.040447 -34.37 <2e-16 ***

---

Signif. codes: 0 ‘***’ 0.001 ‘**’ 0.01 ‘*’ 0.05 ‘.’ 0.1 ‘ ’ 1

Names of linear predictors: mu, loglink(sd)

Log-likelihood: -47355.99 on 41291 degrees of freedom

Number of Fisher scoring iterations: 5

No Hauck-Donner effect found in any of the estimates

> b <- coef(occ)

> se <- sqrt(diag(vcov(occ)))

> cbind(LL = b - qnorm(0.975) * se, UL = b + qnorm(0.975) * se)

LL UL

(Intercept):1 4.264463 4.366496

(Intercept):2 1.031982 1.052859

symptomsYes -1.469354 -1.310804

> occ <- vglm(int_exp_test ~ uitslag,

+ family= tobit(Lower = 0, Upper = 14), data = combined)

> summary(occ)

Call:

vglm(formula = int_exp_test ~ uitslag, family = tobit(Lower = 0,

Upper = 14), data = combined)

Coefficients:

Estimate Std. Error z value Pr(>|z|)

(Intercept):1 3.94325 0.02331 169.15 <2e-16 ***

(Intercept):2 1.06312 0.00534 199.10 <2e-16 ***

uitslagPositive -0.89160 0.04799 -18.58 <2e-16 ***

---

Signif. codes: 0 ‘***’ 0.001 ‘**’ 0.01 ‘*’ 0.05 ‘.’ 0.1 ‘ ’ 1

Names of linear predictors: mu, loglink(sd)

Log-likelihood: -47575.59 on 41135 degrees of freedom

Number of Fisher scoring iterations: 5

No Hauck-Donner effect found in any of the estimates

> b <- coef(occ)

> se <- sqrt(diag(vcov(occ)))

> cbind(LL = b - qnorm(0.975) * se, UL = b + qnorm(0.975) * se)

LL UL

(Intercept):1 3.8975547 3.9889377

(Intercept):2 1.0526498 1.0735810

uitslagPositive -0.9856599 -0.7975296

> occ <- vglm(int_exp_test ~ agegrp,

+ family= tobit(Lower = 0, Upper = 14), data = combined)

> summary(occ)

Call:

vglm(formula = int_exp_test ~ agegrp, family = tobit(Lower = 0,

Upper = 14), data = combined)

Coefficients:

Estimate Std. Error z value Pr(>|z|)

(Intercept):1 3.553161 0.037173 95.585 < 2e-16 ***

(Intercept):2 1.067487 0.005330 200.293 < 2e-16 ***

agegrp0-14 0.597439 0.057221 10.441 < 2e-16 ***

agegrp30-44 0.005693 0.060063 0.095 0.924

agegrp45-59 0.030334 0.058899 0.515 0.607

agegrp60+ 0.453947 0.077074 5.890 3.87e-09 ***

---

Signif. codes: 0 ‘***’ 0.001 ‘**’ 0.01 ‘*’ 0.05 ‘.’ 0.1 ‘ ’ 1

Names of linear predictors: mu, loglink(sd)

Log-likelihood: -47852.45 on 41288 degrees of freedom

Number of Fisher scoring iterations: 5

No Hauck-Donner effect found in any of the estimates

> b <- coef(occ)

> se <- sqrt(diag(vcov(occ)))

> cbind(LL = b - qnorm(0.975) * se, UL = b + qnorm(0.975) * se)

LL UL

(Intercept):1 3.48030370 3.6260177

(Intercept):2 1.05704081 1.0779325

agegrp0-14 0.48528784 0.7095898

agegrp30-44 -0.11202835 0.1234138

agegrp45-59 -0.08510722 0.1457745

agegrp60+ 0.30288466 0.6050085

> occ <- vglm(int_exp_test ~ geslacht,

+ family= tobit(Lower = 0, Upper = 14), data = combined)

> summary(occ)

Call:

vglm(formula = int_exp_test ~ geslacht, family = tobit(Lower = 0,

Upper = 14), data = combined)

Coefficients:

Estimate Std. Error z value Pr(>|z|)

(Intercept):1 3.680625 0.028198 130.53 < 2e-16 ***

(Intercept):2 1.071363 0.005337 200.75 < 2e-16 ***

geslachtMales 0.108521 0.041108 2.64 0.00829 **

---

Signif. codes: 0 ‘***’ 0.001 ‘**’ 0.01 ‘*’ 0.05 ‘.’ 0.1 ‘ ’ 1

Names of linear predictors: mu, loglink(sd)

Log-likelihood: -47817.87 on 41197 degrees of freedom

Number of Fisher scoring iterations: 5

No Hauck-Donner effect found in any of the estimates

> b <- coef(occ)

> se <- sqrt(diag(vcov(occ)))

> cbind(LL = b - qnorm(0.975) * se, UL = b + qnorm(0.975) * se)

LL UL

(Intercept):1 3.62535809 3.7358910

(Intercept):2 1.06090342 1.0818229

geslachtMales 0.02795126 0.1890901

> occ <- vglm(int_exp_test ~ AMS_or_subburb,

+ family= tobit(Lower = 0, Upper = 14), data = combined)

> summary(occ)

Call:

vglm(formula = int_exp_test ~ AMS_or_subburb, family = tobit(Lower = 0,

Upper = 14), data = combined)

Coefficients:

Estimate Std. Error z value Pr(>|z|)

(Intercept):1 3.759425 0.024147 155.686 <2e-16 ***

(Intercept):2 1.069850 0.005574 191.934 <2e-16 ***

AMS_or_subburbsubburb -0.080476 0.052367 -1.537 0.124

---

Signif. codes: 0 ‘***’ 0.001 ‘**’ 0.01 ‘*’ 0.05 ‘.’ 0.1 ‘ ’ 1

Names of linear predictors: mu, loglink(sd)

Log-likelihood: -43752.45 on 37703 degrees of freedom

Number of Fisher scoring iterations: 5

No Hauck-Donner effect found in any of the estimates

> b <- coef(occ)

> se <- sqrt(diag(vcov(occ)))

> cbind(LL = b - qnorm(0.975) * se, UL = b + qnorm(0.975) * se)

LL UL

(Intercept):1 3.7120965 3.80675270

(Intercept):2 1.0589251 1.08077491

AMS_or_subburbsubburb -0.1831136 0.02216101

> occ <- vglm(int_exp_test ~ deg_con + notif_CM + symptoms + agegrp + geslacht + AMS_or_subburb,

+ family= tobit(Lower = 0, Upper = 14), data = combined)

> summary(occ)

Call:

vglm(formula = int_exp_test ~ deg_con + notif_CM + symptoms +

agegrp + geslacht + AMS_or_subburb, family = tobit(Lower = 0,

Upper = 14), data = combined)

Coefficients:

Estimate Std. Error z value Pr(>|z|)

(Intercept):1 3.322928 0.061818 53.753 < 2e-16 ***

(Intercept):2 1.013780 0.005581 181.651 < 2e-16 ***

deg_conCategory 1: huishoudcontact 0.550950 0.052340 10.526 < 2e-16 ***

deg_conCategory 2a: nauw contact, lang 1.557300 0.054068 28.803 < 2e-16 ***

deg_conCategory 2b: nauw contact, kort 1.900519 0.189126 10.049 < 2e-16 ***

deg_conCategory 3: overig contact 1.201141 0.440122 2.729 0.00635 **

notif_CMYes 0.499779 0.173971 2.873 0.00407 **

symptomsYes -1.167111 0.043063 -27.103 < 2e-16 ***

agegrp0-14 0.319117 0.057590 5.541 3.01e-08 ***

agegrp30-44 0.016243 0.060058 0.270 0.78682

agegrp45-59 0.136626 0.059330 2.303 0.02129 *

agegrp60+ 0.339410 0.076830 4.418 9.98e-06 ***

geslachtMales 0.066578 0.040818 1.631 0.10287

AMS_or_subburbsubburb -0.130700 0.049954 -2.616 0.00889 **

---

Signif. codes: 0 ‘***’ 0.001 ‘**’ 0.01 ‘*’ 0.05 ‘.’ 0.1 ‘ ’ 1

Names of linear predictors: mu, loglink(sd)

Log-likelihood: -42478.7 on 37484 degrees of freedom

Number of Fisher scoring iterations: 5

No Hauck-Donner effect found in any of the estimates

> b <- coef(occ)

> se <- sqrt(diag(vcov(occ)))

> cbind(LL = b - qnorm(0.975) * se, UL = b + qnorm(0.975) * se)

LL UL

(Intercept):1 3.20176730 3.4440894

(Intercept):2 1.00284185 1.0247186

deg_conCategory 1: huishoudcontact 0.44836550 0.6535353

deg_conCategory 2a: nauw contact, lang 1.45132848 1.6632720

deg_conCategory 2b: nauw contact, kort 1.52983912 2.2711982

deg_conCategory 3: overig contact 0.33851724 2.0637653

notif_CMYes 0.15880130 0.8407565

symptomsYes -1.25151260 -1.0827094

agegrp0-14 0.20624149 0.4319916

agegrp30-44 -0.10146880 0.1339538

agegrp45-59 0.02034215 0.2529098

agegrp60+ 0.18882682 0.4899941

geslachtMales -0.01342468 0.1465808

AMS_or_subburbsubburb -0.22860881 -0.0327919

## First rapid diagnostic test accuracy study

> #First RDT evaluation study data

> Sys.setlocale("LC_TIME", "en_GB")

[1] "en_GB"

> #install packages

> library(readxl)

> library(dplyr)

> library(survival)

> library(SurvRegCensCov)

> library(eha)

> library(VGAM)

> #import the datasets and make that that all the dates are in datatype 'date' and age is 'numeric'

> Roche <- read_excel("~/Desktop/CM for paper/Data/First_antigentestround/export Roche_BMJ.xlsx")

> Veritor <- read_excel("~/Desktop/CM for paper/Data/First_antigentestround/export BD-veritor_BMJ.xlsx")

> #create a subset of the variables used

> Roche<- Roche %>%

+ select(Antigeen_sneltest, date_afname, last_contact, Uitslag_PCR, Ct, W_GGD, W_app, W_pers, W_ini, Klachten_Nee, Klachten_Ja,

+ Klachten, Afnamelocatie, Geslacht, Leeftijd)

> Veritor <- Veritor %>%

+ select(Antigeen_sneltest, date_afname, last_contact, Uitslag_PCR, Ct, W_GGD, W_app, W_pers, W_ini, Klachten_Nee, Klachten_Ja,

+ Klachten, Afnamelocatie, Geslacht, Leeftijd)

> #bind both datasets into one. For new variable "origns" 1=Veritor, 2= Roche

> new<- bind_rows(Roche, Veritor, .id= "origins")

> #change datatypes of some variables

> numeric_cols <- c("date_afname", "last_contact", "Leeftijd", "Klachten_Nee", "Klachten_Ja", "Klachten")

> new <- new %>%

+ mutate(across(all_of(numeric_cols), as.numeric))

> #convert variables to correct classification

> new<- new%>%

+ mutate(date_afname= as.Date(date_afname, origin= "1970-01-01"))

> new<- new%>%

+ mutate(last_contact= as.Date(last_contact, origin= "1970-01-01"))

> #recode variables for improved interpretation (0,1 are Rotterdam and 2,3 are Brabant)

> new$agegrp<-cut(new$Leeftijd, c(14,29,44,59,101),

+ labels=c("16-29", "30-44", "45-59", "60+") )

> new$Afnamelocatie[new$Afnamelocatie == "ZH D X L Rotterdam - Airportplein P3"] <- "0"

> new$Afnamelocatie[new$Afnamelocatie == "ZH D X Rotterdam - Airportplein P3"] <- "0"

> new$Afnamelocatie[new$Afnamelocatie == "ZH D Rotterdam - Koperstraat 23"] <- "0"

> new$Afnamelocatie[new$Afnamelocatie == "ZH D L Rotterdam - van Zandvlietplein 20"] <- "0"

> new$Afnamelocatie[new$Afnamelocatie == "ZH D Delft - Bieslandsekade 68"] <- "0"

> new$Afnamelocatie[new$Afnamelocatie == "ZH D X Rotterdam - Ahoy Rotterdam P1"] <- "1"

> new$Afnamelocatie[new$Afnamelocatie == "424"] <- "2"

> new$Afnamelocatie[new$Afnamelocatie == "423"] <- "2"

> new$Afnamelocatie[new$Afnamelocatie == "422"] <- "2"

> new$Afnamelocatie[new$Afnamelocatie == "137"] <- "3"

> new$Afnamelocatie[new$Afnamelocatie == "NA"] <- NA

> new$Afnamelocatie<- as.numeric(new$Afnamelocatie)

> new$locationRB<- new$Afnamelocatie

> new$locationRB[new$locationRB == "0"] <- "Rotterdam"

> new$locationRB[new$locationRB == "1"] <- "Rotterdam"

> new$locationRB[new$locationRB == "2"] <- "Brabant"

> new$locationRB[new$locationRB == "3"] <- "Brabant"

> new$Uitslag_PCR[new$Uitslag_PCR == "0"] <- "Negative"

> new$Uitslag_PCR[new$Uitslag_PCR == "1"] <- "Positive"

> print(table(new$Uitslag_PCR))

Negative Positive

3909 365

> new$Geslacht[new$Geslacht == "NA"] <- NA

> print(table(new$Geslacht))

Female Male

2121 2137

> #make variable for symptoms based on questionnaire

> new$Klachten_Nee[is.na(new$Klachten_Nee)] <- 0

> new$Klachten_Ja[is.na(new$Klachten_Ja)] <- 0

> new$symptoms_quest <- ifelse((new$Klachten_Nee == "1" & new$Klachten_Ja == "0"), 0, +

+ ifelse((new$Klachten_Nee == "0" & new$Klachten_Ja == "1"), 1, 3))

> new$symptoms_quest<- as.character(new$symptoms_quest)

> new$symptoms_quest[new$symptoms_quest == "0"] <- "No"

> new$symptoms_quest[new$symptoms_quest == "1"] <- "Yes"

> new<- new %>%

+ mutate(symptoms_quest = na_if(symptoms_quest, "3"))

> print(table(new$symptoms_quest))

No Yes

3729 377

> #make new variable for the interval between exposure and getting tested

> new$int_exp_test<- difftime(new$date_afname, new$last_contact, units = "days")

> new$int_exp_test<- as.numeric(new$int_exp_test)

> #Make new variable for method exposure notification

> new$W_GGD <- ifelse((new$W_GGD == "1"), 1, 0)

> new$W_app <- ifelse((new$W_app == "1"), 10, 0)

> new$W_pers <- ifelse((new$W_pers == "1"), 100, 0)

> new$W_ini <- ifelse((new$W_ini == "1"), 1000, 0)

> new$method_notification<- new$W_GGD + new$W_app + new$W_pers + new$W_ini

> new$method_notification<- as.character(new$method_notification)

> print(table(new$method_notification))

0 1 10 100 1000 1001 101 1010 11 110 1100 1101 111 1111

586 307 228 2388 479 7 189 3 4 38 21 4 19 1

> new$method_notification[new$method_notification == "0"] <- "Unknown"

> new$method_notification[new$method_notification == "1"] <- "BCO"

> new$method_notification[new$method_notification == "10"] <- "CM"

> new$method_notification[new$method_notification == "11"] <- "BCO & CM"

> new$method_notification[new$method_notification == "100"] <- "Index"

> new$method_notification[new$method_notification == "101"] <- "BCO & Index"

> new$method_notification[new$method_notification == "110"] <- "CM & Index"

> new$method_notification[new$method_notification == "111"] <- "BCO & CM & Index"

> new$method_notification[new$method_notification == "1000"] <- "Self"

> new$method_notification[new$method_notification == "1001"] <- "BCO & Self"

> new$method_notification[new$method_notification == "1010"] <- "CM & Self"

> new$method_notification[new$method_notification == "1011"] <- "BCO & CM & Self"

> new$method_notification[new$method_notification == "1100"] <- "Index & Self"

> new$method_notification[new$method_notification == "1101"] <- "BCO & Index & Self"

> new$method_notification[new$method_notification == "1110"] <- "CM & Index & Self"

> new$method_notification[new$method_notification == "1111"] <- "All methods"

> #combine the multiple ways of getting tested

> new$method_notification_multi<- new$W_GGD + new$W_app + new$W_pers + new$W_ini

> new$method_notification_multi<- as.character(new$method_notification_multi)

> new$method_notification_multi[new$method_notification_multi == "0"] <- NA

> #remove participants with blank questionnaire

> new <- new[!(is.na(new$symptoms_quest)) | !(is.na(new$last_contact)) | !(is.na(new$method_notification_multi)),]

> #if interval less than 0 or more than 14 then set to NA

> new<- new %>%

+ mutate(int_exp_test = replace(int_exp_test, int_exp_test< -1, NA))

> new<- new %>%

+ mutate(int_exp_test = replace(int_exp_test, int_exp_test>14, NA))

> #combine the multiple ways of getting tested

> new$method_notification_multi<- new$W_GGD + new$W_app + new$W_pers + new$W_ini

> new$method_notification_multi<- as.character(new$method_notification_multi)

> new$method_notification_multi[new$method_notification_multi == "0"] <- "Unknown"

> new$method_notification_multi[new$method_notification_multi == "1"] <- "MCT"

> new$method_notification_multi[new$method_notification_multi == "10"] <- "DCT"

> new$method_notification_multi[new$method_notification_multi == "11"] <- "DCT"

> new$method_notification_multi[new$method_notification_multi == "100"] <- "Index"

> new$method_notification_multi[new$method_notification_multi == "101"] <- "MCT"

> new$method_notification_multi[new$method_notification_multi == "110"] <- "DCT"

> new$method_notification_multi[new$method_notification_multi == "111"] <- "DCT"

> new$method_notification_multi[new$method_notification_multi == "1000"] <- "Self"

> new$method_notification_multi[new$method_notification_multi == "1001"] <- "MCT"

> new$method_notification_multi[new$method_notification_multi == "1010"] <- "DCT"

> new$method_notification_multi[new$method_notification_multi == "1011"] <- "DCT"

> new$method_notification_multi[new$method_notification_multi == "1100"] <- "Index"

> new$method_notification_multi[new$method_notification_multi == "1101"] <- "MCT"

> new$method_notification_multi[new$method_notification_multi == "1110"] <- "DCT"

> new$method_notification_multi[new$method_notification_multi == "1111"] <- "DCT"

> #reason for testing over time

> new$ID<- seq.int(nrow(new)

> grouped_reason_testing<- new %>%

+ group_by(method_notification_multi, date_afname) %>%

+ summarise(uniqueid= n_distinct(ID))

`summarise()` has grouped output by 'method_notification_multi'. You can override using the

`.groups` argument.

> grouped_reason_testing<- transform(grouped_reason_testing, percent= ave(uniqueid, date_afname, FUN= prop.table))

> grouped_reason_testing$percent <- grouped_reason_testing$percent * 100

> ggplot(data = grouped_reason_testing, aes(x = date_afname, y = percent, color = fct_relevel(method_notification_multi, "DCT", "MCT", "Index", "Self", "Unknown"))) +

+ geom_line(size=1) +

+ ylim(0,100)+

+ scale_x_date(date_breaks= "2 weeks", date_labels = "%d %b. %Y") +

+ scale_color_manual(values = c("deeppink3", "darkblue", "darkgreen", "yellow", "dimgrey")) +

+ labs(y="Percentage of total tests", x= "Testing date", color="Reason for testing") +

+ theme(panel.background = element_rect(fill = "white"),

+ panel.grid.major = element_line(color = "lightgray", linetype = "dashed"),

+ axis.line = element_line(color = "white", size = 2),

+ legend.key = element_rect(fill = "white", size = 10),

+ legend.background = element_rect(fill = "white"),

+ legend.text = element_text(size = 14, color = "black"),

+ legend.title = element_text(size = 14, face = "bold"),

+ axis.text.x = element_text(size = 12, color = "black"),

+ axis.text.y = element_text(size = 12, color = "black"),

+ axis.title.x = element_text(size = 14, face = "bold"),

+ axis.title.y = element_text(size = 14, face = "bold"))

> #for descriptive stats table in report

> summary_table <- new %>%

+ group_by(method_notification_multi) %>%

+ summarise(

+ cases = n(),

+ prc_case = scales::percent(cases/ 4131, accuracy = 0.01L),

+ tot_symp = sum(symptoms_quest == "No" | symptoms_quest == "Yes", na.rm = T),

+ symptoms_yes = sum(symptoms_quest == "Yes", na.rm = T),

+ prc_symptoms = scales::percent(symptoms_yes/ tot_symp, accuracy = 0.01L),

+ tot_test_result =sum(Uitslag_PCR== "Positive" | Uitslag_PCR== "Negative" , na.rm= T),

+ pos_test_result = sum(Uitslag_PCR == "Positive", na.rm = T),

+ pos_prc_test_result = scales::percent(pos_test_result/ tot_test_result, accuracy = 0.01L),

+ age_med = median(Leeftijd, na.rm = T),

+ age_IQR1 = quantile(Leeftijd, probs = c(.25), na.rm = T),

+ age_IQR2 = quantile(Leeftijd, probs = c(.75), na.rm = T),

+ age_min = min(Leeftijd, na.rm = T),

+ age_max = max (Leeftijd, na.rm= T),

+ tot_gender = sum(Geslacht == "Female" | Geslacht == "Male", na.rm =T),

+ fgender = sum(Geslacht == "Female", na.rm = T),

+ prc_fgender = scales::percent(fgender/ tot_gender, accuracy = 0.01L),

+ tot_loca =sum(locationRB== "Brabant" | locationRB== "Rotterdam", na.rm = T),

+ R_loca = sum(locationRB== "Brabant", na.rm = T),

+ prc_R_loca = scales::percent(R_loca/ tot_loca, accuracy = 0.01L),

+ B_loca = sum(locationRB== "Rotterdam" , na.rm = T),

+ prc_B1_loca = scales::percent(B_loca/ tot_loca, accuracy = 0.01L), )

> subset_new<- new %>%

+ filter(Uitslag_PCR =="Positive")

> summary_table <- subset_new %>%

+ group_by(method_notification_multi) %>%

+ summarise(

+ cases = n(),

+ Ct_med = median(Ct, na.rm = T),

+ Ct_IQR1 = quantile(Ct, probs = c(.25), na.rm = T),

+ Ct_IQR2 = quantile(Ct, probs = c(.75), na.rm = T),

+ all = sum(Ct>0),

+ low = sum(Ct<= 30),

+ low_pc = scales::percent(low/all, accuracy = 0.01L),

+ high =sum(Ct> 30),

+ high_pc = scales:: percent(high/all, accuracy = 0.01L))

> #Checking number of missing values

> sum(is.na(new$symptoms_quest))

[1] 25

> sum(is.na(new$Leeftijd))

[1] 10

> sum(is.na(new$Geslacht))

[1] 16

> sum(is.na(new$locationRB))

[1] 10

> #calculate p-values

> age_krus <- kruskal.test(Leeftijd ~ method_notification_multi, data = new)

> age.aov <- aov(Leeftijd ~ method_notification_multi, data = new)

> summary(age.aov)

Df Sum Sq Mean Sq F value Pr(>F)

method_notification_multi 4 12213 3053.2 10.41 2.2e-08 ***

Residuals 4116 1207702 293.4

---

Signif. codes: 0 ‘***’ 0.001 ‘**’ 0.01 ‘*’ 0.05 ‘.’ 0.1 ‘ ’ 1

10 observations deleted due to missingness

> chisq.test(new$method_notification_multi, new$Geslacht)

Pearson's Chi-squared test

data: new$method_notification_multi and new$Geslacht

X-squared = 6.1805, df = 4, p-value = 0.1861

> chisq.test(new$method_notification_multi, new$locationRB)

Pearson's Chi-squared test

data: new$method_notification_multi and new$locationRB

X-squared = 165.59, df = 4, p-value < 2.2e-16

> chisq.test(new$method_notification_multi, new$symptoms_quest)

Pearson's Chi-squared test

data: new$method_notification_multi and new$symptoms_quest

X-squared = 54.305, df = 4, p-value = 4.543e-11

> chisq.test(new$method_notification_multi, new$Uitslag_PCR)

Pearson's Chi-squared test

data: new$method_notification_multi and new$Uitslag_PCR

X-squared = 15.651, df = 4, p-value = 0.003525

> ct.aov <- aov(Ct ~ method_notification_multi, data = subset_new)

> kruskal.test(Ct~ method_notification_multi, data =new)

Kruskal-Wallis rank sum test

data: Ct by method_notification_multi

Kruskal-Wallis chi-squared = 21.751, df = 4, p-value = 0.0002247

> summary(ct.aov)

Df Sum Sq Mean Sq F value Pr(>F)

method_notification_multi 4 130 32.58 0.918 0.454

Residuals 344 12215 35.51

> #checking assumptions of one-way ANNOVA

> TukeyHSD(ct.aov)

Tukey multiple comparisons of means

95% family-wise confidence level

Fit: aov(formula = Ct ~ method_notification_multi, data = subset_new)

$method_notification_multi

diff lwr upr p adj

Index-DCT 1.0930000 -4.204626 6.390626 0.9798860

MCT-DCT 0.7796000 -4.881050 6.440250 0.9956639

Self-DCT 0.4247925 -5.209094 6.058678 0.9995918

Unknown-DCT 2.5915000 -3.185877 8.368877 0.7338004

MCT-Index -0.3134000 -2.902389 2.275589 0.9973725

Self-Index -0.6682075 -3.198143 1.861728 0.9507973

Unknown-Index 1.4985000 -1.336635 4.333635 0.5960871

Self-MCT -0.3548075 -3.576406 2.866791 0.9981836

Unknown-MCT 1.8119000 -1.654526 5.278326 0.6064940

Unknown-Self 2.1667075 -1.255838 5.589253 0.4132070

> TukeyHSD(age.aov)

Tukey multiple comparisons of means

95% family-wise confidence level

Fit: aov(formula = Leeftijd ~ method_notification_multi, data = new)

$method_notification_multi

diff lwr upr p adj

Index-DCT -5.8956720 -8.7927765 -2.9985674 0.0000003

MCT-DCT -4.7064994 -8.1406654 -1.2723334 0.0017457

Self-DCT -2.8790026 -6.3524110 0.5944058 0.1575922

Unknown-DCT -6.3438341 -9.8674915 -2.8201766 0.0000092

MCT-Index 1.1891726 -1.0955037 3.4738489 0.6144498

Self-Index 3.0166694 0.6734204 5.3599184 0.0040867

Unknown-Index -0.4481621 -2.8652702 1.9689460 0.9868020

Self-MCT 1.8274968 -1.1542975 4.8092911 0.4511516

Unknown-MCT -1.6373347 -4.6775144 1.4028451 0.5823058

Unknown-Self -3.4648315 -6.5492703 -0.3803926 0.0185913

> plot(age.aov, 1)

> plot(age.aov, 2)

> leveneTest(Leeftijd ~ method_notification_multi, data = new)

Levene's Test for Homogeneity of Variance (center = median)

Df F value Pr(>F)

group 4 2.0769 0.08115 .

4116

---

Signif. codes: 0 ‘***’ 0.001 ‘**’ 0.01 ‘*’ 0.05 ‘.’ 0.1 ‘ ’ 1

> #ad-hoc analyses, perform pairwise comparisons with Bonferroni correction

> sig<- .05

> sig_age <- pairwise.t.test(new$Leeftijd, new$method_notification_multi,

+ p.adj = "bonferroni", pool.sd = FALSE)

> print(sig_age$p.value < 0.05)

DCT Index MCT Self

Index TRUE NA NA NA

MCT TRUE FALSE NA NA

Self FALSE TRUE FALSE NA

Unknown TRUE FALSE FALSE TRUE

> sig_location<- table(new$method_notification_multi, new$locationRB)

> sigadj_loca<-sig/(nrow(sig_location)*ncol(sig_location))

> qnorm(sigadj_loca/2)

[1] -2.807034

> chisq.test(sig_location, correct= FALSE)$stdres

Brabant Rotterdam

DCT 3.223738 -3.223738

Index -12.753427 12.753427

MCT 4.794063 -4.794063

Self 6.676325 -6.676325

Unknown 5.652201 -5.652201

> sig_symp<- table(new$method_notification_multi, new$symptoms_quest)

> sigadj_sym<-sig/(nrow(sig_symp)*ncol(sig_symp))

> qnorm(sigadj_sym/2)

[1] -2.807034

> chisq.test(sig_symp, correct= FALSE)$stdres

No Yes

DCT 2.889222 -2.889222

Index 1.602871 -1.602871

MCT 3.680628 -3.680628

Self -4.438956 4.438956

Unknown -4.257593 4.257593

> sig_test<- table(new$method_notification_multi, new$Uitslag_PCR)

> sigadj_test<-sig/(nrow(sig_test)*ncol(sig_test))

> qnorm(sigadj_test/2)

[1] -2.807034

> chisq.test(sig_test, correct= FALSE)$stdres

Negative Positive

DCT 3.2152901 -3.2152901

Index 0.8532779 -0.8532779

MCT -1.2219416 1.2219416

Self -2.1898578 2.1898578

Unknown -0.4653853 0.4653853

> #calculations interval exposure to testing in dataset with those with exp date!

> new_compint<- new %>%

+ filter(int_exp_test >= 0)

> summary_table <- new_compint %>%

+ group_by(method_notification_multi) %>%

+ summarise(

+ cases = n(),

+ prc_case = scales::percent(cases/3646 , accuracy = 0.01L),

+ all = round(mean(int_exp_test, na.rm = T), digits=2),

+ all_sd = round(sd(int_exp_test, na.rm = T), digits=2),

+ symp_no = round(mean(int_exp_test [symptoms_quest== "No"], na.rm = T), digits = 2),

+ symp_no_sd = round(sd(int_exp_test [symptoms_quest== "No"], na.rm = T), digits = 2),

+ symp_yes = round(mean(int_exp_test [symptoms_quest== "Yes"], na.rm = T), digits = 2),

+ symp_yes_sd = round(sd(int_exp_test [symptoms_quest== "Yes"], na.rm = T), digits = 2),

+ neg_test= round(mean(int_exp_test [Uitslag_PCR == "Negative"], na.rm = T), digits = 2),

+ neg_sd= round(sd(int_exp_test [Uitslag_PCR == "Negative"], na.rm = T), digits = 2),

+ pos_test= round(mean(int_exp_test [Uitslag_PCR == "Positive"], na.rm = T), digits = 2),

+ pos_sd= round(sd(int_exp_test [Uitslag_PCR == "Positive"], na.rm = T), digits = 2),

+ age_1 = round(mean(int_exp_test [agegrp == "16-29"], na.rm = T), digits = 2),

+ age_1_sd = round(sd(int_exp_test [agegrp == "16-29"], na.rm = T), digits = 2),

+ age_2 = round(mean(int_exp_test [agegrp == "30-44"], na.rm = T), digits = 2),

+ age_2_sd = round(sd(int_exp_test [agegrp == "30-44"], na.rm = T), digits = 2),

+ age_3 = round(mean(int_exp_test [agegrp == "45-59"], na.rm = T), digits = 2),

+ age_3_sd = round(sd(int_exp_test [agegrp == "45-59"], na.rm = T), digits = 2),

+ age_4 = round(mean(int_exp_test [agegrp == "60+"], na.rm = T), digits = 2),

+ age_4_sd = round(sd(int_exp_test [agegrp == "60+"], na.rm = T), digits = 2),

+ female = round(mean(int_exp_test [Geslacht == "Female"], na.rm = T), digits = 2),

+ female_sd= round(sd(int_exp_test [Geslacht == "Female"], na.rm = T), digits = 2),

+ male= round(mean(int_exp_test [Geslacht == "Male"], na.rm = T), digits = 2),

+ male_sd= round(sd(int_exp_test [Geslacht == "Male"], na.rm = T), digits = 2),

+ BRA = round(mean(int_exp_test [locationRB == "Brabant"], na.rm = T), digits = 2),

+ BRA_sd = round(sd(int_exp_test [locationRB == "Brabant"], na.rm = T), digits = 2),

+ ROT = round(mean(int_exp_test [locationRB == "Rotterdam"], na.rm = T), digits = 2),

+ ROT_sd = round(sd(int_exp_test [locationRB == "Rotterdam"], na.rm = T), digits = 2), )

> summary_table <- new_compint %>%

+ group_by() %>%

+ summarise(

+ cases = n(),

+ prc_case = scales::percent(cases/3646 , accuracy = 0.01L),

+ tot_symp =sum(symptoms_quest== "Yes" | symptoms_quest== "No", na.rm= T),

+ no_symp = sum(symptoms_quest== "No", na.rm = T),

+ prc_no = scales::percent(no_symp/ tot_symp),

+ yes_symp = sum(symptoms_quest== "Yes", na.rm = T),

+ prc_yes = scales::percent(yes_symp/ tot_symp),

+ tot_test_result =sum(Uitslag_PCR== "Positive" | Uitslag_PCR == "Negative", na.rm= T),

+ neg_test_result = sum(Uitslag_PCR == "Negative", na.rm = T),

+ neg_prc_test_restult = scales::percent(neg_test_result/ tot_test_result,),

+ pos_test_result = sum(Uitslag_PCR== "Positive", na.rm = T),

+ pos_prc_test_restult = scales::percent(pos_test_result/ tot_test_result,),

+ allage= sum(Leeftijd>= 0, na.rm = T),

+ age_0 = sum(agegrp == "16-29", na.rm = T),

+ age_0_pc = scales::percent (age_0/allage),

+ age_1 = sum(agegrp == "30-44", na.rm = T),

+ age_1_pc = scales::percent (age_1/allage),

+ age_3 = sum(agegrp == "45-59", na.rm = T),

+ age_3_pc = scales::percent (age_3/allage),

+ age_4 = sum(agegrp == "60+", na.rm = T),

+ age_4_pc = scales::percent (age_4/allage),

+ totgender= sum(Geslacht== "Female" | Geslacht == "Male", na.rm=T),

+ female =sum(Geslacht== "Female", na.rm = T),

+ female_pc = scales::percent(female/ totgender),

+ male = sum(Geslacht == "Male", na.rm=T),

+ male_pc= scales::percent(male/ totgender),

+ MUN = sum(locationRB == "Brabant" | locationRB == "Rotterdam", na.rm = T),

+ BRA =sum(locationRB == "Brabant" , na.rm = T),

+ BRA_pc = scales::percent (BRA/MUN),

+ ROT =sum(locationRB == "Rotterdam", na.rm = T),

+ ROT_pc = scales::percent (ROT/MUN), )

> #releveling data to set reference group

> new_compint$W_app[new_compint$W_app == "0"] <- "No"

> new_compint$W_app[new_compint$W_app == "10"] <- "Yes"

> new_compint$W_GGD[new_compint$W_GGD == "0"] <- "No"

> new_compint$W_GGD[new_compint$W_GGD == "1"] <- "Yes"

> new_compint$W_pers[new_compint$W_pers == "0"] <- "No"

> new_compint$W_pers[new_compint$W_pers == "100"] <- "Yes"

> new_compint$W_ini [new_compint$W_ini == "0"] <- "No"

> new_compint$W_ini[new_compint$W_ini == "1000"] <- "Yes"

> new_compint$W_unknown<- ifelse(new_compint$method_notification=="Unknown", "Yes", "No")

> new_compint$locationRB <- as.factor(new_compint$locationRB)

> new_compint$int_exp_test_plus1<- new_compint$int_exp_test + 1

> new_compint$status<- 1

> #Univariate Weibull regression model

> occ<-survreg(Surv(int_exp_test_plus1, status)~ W_app,

+ data= new_compint, dist='weibull')

> summary(occ)

Call:

survreg(formula = Surv(int_exp_test_plus1, status) ~ W_app, data = new_compint,

dist = "weibull")

Value Std. Error z p

(Intercept) 1.8593 0.0043 432.1 <2e-16

W_appYes 0.0415 0.0148 2.8 0.0051

Log(scale) -1.4252 0.0116 -122.8 <2e-16

Scale= 0.24

Weibull distribution

Loglik(model)= -6842.5 Loglik(intercept only)= -6846.6

Chisq= 8.19 on 1 degrees of freedom, p= 0.0042

Number of Newton-Raphson Iterations: 5

n= 3646

> ConvertWeibull(occ, conf.level = 0.95)

$vars

Estimate SE

lambda 0.0004384068 4.211433e-05

gamma 4.1588064578 4.826028e-02

W_appYes -0.1726584143 6.179588e-02

$HR

HR LB UB

W_appYes 0.841425 0.7454434 0.949765

$ETR

ETR LB UB

W_appYes 1.04239 1.012509 1.073153

> occ<-survreg(Surv(int_exp_test_plus1, status)~ W_GGD,

+ data= new_compint, dist='weibull')

> summary(occ)

Call:

survreg(formula = Surv(int_exp_test_plus1, status) ~ W_GGD, data = new_compint,

dist = "weibull")

Value Std. Error z p

(Intercept) 1.85922 0.00447 416.09 <2e-16

W_GGDYes 0.02276 0.01134 2.01 0.045

Log(scale) -1.42386 0.01158 -122.97 <2e-16

Scale= 0.241

Weibull distribution

Loglik(model)= -6844.6 Loglik(intercept only)= -6846.6

Chisq= 4.12 on 1 degrees of freedom, p= 0.042

Number of Newton-Raphson Iterations: 5

n= 3646

> ConvertWeibull(occ, conf.level = 0.95)

$vars

Estimate SE

lambda 0.0004431684 4.253095e-05

gamma 4.1531072692 4.809012e-02

W_GGDYes -0.0945378655 4.711246e-02

$ΩR

HR LB UB

W_GGDYes 0.9097933 0.8295461 0.9978033

$ETR

ETR LB UB

W_GGDYes 1.023024 1.000537 1.046017

> occ<-survreg(Surv(int_exp_test_plus1, status)~ W_pers,

+ data= new_compint, dist='weibull')

> summary(occ)

Call:

survreg(formula = Surv(int_exp_test_plus1, status) ~ W_pers,

data = new_compint, dist = "weibull")

Value Std. Error z p

(Intercept) 1.89628 0.00775 244.6 <2e-16

W_persYes -0.04626 0.00889 -5.2 2e-07

Log(scale) -1.43415 0.01177 -121.8 <2e-16

Scale= 0.238

Weibull distribution

Loglik(model)= -6833 Loglik(intercept only)= -6846.6

Chisq= 27.32 on 1 degrees of freedom, p= 1.7e-07

Number of Newton-Raphson Iterations: 6

n= 3646

> ConvertWeibull(occ, conf.level = 0.95)

$vars

Estimate SE

lambda 0.0003502284 3.728595e-05

gamma 4.1960717293 4.940189e-02

W_persYes 0.1941010997 3.763350e-02

$HR

HR LB UB

W_persYes 1.214219 1.127881 1.307166

$ETR

ETR LB UB

W_persYes 0.9547958 0.9383043 0.9715771

> occ<-survreg(Surv(int_exp_test_plus1, status)~ W_ini,

+ data= new_compint, dist='weibull')

> summary(occ)

Call:

survreg(formula = Surv(int_exp_test_plus1, status) ~ W_ini, data = new_compint,

dist = "weibull")

Value Std. Error z p

(Intercept) 1.8603 0.0044 423.25 <2e-16

W_iniYes 0.0183 0.0118 1.54 0.12

Log(scale) -1.4253 0.0117 -122.31 <2e-16

Scale= 0.24

Weibull distribution

Loglik(model)= -6845.4 Loglik(intercept only)= -6846.6

Chisq= 2.42 on 1 degrees of freedom, p= 0.12

Number of Newton-Raphson Iterations: 6

n= 3646

> ConvertWeibull(occ, conf.level = 0.95)

$vars

Estimate SE

lambda 0.0004362431 0.0000419289

gamma 4.1591392858 0.0484673290

W_iniYes -0.0760726910 0.0493348780

$HR

HR LB UB

W_iniYes 0.9267488 0.8413334 1.020836

$ETR

ETR LB UB

W_iniYes 1.018459 0.9950969 1.042369

> occ<-survreg(Surv(int_exp_test_plus1, status)~ W_unknown,

+ data= new_compint, dist='weibull')

> summary(occ)

Call:

survreg(formula = Surv(int_exp_test_plus1, status) ~ W_unknown,

data = new_compint, dist = "weibull")

Value Std. Error z p

(Intercept) 1.86253 0.00417 446.42 <2e-16

W_unknownYes 0.00841 0.04836 0.17 0.86

Log(scale) -1.42328 0.01158 -122.91 <2e-16

Scale= 0.241

Weibull distribution

Loglik(model)= -6846.6 Loglik(intercept only)= -6846.6

Chisq= 0.03 on 1 degrees of freedom, p= 0.86

Number of Newton-Raphson Iterations: 6

n= 3646

> ConvertWeibull(occ, conf.level = 0.95)

$vars

Estimate SE

lambda 0.000439078 4.208262e-05

gamma 4.150707871 4.806399e-02

W_unknownYes -0.034905912 2.007151e-01

$HR

HR LB UB

W_unknownYes 0.9656963 0.6516157 1.431164

$ETR

ETR LB UB

W_unknownYes 1.008445 0.9172592 1.108696

> occ<-survreg(Surv(int_exp_test_plus1, status)~ symptoms_quest,

+ data= new_compint, dist='weibull')

> summary(occ)

Call:

survreg(formula = Surv(int_exp_test_plus1, status) ~ symptoms_quest,

data = new_compint, dist = "weibull")

Value Std. Error z p

(Intercept) 1.8614 0.0043 432.42 <2e-16

symptoms_questYes 0.0216 0.0147 1.47 0.14

Log(scale) -1.4255 0.0117 -122.29 <2e-16

Scale= 0.24

Weibull distribution

Loglik(model)= -6802.7 Loglik(intercept only)= -6803.8

Chisq= 2.21 on 1 degrees of freedom, p= 0.14

Number of Newton-Raphson Iterations: 6

n=3625 (21 observations deleted due to missingness)

> ConvertWeibull(occ, conf.level = 0.95)

$vars

Estimate SE

lambda 0.0004337074 4.178994e-05

gamma 4.1598622625 4.848790e-02

symptoms_questYes -0.0898704750 6.121810e-02

$HR

HR LB UB

symptoms_questYes 0.9140496 0.8107012 1.030573

$ETR

ETR LB UB

symptoms_questYes 1.021839 0.9928297 1.051696

> occ<-survreg(Surv(int_exp_test_plus1, status)~ Uitslag_PCR,

+ data= new_compint, dist='weibull')

> summary(occ)

Call:

survreg(formula = Surv(int_exp_test_plus1, status) ~ Uitslag_PCR,

data = new_compint, dist = "weibull")

Value Std. Error z p

(Intercept) 1.86394 0.00433 430.86 <2e-16

Uitslag_PCRPositive -0.01670 0.01442 -1.16 0.25

Log(scale) -1.42318 0.01158 -122.89 <2e-16

Scale= 0.241

Weibull distribution

Loglik(model)= -6846 Loglik(intercept only)= -6846.6

Chisq= 1.32 on 1 degrees of freedom, p= 0.25

Number of Newton-Raphson Iterations: 6

n= 3646

> ConvertWeibull(occ, conf.level = 0.95)

$vars

Estimate SE

lambda 0.000436865 4.192336e-05

gamma 4.150296521 4.806525e-02

Uitslag_PCRPositive 0.069308003 5.981716e-02

$HR

HR LB UB

Uitslag_PCRPositive 1.071766 0.9531991 1.205082

$ETR

ETR LB UB

Uitslag_PCRPositive 0.9834391 0.9560429 1.01162

> occ<-survreg(Surv(int_exp_test_plus1, status)~ agegrp,

+ data= new_compint, dist='weibull')

> summary(occ)

Call:

survreg(formula = Surv(int_exp_test_plus1, status) ~ agegrp,

data = new_compint, dist = "weibull")

Value Std. Error z p

(Intercept) 1.851529 0.007723 239.75 <2e-16

agegrp30-44 0.017159 0.011275 1.52 0.1280

agegrp45-59 0.000647 0.010584 0.06 0.9513

agegrp60+ 0.034301 0.011775 2.91 0.0036

Log(scale) -1.425358 0.011621 -122.65 <2e-16

Scale= 0.24

Weibull distribution

Loglik(model)= -6826.3 Loglik(intercept only)= -6832.1

Chisq= 11.53 on 3 degrees of freedom, p= 0.0092

Number of Newton-Raphson Iterations: 5

n=3637 (9 observations deleted due to missingness)

> ConvertWeibull(occ, conf.level = 0.95)

$vars

Estimate SE

lambda 0.0004523055 4.475752e-05

gamma 4.1593476809 4.833549e-02

agegrp30-44 -0.0713690375 4.691296e-02

agegrp45-59 -0.0026903254 4.402515e-02

agegrp60+ -0.1426695156 4.906148e-02

$HR

HR LB UB

agegrp30-44 0.9311182 0.8493221 1.0207919

agegrp45-59 0.9973133 0.9148657 1.0871911

agegrp60+ 0.8670406 0.7875501 0.9545543

$ETR

ETR LB UB

agegrp30-44 1.017307 0.9950723 1.040038

agegrp45-59 1.000647 0.9801022 1.021622

agegrp60+ 1.034896 1.0112866 1.059057

> occ<-survreg(Surv(int_exp_test_plus1, status)~ Geslacht,

+ data= new_compint, dist='weibull')

> summary(occ)

Call:

survreg(formula = Surv(int_exp_test_plus1, status) ~ Geslacht,

data = new_compint, dist = "weibull")

Value Std. Error z p

(Intercept) 1.862492 0.005820 320.03 <2e-16

GeslachtMale 0.000343 0.008006 0.04 0.97

Log(scale) -1.422091 0.011607 -122.52 <2e-16

Scale= 0.241

Weibull distribution

Loglik(model)= -6825.1 Loglik(intercept only)= -6825.1

Chisq= 0 on 1 degrees of freedom, p= 0.97

Number of Newton-Raphson Iterations: 5

n=3632 (14 observations deleted due to missingness)

> ConvertWeibull(occ, conf.level = 0.95)

$vars

Estimate SE

lambda 0.0004432037 4.314403e-05

gamma 4.1457797170 4.811832e-02

GeslachtMale -0.0014213728 3.319037e-02

$HR

HR LB UB

GeslachtMale 0.9985796 0.9356879 1.065699

$ETR

ETR LB UB

GeslachtMale 1.000343 0.984769 1.016163

> occ<-survreg(Surv(int_exp_test_plus1, status)~ locationRB,

+ data= new_compint, dist='weibull')

> summary(occ)

Call:

survreg(formula = Surv(int_exp_test_plus1, status) ~ locationRB,

data = new_compint, dist = "weibull")

Value Std. Error z p

(Intercept) 1.87924 0.00532 353.55 < 2e-16

locationRBRotterdam -0.04158 0.00813 -5.12 3.1e-07

Log(scale) -1.43126 0.01171 -122.18 < 2e-16

Scale= 0.239

Weibull distribution

Loglik(model)= -6819.4 Loglik(intercept only)= -6832.1

Chisq= 25.49 on 1 degrees of freedom, p= 4.4e-07

Number of Newton-Raphson Iterations: 6

n=3637 (9 observations deleted due to missingness)

> ConvertWeibull(occ, conf.level = 0.95)

$vars

Estimate SE

lambda 0.0003848523 3.868447e-05

gamma 4.1839502705 4.901296e-02

locationRBRotterdam 0.1739685450 3.424891e-02

$HR

HR LB UB

locationRBRotterdam 1.190018 1.112758 1.272642

$ETR

ETR LB UB

locationRBRotterdam 0.9592726 0.944113 0.9746757

> #Multivariable Weibull regression model

> occ<-survreg(Surv(int_exp_test_plus1, status)~ W_app + W_GGD + W_pers + W_ini + W_unknown + symptoms_quest + Uitslag_PCR + agegrp + Geslacht + locationRB,

+ data= new_compint, dist='weibull')

> summary(occ)

Call:

survreg(formula = Surv(int_exp_test_plus1, status) ~ W_app +

W_GGD + W_pers + W_ini + W_unknown + symptoms_quest + Uitslag_PCR +

agegrp + Geslacht + locationRB, data = new_compint, dist = "weibull")

Value Std. Error z p

(Intercept) 1.911056 0.019218 99.44 <2e-16

W_appYes 0.000980 0.019009 0.05 0.9589

W_GGDYes -0.004291 0.014234 -0.30 0.7631

W_persYes -0.052747 0.016325 -3.23 0.0012

W_iniYes -0.027024 0.019225 -1.41 0.1598

W_unknownYes -0.033385 0.051147 -0.65 0.5139

symptoms_questYes 0.032496 0.014704 2.21 0.0271

Uitslag_PCRPositive -0.016268 0.014254 -1.14 0.2538

agegrp30-44 0.017993 0.011157 1.61 0.1068

agegrp45-59 -0.005809 0.010528 -0.55 0.5811

agegrp60+ 0.024351 0.011908 2.04 0.0409

GeslachtMale -0.000829 0.007918 -0.10 0.9166

locationRBRotterdam -0.034083 0.008403 -4.06 5e-05

Log(scale) -1.443627 0.011999 -120.31 <2e-16

Scale= 0.236

Weibull distribution

Loglik(model)= -6750.9 Loglik(intercept only)= -6782.2

Chisq= 62.6 on 12 degrees of freedom, p= 7.6e-09

Number of Newton-Raphson Iterations: 7

n=3611 (35 observations deleted due to missingness)

> ConvertWeibull(occ, conf.level = 0.95)

$vars

Estimate SE

lambda 0.000304971 4.034555e-05

gamma 4.236031947 5.082815e-02

W_appYes -0.004152766 8.052265e-02

W_GGDYes 0.018177050 6.029882e-02

W_persYes 0.223436417 6.935274e-02

W_iniYes 0.114474538 8.142928e-02

W_unknownYes 0.141420001 2.166705e-01

symptoms_questYes -0.137654780 6.244492e-02

Uitslag_PCRPositive 0.068910798 6.037040e-02

agegrp30-44 -0.076217516 4.729473e-02

agegrp45-59 0.024607437 4.459584e-02

agegrp60+ -0.103151360 5.049402e-02

GeslachtMale 0.003511860 3.353981e-02

locationRBRotterdam 0.144378442 3.574673e-02

$HR

HR LB UB

W_appYes 0.9958558 0.8504631 1.1661045

W_GGDYes 1.0183433 0.9048316 1.1460951

W_persYes 1.2503661 1.0914505 1.4324200

W_iniYes 1.1212841 0.9558790 1.3153108

W_unknownYes 1.1519083 0.7533341 1.7613603

symptoms_questYes 0.8713995 0.7710172 0.9848509

Uitslag_PCRPositive 1.0713406 0.9517880 1.2059102

agegrp30-44 0.9266146 0.8445820 1.0166150

agegrp45-59 1.0249127 0.9391324 1.1185281

agegrp60+ 0.9019904 0.8169987 0.9958238

GeslachtMale 1.0035180 0.9396715 1.0717027

locationRBRotterdam 1.1553212 1.0771473 1.2391687

$ETR

ETR LB UB

W_appYes 1.0009808 0.9643733 1.0389780

W_GGDYes 0.9957181 0.9683243 1.0238870

W_persYes 0.9486203 0.9187478 0.9794642

W_iniYes 0.9733379 0.9373452 1.0107126

W_unknownYes 0.9671661 0.8749121 1.0691477

symptoms_questYes 1.0330299 1.0036829 1.0632350

Uitslag_PCRPositive 0.9838638 0.9567574 1.0117383

agegrp30-44 1.0181555 0.9961335 1.0406643

agegrp45-59 0.9942078 0.9739027 1.0149362

agegrp60+ 1.0246498 1.0010118 1.0488461

GeslachtMale 0.9991713 0.9837853 1.0147979

locationRBRotterdam 0.9664909 0.9507034 0.9825405

> #Linear model with tobit

> occ <- vglm(int_exp_test ~ W_app,

+ family= tobit(Lower = 0, Upper = 14), data = new_compint)

> summary(occ)

Call:

vglm(formula = int_exp_test ~ W_app, family = tobit(Lower = 0,

Upper = 14), data = new_compint)

Coefficients:

Estimate Std. Error z value Pr(>|z|)

(Intercept):1 4.89319 0.02700 181.219 < 2e-16 ***

(Intercept):2 0.44809 0.01172 38.236 < 2e-16 ***

W_appYes 0.27151 0.09658 2.811 0.00493 **

---

Signif. codes: 0 ‘***’ 0.001 ‘**’ 0.01 ‘*’ 0.05 ‘.’ 0.1 ‘ ’ 1

Names of linear predictors: mu, loglink(sd)

Log-likelihood: -6787.037 on 7289 degrees of freedom

Number of Fisher scoring iterations: 5

No Hauck-Donner effect found in any of the estimates

> b <- coef(occ)

> se <- sqrt(diag(vcov(occ)))

> cbind(LL = b - qnorm(0.975) * se, UL = b + qnorm(0.975) * se)

LL UL

(Intercept):1 4.8402672 4.9461113

(Intercept):2 0.4251254 0.4710643

W_appYes 0.0822246 0.4607948

> occ <- vglm(int_exp_test ~ W_GGD,

+ family= tobit(Lower = 0, Upper = 14), data = new_compint)

> summary(occ)

Call:

vglm(formula = int_exp_test ~ W_GGD, family = tobit(Lower = 0,

Upper = 14), data = new_compint)

Coefficients:

Estimate Std. Error z value Pr(>|z|)

(Intercept):1 4.88809 0.02804 174.346 <2e-16 ***

(Intercept):2 0.44835 0.01172 38.257 <2e-16 ***

W_GGDYes 0.18208 0.07374 2.469 0.0135 *

---

Signif. codes: 0 ‘***’ 0.001 ‘**’ 0.01 ‘*’ 0.05 ‘.’ 0.1 ‘ ’ 1

Names of linear predictors: mu, loglink(sd)

Log-likelihood: -6787.939 on 7289 degrees of freedom

Number of Fisher scoring iterations: 5

No Hauck-Donner effect found in any of the estimates

> b <- coef(occ)

> se <- sqrt(diag(vcov(occ)))

> cbind(LL = b - qnorm(0.975) * se, UL = b + qnorm(0.975) * se)

LL UL

(Intercept):1 4.83313871 4.9430406

(Intercept):2 0.42538298 0.4713219

W_GGDYes 0.03754702 0.3266177

> occ <- vglm(int_exp_test ~ W_pers,

+ family= tobit(Lower = 0, Upper = 14), data = new_compint)

> summary(occ)

Call:

vglm(formula = int_exp_test ~ W_pers, family = tobit(Lower = 0,

Upper = 14), data = new_compint)

Coefficients:

Estimate Std. Error z value Pr(>|z|)

(Intercept):1 4.80459 0.04937 97.323 < 2e-16 ***

(Intercept):2 0.44836 0.01172 38.258 < 2e-16 ***

W_persYes 0.15161 0.05802 2.613 0.00897 **

---

Signif. codes: 0 ‘***’ 0.001 ‘**’ 0.01 ‘*’ 0.05 ‘.’ 0.1 ‘ ’ 1

Names of linear predictors: mu, loglink(sd)

Log-likelihood: -6787.58 on 7289 degrees of freedom

Number of Fisher scoring iterations: 5

No Hauck-Donner effect found in any of the estimates

> b <- coef(occ)

> se <- sqrt(diag(vcov(occ)))

> cbind(LL = b - qnorm(0.975) * se, UL = b + qnorm(0.975) * se)

LL UL

(Intercept):1 4.70783011 4.9013467

(Intercept):2 0.42538613 0.4713252

W_persYes 0.03789754 0.2653147

> occ <- vglm(int_exp_test ~ W_ini,

+ family= tobit(Lower = 0, Upper = 14), data = new_compint)

> summary(occ)

Call:

vglm(formula = int_exp_test ~ W_ini, family = tobit(Lower = 0,

Upper = 14), data = new_compint)

Coefficients:

Estimate Std. Error z value Pr(>|z|)

(Intercept):1 4.98775 0.02765 180.361 < 2e-16 ***

(Intercept):2 0.44226 0.01172 37.737 < 2e-16 ***

W_iniYes -0.55959 0.07630 -7.334 2.23e-13 ***

---

Signif. codes: 0 ‘***’ 0.001 ‘**’ 0.01 ‘*’ 0.05 ‘.’ 0.1 ‘ ’ 1

Names of linear predictors: mu, loglink(sd)

Log-likelihood: -6764.428 on 7289 degrees of freedom

Number of Fisher scoring iterations: 5

No Hauck-Donner effect found in any of the estimates

> b <- coef(occ)

> se <- sqrt(diag(vcov(occ)))

> cbind(LL = b - qnorm(0.975) * se, UL = b + qnorm(0.975) * se)

LL UL

(Intercept):1 4.9335533 5.0419561

(Intercept):2 0.4192909 0.4652306

W_iniYes -0.7091324 -0.4100405

> occ <- vglm(int_exp_test ~ W_unknown,

+ family= tobit(Lower = 0, Upper = 14), data = new_compint)

> summary(occ)

Call:

vglm(formula = int_exp_test ~ W_unknown, family = tobit(Lower = 0,

Upper = 14), data = new_compint)

Coefficients:

Estimate Std. Error z value Pr(>|z|)

(Intercept):1 4.92037 0.02602 189.109 < 2e-16 ***

(Intercept):2 0.44827 0.01172 38.251 < 2e-16 ***

W_unknownYes -0.87897 0.31427 -2.797 0.00516 **

---

Signif. codes: 0 ‘***’ 0.001 ‘**’ 0.01 ‘*’ 0.05 ‘.’ 0.1 ‘ ’ 1

Names of linear predictors: mu, loglink(sd)

Log-likelihood: -6787.144 on 7289 degrees of freedom

Number of Fisher scoring iterations: 5

No Hauck-Donner effect found in any of the estimates

> b <- coef(occ)

> se <- sqrt(diag(vcov(occ)))

> cbind(LL = b - qnorm(0.975) * se, UL = b + qnorm(0.975) * se)

LL UL

(Intercept):1 4.8693718 4.9713633

(Intercept):2 0.4253046 0.4712439

W_unknownYes -1.4949312 -0.2630154

> occ <- vglm(int_exp_test ~ symptoms_quest,

+ family= tobit(Lower = 0, Upper = 14), data = new_compint)

> summary(occ)

Call:

vglm(formula = int_exp_test ~ symptoms_quest, family = tobit(Lower = 0,

Upper = 14), data = new_compint)

Coefficients:

Estimate Std. Error z value Pr(>|z|)

(Intercept):1 4.95357 0.02703 183.292 < 2e-16 ***

(Intercept):2 0.44466 0.01175 37.833 < 2e-16 ***

symptoms_questYes -0.44656 0.09506 -4.697 2.63e-06 ***

---

Signif. codes: 0 ‘***’ 0.001 ‘**’ 0.01 ‘*’ 0.05 ‘.’ 0.1 ‘ ’ 1

Names of linear predictors: mu, loglink(sd)

Log-likelihood: -6735.867 on 7247 degrees of freedom

Number of Fisher scoring iterations: 5

No Hauck-Donner effect found in any of the estimates

> b <- coef(occ)

> se <- sqrt(diag(vcov(occ)))

> cbind(LL = b - qnorm(0.975) * se, UL = b + qnorm(0.975) * se)

LL UL

(Intercept):1 4.9006053 5.0065440

(Intercept):2 0.4216243 0.4676956

symptoms_questYes -0.6328832 -0.2602399

> occ <- vglm(int_exp_test ~ Uitslag_PCR,

+ family= tobit(Lower = 0, Upper = 14), data = new_compint)

> summary(occ)

Call:

vglm(formula = int_exp_test ~ Uitslag_PCR, family = tobit(Lower = 0,

Upper = 14), data = new_compint)

Coefficients:

Estimate Std. Error z value Pr(>|z|)

(Intercept):1 4.94980 0.02704 183.073 < 2e-16 ***

(Intercept):2 0.44644 0.01172 38.094 < 2e-16 ***

Uitslag_PCRPositive -0.42339 0.09348 -4.529 5.93e-06 ***

---

Signif. codes: 0 ‘***’ 0.001 ‘**’ 0.01 ‘*’ 0.05 ‘.’ 0.1 ‘ ’ 1

Names of linear predictors: mu, loglink(sd)

Log-likelihood: -6780.801 on 7289 degrees of freedom

Number of Fisher scoring iterations: 5

No Hauck-Donner effect found in any of the estimates

> b <- coef(occ)

> se <- sqrt(diag(vcov(occ)))

> cbind(LL = b - qnorm(0.975) * se, UL = b + qnorm(0.975) * se)

LL UL

(Intercept):1 4.8968108 5.002795

(Intercept):2 0.4234678 0.469407

Uitslag_PCRPositive -0.6066168 -0.240165

> occ <- vglm(int_exp_test ~ agegrp,

+ family= tobit(Lower = 0, Upper = 14), data = new_compint)

> summary(occ)

Call:

vglm(formula = int_exp_test ~ agegrp, family = tobit(Lower = 0,

Upper = 14), data = new_compint)

Coefficients:

Estimate Std. Error z value Pr(>|z|)

(Intercept):1 4.92410 0.04989 98.708 <2e-16 ***

(Intercept):2 0.44877 0.01173 38.246 <2e-16 ***

agegrp30-44 0.06133 0.07346 0.835 0.404

agegrp45-59 -0.11193 0.06895 -1.623 0.105

agegrp60+ 0.04766 0.07667 0.622 0.534

---

Signif. codes: 0 ‘***’ 0.001 ‘**’ 0.01 ‘*’ 0.05 ‘.’ 0.1 ‘ ’ 1

Names of linear predictors: mu, loglink(sd)

Log-likelihood: -6772.782 on 7269 degrees of freedom

Number of Fisher scoring iterations: 5

No Hauck-Donner effect found in any of the estimates

> b <- coef(occ)

> se <- sqrt(diag(vcov(occ)))

> cbind(LL = b - qnorm(0.975) * se, UL = b + qnorm(0.975) * se)

LL UL

(Intercept):1 4.82632111 5.02186932

(Intercept):2 0.42577060 0.47176661

agegrp30-44 -0.08263716 0.20530634

agegrp45-59 -0.24707145 0.02321337

agegrp60+ -0.10260095 0.19792492

> occ <- vglm(int_exp_test ~ Geslacht,

+ family= tobit(Lower = 0, Upper = 14), data = new_compint)

> summary(occ)

Call:

vglm(formula = int_exp_test ~ Geslacht, family = tobit(Lower = 0,

Upper = 14), data = new_compint)

Coefficients:

Estimate Std. Error z value Pr(>|z|)

(Intercept):1 4.94284 0.03710 133.24 <2e-16 ***

(Intercept):2 0.45043 0.01174 38.36 <2e-16 ***

GeslachtMale -0.05622 0.05208 -1.08 0.28

---

Signif. codes: 0 ‘***’ 0.001 ‘**’ 0.01 ‘*’ 0.05 ‘.’ 0.1 ‘ ’ 1

Names of linear predictors: mu, loglink(sd)

Log-likelihood: -6769.013 on 7261 degrees of freedom

Number of Fisher scoring iterations: 5

No Hauck-Donner effect found in any of the estimates

> b <- coef(occ)

> se <- sqrt(diag(vcov(occ)))

> cbind(LL = b - qnorm(0.975) * se, UL = b + qnorm(0.975) * se)

LL UL

(Intercept):1 4.8701337 5.01554876

(Intercept):2 0.4274186 0.47344652

GeslachtMale -0.1582872 0.04584925

> occ <- vglm(int_exp_test ~ locationRB,

+ family= tobit(Lower = 0, Upper = 14), data = new_compint)

> summary(occ)

Call:

vglm(formula = int_exp_test ~ locationRB, family = tobit(Lower = 0,

Upper = 14), data = new_compint)

Coefficients:

Estimate Std. Error z value Pr(>|z|)

(Intercept):1 4.88182 0.03352 145.62 <2e-16 ***

(Intercept):2 0.44962 0.01173 38.32 <2e-16 ***

locationRBRotterdam 0.08177 0.05309 1.54 0.124

---

Signif. codes: 0 ‘***’ 0.001 ‘**’ 0.01 ‘*’ 0.05 ‘.’ 0.1 ‘ ’ 1

Names of linear predictors: mu, loglink(sd)

Log-likelihood: -6775.264 on 7271 degrees of freedom

Number of Fisher scoring iterations: 5

No Hauck-Donner effect found in any of the estimates

> b <- coef(occ)

> se <- sqrt(diag(vcov(occ)))

> cbind(LL = b - qnorm(0.975) * se, UL = b + qnorm(0.975) * se)

LL UL

(Intercept):1 4.81610951 4.9475224

(Intercept):2 0.42661892 0.4726150

locationRBRotterdam -0.02229259 0.1858324

> occ <- vglm(int_exp_test ~ W_app + W_GGD + W_pers + W_ini + W_unknown + symptoms_quest + Uitslag_PCR + agegrp + Geslacht + locationRB,

+ family= tobit(Lower = 0, Upper = 14), data = new_compint)

> summary(occ)

Call:

vglm(formula = int_exp_test ~ W_app + W_GGD + W_pers + W_ini +

W_unknown + symptoms_quest + Uitslag_PCR + agegrp + Geslacht +

locationRB, family = tobit(Lower = 0, Upper = 14), data = new_compint)

Coefficients:

Estimate Std. Error z value Pr(>|z|)

(Intercept):1 5.16712 0.12453 41.493 < 2e-16 ***

(Intercept):2 0.43448 0.01178 36.894 < 2e-16 ***

W_appYes 0.08068 0.12334 0.654 0.513042

W_GGDYes 0.04309 0.09226 0.467 0.640482

W_persYes -0.13743 0.10462 -1.314 0.188965

W_iniYes -0.62708 0.12302 -5.097 3.44e-07 ***

W_unknownYes -0.83427 0.33369 -2.500 0.012414 *

symptoms_questYes -0.36001 0.09550 -3.770 0.000164 ***

Uitslag_PCRPositive -0.36131 0.09358 -3.861 0.000113 ***

agegrp30-44 0.04418 0.07293 0.606 0.544611

agegrp45-59 -0.10166 0.06880 -1.478 0.139527

agegrp60+ 0.05666 0.07710 0.735 0.462454

GeslachtMale -0.05145 0.05163 -0.997 0.318988

locationRBRotterdam 0.04852 0.05410 0.897 0.369855

---

Signif. codes: 0 ‘***’ 0.001 ‘**’ 0.01 ‘*’ 0.05 ‘.’ 0.1 ‘ ’ 1

Names of linear predictors: mu, loglink(sd)

Log-likelihood: -6671.086 on 7208 degrees of freedom

Number of Fisher scoring iterations: 5

No Hauck-Donner effect found in any of the estimates

> b <- coef(occ)

> se <- sqrt(diag(vcov(occ)))

> cbind(LL = b - qnorm(0.975) * se, UL = b + qnorm(0.975) * se)

LL UL

(Intercept):1 4.92304709 5.41119129

(Intercept):2 0.41139484 0.45755693

W_appYes -0.16106876 0.32242897

W_GGDYes -0.13774221 0.22391999

W_persYes -0.34247482 0.06761562

W_iniYes -0.86819681 -0.38597243

W_unknownYes -1.48828904 -0.18025174

symptoms_questYes -0.54719054 -0.17282397

Uitslag_PCRPositive -0.54471685 -0.17790323

agegrp30-44 -0.09875347 0.18712187

agegrp45-59 -0.23650898 0.03319070

agegrp60+ -0.09446357 0.20777672

GeslachtMale -0.15264143 0.04974069

locationRBRotterdam -0.05752229 0.15455359

## Second rapid diagnostic test accuracy study

> #Second RDT evaluation study data

> Sys.setlocale("LC_TIME", "en_GB")

[1] "en_GB"

> #install packages

> library(readxl)

> library(dplyr)

> library(tidyverse)

> library(ggplot2)

> library(writexl)

> library(survival)

> library(SurvRegCensCov)

> library(eha)

> library(VGAM)

> #import datasets

> A1 <- read.csv("~/Desktop/CM for paper/Data/Second_antigentestround/data_Wianne_ Abbott fase 1 .csv")

> A2 <- read.csv("~/Desktop/CM for paper/Data/Second_antigentestround/data_Wianne_ Abbott fase 2 .csv")

> R1 <- read.csv("~/Desktop/CM for paper/Data/Second_antigentestround/data_Wianne_ Roche fase 1 .csv")

> R2 <- read.csv("~/Desktop/CM for paper/Data/Second_antigentestround/data_Wianne_ Roche fase 2 .csv")

> V1 <- read.csv("~/Desktop/CM for paper/Data/Second_antigentestround/data_Wianne_ BD-veritor fase 1 .csv")

> #Select important variables

> A1<- A1 %>%

+ select(Afname_dt, Antigeen_sneltest, Uitslag_PCR, Ct, R_klachten, R_contact, dt_lcontact, W_huisgenoot, W_app, W_GGD, W_pers, W_anders, W_anders_nl, R_huisarts, R_reis, R_anders, R_anders_nl, Gevaccineerd, Eerdercorona, Klachten, Kl_sinds, Afspraak_dt, Geslacht, Leeftijd)

> A2<- A2 %>%

+ select(Afname_dt, Antigeen_sneltest, Uitslag_PCR, Ct, R_klachten, R_contact, dt_lcontact, W_huisgenoot, W_app, W_GGD, W_pers, W_anders, W_anders_nl, R_huisarts, R_reis, R_anders, R_anders_nl, Gevaccineerd, Eerdercorona, Klachten, Kl_sinds, Afspraak_dt, Geslacht, Leeftijd)

> R1<- R1 %>%

+ select(Afname_dt, Antigeen_sneltest, Uitslag_PCR, Ct, R_klachten, R_contact, dt_lcontact, W_huisgenoot, W_app, W_GGD, W_pers, W_anders, W_anders_nl, R_huisarts, R_reis, R_anders, R_anders_nl, Gevaccineerd, Eerdercorona, Klachten, Kl_sinds, Afspraak_dt, Geslacht, Leeftijd)

> R2<- R2 %>%

+ select(Afname_dt, Antigeen_sneltest, Uitslag_PCR, Ct, R_klachten, R_contact, dt_lcontact, W_huisgenoot, W_app, W_GGD, W_pers, W_anders, W_anders_nl, R_huisarts, R_reis, R_anders, R_anders_nl, Gevaccineerd, Eerdercorona, Klachten, Kl_sinds, Afspraak_dt, Geslacht, Leeftijd)

> V1<- V1 %>%

+ select(Afname_dt, Antigeen_sneltest, Uitslag_PCR, Ct, R_klachten, R_contact, dt_lcontact, W_huisgenoot, W_app, W_GGD, W_pers, W_anders, W_anders_nl, R_huisarts, R_reis, R_anders, R_anders_nl, Gevaccineerd, Eerdercorona, Klachten, Kl_sinds, Afspraak_dt, Geslacht, Leeftijd)

> #combined to one dataset

> complete_dataset_second_antigenevaluation <- bind_rows(A1, A2, R1, R2, V1, .id= "origins")

> #edit based on text fields reason testing. If they have a reason in the column "W_anders_nl" then recategorize this and add the column W_self. Recategorize column "R_anders_nl" as well.

> write_xlsx(complete_dataset_second_antigenevaluation, "~/Desktop/CM for paper/Data/Second_antigentestround/complete_dataset_second_antigenevaluation.xlsx")

> #import edited file and rename

> comb <- read_excel("~/Desktop/CM for paper/Data/Second_antigentestround/edited_complete_dataset_second_antigenevaluation.xlsx", col_types = c("text", "text", "text", "numeric", "numeric", "numeric", "numeric", "text", "text", "text", "text", "text", "text", "text", "numeric", "numeric", "numeric", "numeric", "numeric", "numeric", "text", "text", "numeric", "text", "text", "text", "numeric"))

> #clean and categorize the reasons for testing after contact

> comb$W_app <- ifelse((comb$W_app == "Ja"), 1, 0)

> comb$W_app<- replace_na(comb$W_app, 0)

> comb$W_GGD <- ifelse((comb$W_GGD == "Ja"), 10, 0)

> comb$W_GGD<- replace_na(comb$W_GGD, 0)

> comb$W_pers <- ifelse((comb$W_pers == "Ja"), 1000, 0)

> comb$W_pers<- replace_na(comb$W_pers, 0)

> comb$W_huisgenoot <- ifelse((comb$W_huisgenoot == "Ja"), 100, 0)

> comb$W_huisgenoot<- replace_na(comb$W_huisgenoot, 0)

> comb$W_self <- ifelse((comb$W_self == "Ja"), 10000, 0)

> comb$W_self<- replace_na(comb$W_self, 0)

> table(comb$W_app)

0 1

7677 248

> table(comb$W_GGD)

0 10

7614 311

> table(comb$W_pers)

0 1000

5856 2069

> table(comb$W_huisgenoot)

0 100

7037 888

> table(comb$W_self)

0 10000

7649 276

> comb$R_contact_added_up<- comb$W_app + comb$W_GGD + comb$W_pers + comb$W_huisgenoot + comb$W_self

> comb$R_contact_added_up<-ifelse(comb$R_contact_added_up == 0, 0, 1)

> comb$R_contact<- replace_na(comb$R_contact, 0)

> comb$R_allcontact<- comb$R_contact + comb$R_contact_added_up

> comb$R_allcontact<-ifelse(comb$R_allcontact == 0, 0, 1)

> table(comb$R_allcontact)

0 1

4191 3734

> #clean and new variable for reason testing in general

> comb$R_klachten<- replace_na(comb$R_klachten, 0)

> comb$R_klachten<- as.numeric(comb$R_klachten)

> comb$R_allcontact[comb$R_allcontact == 1] <- 100

> comb$R_huisarts<- replace_na(comb$R_huisarts, 0)

> comb$R_huisarts<- as.numeric(comb$R_huisarts)

> comb$R_huisarts[comb$R_huisarts == 1] <- 10

> comb$R_reis<- replace_na(comb$R_reis, 0)

> comb$R_reis<- as.numeric(comb$R_reis)

> comb$R_reis[comb$R_reis == 1] <- 1000

> comb$`R_anders(01)`<- replace_na(comb$`R_anders(01)`, 0)

> comb$`R_anders(01)` <- as.numeric(comb$`R_anders(01)`)

> comb$`R_anders(01)`[comb$`R_anders(01)` == 1] <- 10000

> table(comb$R_klachten)

0 1

3741 4184

> table(comb$R_allcontact)

0 100

4191 3734

> table(comb$R_huisarts)

0 10

7759 166

> table(comb$R_reis)

0 1000

7805 120

> table(comb$`R_anders(01)`)

0 10000

7807 118

> #add row number as a sort of id

> comb <- comb %>% mutate(id = row_number())

> #make new variable that names the various combinations of reasons for testing.

> comb$reason_testing_general<- comb$R_klachten + comb$R_allcontact + comb$R_huisarts + comb$R_reis +comb$`R_anders(01)`

> table(comb$reason_testing_general)

0 1 10 11 100 101 110 111 1000 1001 1100 1101 1111 10000 10001 10100 10101

295 3564 75 50 3140 526 28 9 103 6 3 4 4 80 18 17 3

> comb$reason_testing_general_named <- comb$reason_testing_general

> comb$reason_testing_general_named[comb$reason_testing_general == "0"] <- "Unknown"

> comb$reason_testing_general_named[comb$reason_testing_general == "1"] <- "Symptoms"

> comb$reason_testing_general_named[comb$reason_testing_general == "10"] <- "GP"

> comb$reason_testing_general_named[comb$reason_testing_general == "100"] <- "Notification"

> comb$reason_testing_general_named[comb$reason_testing_general == "1000"] <- "Travel"

> comb$reason_testing_general_named[comb$reason_testing_general == "10000"] <- "Other"

> comb$reason_testing_general_named[comb$reason_testing_general == "11"] <- "Symptoms & GP"

> comb$reason_testing_general_named[comb$reason_testing_general == "101"] <- "Notification"

> comb$reason_testing_general_named[comb$reason_testing_general == "110"] <- "Notification & GP"

> comb$reason_testing_general_named[comb$reason_testing_general == "111"] <- "Notification & GP"

> comb$reason_testing_general_named[comb$reason_testing_general == "10001"] <- "Symptoms"

> comb$reason_testing_general_named[comb$reason_testing_general == "1001"] <- "Symptoms & Travel"

> comb$reason_testing_general_named[comb$reason_testing_general == "10010"] <- "GP"

> comb$reason_testing_general_named[comb$reason_testing_general == "11000"] <- "Travel"

> comb$reason_testing_general_named[comb$reason_testing_general == "10011"] <- "Symptoms & GP"

> comb$reason_testing_general_named[comb$reason_testing_general == "10100"] <- "Notification"

> comb$reason_testing_general_named[comb$reason_testing_general == "10101"] <- "Notification"

> comb$reason_testing_general_named[comb$reason_testing_general == "10110"] <- "Notification & GP"

> comb$reason_testing_general_named[comb$reason_testing_general == "1100"] <- "Notification & Travel"

> comb$reason_testing_general_named[comb$reason_testing_general == "1101"] <- "Notification & Travel"

> comb$reason_testing_general_named[comb$reason_testing_general == "1111"] <- "Notification & GP & Travel"

> comb$reason_testing_general_named[comb$reason_testing_general == "11110"] <- "Notification & GP & Travel"

> comb$reason_testing_general_named[comb$reason_testing_general == "11111"] <- "Notification & GP & Travel"

> table(comb$reason_testing_general_named)

GP Notification Notification & GP Notification & GP & Travel Notification & Travel

75 3686 37 4 7

Other Symptoms Symptoms & GP Symptoms & Travel Travel

80 3582 50 6 103

Unknown

295

> #make subset based on exposure notification as then date for exposure is present

> subset_notification<- comb %>%

+ filter(comb$R_allcontact > 1)

> #make new varibale that names the various combinations of notification methods

> subset_notification$notif_method<- subset_notification$W_app + subset_notification$W_GGD + subset_notification$W_huisgenoot + subset_notification$W_pers + subset_notification$W_self #+ subs1$W_anderzz

> subset_notification$notif_method[subset_notification$notif_method == "0"] <- "Unknown"

> subset_notification$notif_method[subset_notification$notif_method == "1"] <- "DCT"

> subset_notification$notif_method[subset_notification$notif_method == "10"] <- "MCT"

> subset_notification$notif_method[subset_notification$notif_method == "100"] <- "Housemate"

> subset_notification$notif_method[subset_notification$notif_method == "1000"] <- "Index"

> subset_notification$notif_method[subset_notification$notif_method == "10000"] <- "Self"

> subset_notification$notif_method[subset_notification$notif_method == "11"] <- "DCT & MCT"

> subset_notification$notif_method[subset_notification$notif_method == "101"] <- "Housemate & DCT"

> subset_notification$notif_method[subset_notification$notif_method == "1001"] <- "DCT & Index"

> subset_notification$notif_method[subset_notification$notif_method == "10001"] <- "DCT"

> subset_notification$notif_method[subset_notification$notif_method == "10010"] <- "MCT"

> subset_notification$notif_method[subset_notification$notif_method == "1010"] <- "MCT & Index"

> subset_notification$notif_method[subset_notification$notif_method == "10100"] <- "Housemate"

> subset_notification$notif_method[subset_notification$notif_method == "10101"] <- "Housemate & DCT"

> subset_notification$notif_method[subset_notification$notif_method == "1011"] <- "DCT & MCT & Index"

> subset_notification$notif_method[subset_notification$notif_method == "110"] <- "Housemate & MCT"

> subset_notification$notif_method[subset_notification$notif_method == "1111"] <- "Housemate & DCT & MCT & Index"

> subset_notification$notif_method[subset_notification$notif_method == "1100"] <- "Housemate & Index"

> subset_notification$notif_method[subset_notification$notif_method == "111"] <- "Housemate & DCT & MCT"

> subset_notification$notif_method[subset_notification$notif_method == "1101"] <- "Housemate & DCT & Index"

> subset_notification$notif_method[subset_notification$notif_method == "1110"] <- "Housemate & MCT & Index"

> subset_notification$notif_method[subset_notification$notif_method == "10110"] <- "Housemate & MCT"

> subset_notification$notif_method[subset_notification$notif_method == "10111"] <- "Housemate & DCT & MCT"

> subset_notification$notif_method[subset_notification$notif_method == "11000"] <- "Index"

> subset_notification$notif_method[subset_notification$notif_method == "11010"] <- "MCT & Index"

> subset_notification$notif_method[subset_notification$notif_method == "11100"] <- "Housemate & Index"

> subset_notification$notif_method[subset_notification$notif_method == "11101"] <- "Housemate & DCT & Index"

> subset_notification$notif_method[subset_notification$notif_method == "11110"] <- "Housemate & MCT & Index"

> subset_notification$notif_method[subset_notification$notif_method == "11111"] <- "Housemate & DCT & MCT & Index"

> table(subset_notification$notif_method)

DCT DCT & Index DCT & MCT & Index Housemate

138 44 10 417

Housemate & DCT Housemate & DCT & Index Housemate & DCT & MCT Housemate & DCT & MCT & Index

18 17 8 13

Housemate & Index Housemate & MCT Housemate & MCT & Index Index

290 52 73 1507

MCT MCT & Index Self Unknown

40 115 236 756

> #group based on hierarchy (so combining groups into 1 reason)

> subset_notification$notif_method_combined<- subset_notification$W_app + subset_notification$W_GGD + subset_notification$W_huisgenoot + subset_notification$W_pers + subset_notification$W_self #+ subs1$W_anderzz

subset_notification$notif_method_combined[subset_notification$notif_method_combined == "0"] <- "Unknown"

>subset_notification$notif_method_combined[subset_notification$notif_method_combined == "1"] <- "DCT"

>subset_notification$notif_method_combined[subset_notification$notif_method_combined == "10"] <- "MCT"

>subset_notification$notif_method_combined[subset_notification$notif_method_combined == "100"] <- "Housemate"

>subset_notification$notif_method_combined[subset_notification$notif_method_combined == "1000"] <- "Index"

>subset_notification$notif_method_combined[subset_notification$notif_method_combined == "10000"] <- "Self"

>subset_notification$notif_method_combined[subset_notification$notif_method_combined == "11"] <- "DCT"

>subset_notification$notif_method_combined[subset_notification$notif_method_combined == "101"] <- "Housemate"

>subset_notification$notif_method_combined[subset_notification$notif_method_combined == "1001"] <- "DCT"

>subset_notification$notif_method_combined[subset_notification$notif_method_combined == "10001"] <- "DCT"

>subset_notification$notif_method_combined[subset_notification$notif_method_combined == "10010"] <- "MCT"

>subset_notification$notif_method_combined[subset_notification$notif_method_combined == "1010"] <- "MCT"

>subset_notification$notif_method_combined[subset_notification$notif_method_combined == "10100"] <- "Housemate"

>subset_notification$notif_method_combined[subset_notification$notif_method_combined == "10101"] <- "Housemate"

>subset_notification$notif_method_combined[subset_notification$notif_method_combined == "1011"] <- "DCT"

>subset_notification$notif_method_combined[subset_notification$notif_method_combined == "110"] <- "Housemate"

>subset_notification$notif_method_combined[subset_notification$notif_method_combined == "1111"] <- "Housemate"

>subset_notification$notif_method_combined[subset_notification$notif_method_combined == "1100"] <- "Housemate"

>subset_notification$notif_method_combined[subset_notification$notif_method_combined == "111"] <- "Housemate"

>subset_notification$notif_method_combined[subset_notification$notif_method_combined == "1101"] <- "Housemate"

>subset_notification$notif_method_combined[subset_notification$notif_method_combined == "1110"] <- "Housemate"

>subset_notification$notif_method_combined[subset_notification$notif_method_combined == "10110"] <- "Housemate"

>subset_notification$notif_method_combined[subset_notification$notif_method_combined == "10111"] <- "Housemate"

>subset_notification$notif_method_combined[subset_notification$notif_method_combined == "11000"] <- "Index"

>subset_notification$notif_method_combined[subset_notification$notif_method_combined == "11010"] <- "MCT"

>subset_notification$notif_method_combined[subset_notification$notif_method_combined == "11100"] <- "Housemate"

>subset_notification$notif_method_combined[subset_notification$notif_method_combined == "11101"] <- "Housemate"

>subset_notification$notif_method_combined[subset_notification$notif_method_combined == "11110"] <- "Housemate"

>subset_notification$notif_method_combined[subset_notification$notif_method_combined == "11111"] <- "Housemate"

> table(subset_notification$notif_method_combined)

DCT Housemate Index MCT Self Unknown

192 888 1507 155 236 756

> #add variable for combined reason for testing

> dataset_notification_id<- subset_notification %>%

+ select(notif_method_combined, id)

> dataset_comb_reason_testing<- left_join(comb, dataset_notification_id, by= "id" )

> dataset_comb_reason_testing$comb_reason_testing <-

+ ifelse(dataset_comb_reason_testing$reason_testing_general_named== "GP", 'Other',

+ ifelse(dataset_comb_reason_testing$reason_testing_general_named== "Other", 'Other',

+ ifelse(dataset_comb_reason_testing$reason_testing_general_named== "Symptoms", 'Symptoms',

+ ifelse(dataset_comb_reason_testing$reason_testing_general_named== "Symptoms & GP", 'Other',

+ ifelse(dataset_comb_reason_testing$reason_testing_general_named== "Symptoms & Travel", 'Other',

+ ifelse(dataset_comb_reason_testing$reason_testing_general_named== "Travel", 'Other',

+ ifelse(dataset_comb_reason_testing$reason_testing_general_named== "Unknown", 'Unknown reason',

+ ifelse(dataset_comb_reason_testing$notif_method_combined== "MCT", 'MCT',

+ ifelse(dataset_comb_reason_testing$notif_method_combined== "DCT", 'DCT',

+ ifelse(dataset_comb_reason_testing$notif_method_combined== "Housemate", 'Housemate',

+ ifelse(dataset_comb_reason_testing$notif_method_combined== "Index", 'Index',

+ ifelse(dataset_comb_reason_testing$notif_method_combined== "Other", 'Unknown notification',

+ ifelse(dataset_comb_reason_testing$notif_method_combined== "Self", 'Self',

+ ifelse(dataset_comb_reason_testing$notif_method_combined== "Unknown", 'Unknown notification', NA))))))))))))))

> table(dataset_comb_reason_testing$comb_reason_testing)

DCT Housemate Index MCT Other Self

192 888 1507 155 314 236

Symptoms Unknown notification Unknown reason

3582 756 295

> #make variable for date of testing a datatype date

> dataset_comb_reason_testing <- dataset_comb_reason_testing %>%

+ mutate(Afname_dt = lubridate:: mdy(Afname_dt))

> dataset_comb_reason_testing$Afname_dt[dataset_comb_reason_testing$Afname_dt == "2021-01-21"] <- '2021-04-21'

> #graph of reason for testing over time

> grouped_reason_testing<- dataset_comb_reason_testing %>%

+ group_by(comb_reason_testing, Afname_dt) %>%

+ summarise(uniqueid= n_distinct(id))

`summarise()` has grouped output by 'comb_reason_testing'. You can override using the `.groups` argument.

> grouped_reason_testing<- transform(grouped_reason_testing, percent= ave(uniqueid, Afname_dt, FUN= prop.table))

> grouped_reason_testing$percent <- grouped_reason_testing$percent * 100

> ggplot(data = grouped_reason_testing, aes(x= Afname_dt, y=percent, color= fct_relevel(comb_reason_testing, "DCT", "MCT", "Index", "Housemate", "Self","Unknown notification","Symptoms","Other", "Unknown reason"))) +

+ geom_line(size=1)+

+ scale_x_date(date_breaks = "2 week", date_labels = "%d %b. %Y") +

+ ylim(0, 100)+

+ scale_color_manual(values = c("deeppink3", "darkblue", "darkgreen", "lightgreen", "yellow","lightblue", "orange", "lightpink", "dimgray" )) +

+ labs(y= "Percentage of total tests", x= "Testing date", color= "Reason for testing") +

+ theme(panel.background = element_rect(fill = "white"),

+ panel.grid.major = element_line(color = "lightgray", linetype = "dashed"),

+ axis.line = element_line(color = "white", size = 2),

+ legend.key = element_rect(fill = "white", size = 10),

+ legend.background = element_rect(fill = "white"),

+ legend.text = element_text(size = 14, color = "black"),

+ legend.title = element_text(size = 14, face = "bold"),

+ axis.text.x = element_text(size = 12, color = "black"),

+ axis.text.y = element_text(size = 12, color = "black"),

+ axis.title.x = element_text(size = 14, face = "bold"),

+ axis.title.y = element_text(size = 14, face = "bold"))

> #recode variables

> dataset_comb_reason_testing$Uitslag_PCR[dataset_comb_reason_testing$Uitslag_PCR == "0"] <- "Negative"

> dataset_comb_reason_testing$Uitslag_PCR[dataset_comb_reason_testing$Uitslag_PCR == "1"] <- "Positive"

> dataset_comb_reason_testing$agegrp<-cut(dataset_comb_reason_testing$Leeftijd, c(15,29,44,59,100),

+ labels=c("16-29", "30-44", "45-59", "60+") )

> dataset_comb_reason_testing$location<- dataset_comb_reason_testing$Antigeen_sneltest

> dataset_comb_reason_testing$Geslacht[dataset_comb_reason_testing$Geslacht== ""]<- NA

> dataset_comb_reason_testing$location[dataset_comb_reason_testing$location == "BD-veritor"] <- "West-Brabant"

> dataset_comb_reason_testing$location[dataset_comb_reason_testing$location == "Roche"] <- "Rotterdam-Rijnmond"

> dataset_comb_reason_testing$location[dataset_comb_reason_testing$location == "Abbott"] <- "IJsselland"

dataset_comb_reason_testing$Gevaccineerd[dataset_comb_reason_testing$Gevaccineerd== " "]<- NA

dataset_comb_reason_testing$Eerdercorona[dataset_comb_reason_testing$Eerdercorona== " "]<- NA

> dataset_comb_reason_testing$status<- 1

> dataset_comb_reason_testing$R_klachten<- as.numeric(dataset_comb_reason_testing$R_klachten)

> dataset_comb_reason_testing$Klachten<- replace_na(dataset_comb_reason_testing$Klachten, 100)

> dataset_comb_reason_testing$Klachten<- as.numeric(dataset_comb_reason_testing$Klachten)

> dataset_comb_reason_testing$Klachtenzz<- dataset_comb_reason_testing$R_klachten + dataset_comb_reason_testing$Klachten

> dataset_comb_reason_testing$Klachtenzz[dataset_comb_reason_testing$Klachtenzz == "0"] <- "0"

> dataset_comb_reason_testing$Klachtenzz[dataset_comb_reason_testing$Klachtenzz == "1"] <- "1"

> dataset_comb_reason_testing$Klachtenzz[dataset_comb_reason_testing$Klachtenzz == "2"] <- "1"

> dataset_comb_reason_testing$Klachtenzz[dataset_comb_reason_testing$Klachtenzz == "100"] <- NA

> dataset_comb_reason_testing$Klachtenzz[dataset_comb_reason_testing$Klachtenzz == "101"] <- "1"

> dataset_comb_reason_testing$int_symp_test<- dataset_comb_reason_testing$Kl_sinds

dataset_comb_reason_testing$int_symp_test[dataset_comb_reason_testing$int_symp_test == "vandaag"] <- "0"

dataset_comb_reason_testing$int_symp_test[dataset_comb_reason_testing$int_symp_test == "gisteren"] <- "1"

dataset_comb_reason_testing$int_symp_test[dataset_comb_reason_testing$int_symp_test == "eergisteren"] <- "2"

dataset_comb_reason_testing$int_symp_test[dataset_comb_reason_testing$int_symp_test == "????? 3 dagen"] <- "3"

dataset_comb_reason_testing$int_symp_test[dataset_comb_reason_testing$int_symp_test == "Onbekend"] <- NA

> dataset_comb_reason_testing$int_symp_test<- as.numeric(dataset_comb_reason_testing$int_symp_test)

> dataset_comb_reason_testing$Uitslag_PCR<- as.factor(dataset_comb_reason_testing$Uitslag_PCR)

> #mean interval calculations

> subset_comb_reason_testing<- dataset_comb_reason_testing %>%

+ filter(dataset_comb_reason_testing$R_allcontact > 1)

> subset_comb_reason_testing$dt_lcontact[subset_comb_reason_testing$dt_lcontact == " "] <- NA

> subset_comb_reason_testing <- subset_comb_reason_testing %>%

+ mutate(dt_lcontact = lubridate:: mdy(dt_lcontact))

> subset_comb_reason_testing$int_exp_test<- difftime(subset_comb_reason_testing$Afname_dt, subset_comb_reason_testing$dt_lcontact, units = "days")

> subset_comb_reason_testing$int_exp_test<- as.numeric(subset_comb_reason_testing$int_exp_test)

> #if interval less than 0 or more than 14 then set to NA

> subset_comb_reason_testing<- subset_comb_reason_testing %>%

+ mutate(int_exp_test = replace(int_exp_test, int_exp_test< 0, NA))

> subset_comb_reason_testing<- subset_comb_reason_testing %>%

+ mutate(int_exp_test = replace(int_exp_test, int_exp_test>14, NA))

> subset_compint<- subset_comb_reason_testing %>%

+ filter(int_exp_test >= 0)

> #check number of missings per variable

> sum(is.na(subset_comb_reason_testing$Klachtenzz))

[1] 16

> sum(is.na(subset_comb_reason_testing$Uitslag_PCR))

[1] 0

> sum(is.na(subset_comb_reason_testing$Leeftijd))

[1] 8

> sum(is.na(subset_comb_reason_testing$Geslacht))

[1] 11

> sum(is.na(subset_comb_reason_testing$location))

[1] 0

> sum(is.na(subset_comb_reason_testing$Gevaccineerd))

[1] 3

> sum(is.na(subset_comb_reason_testing$Eerdercorona))

[1] 18

> #make summary table for table 1 on grouping

> summary_table <- dataset_comb_reason_testing %>%

+ group_by(exp_notif) %>%

+ summarise(

+ cases = n(),

+ prc_case = scales::percent(cases/7925 , accuracy = 0.01L),

+ all = round(mean(int_exp_test, na.rm = T), digits=2),

+ all_sd = round(sd(int_exp_test, na.rm = T), digits=2), )

> #stat tests

> chisq.test(dataset_comb_reason_testing$comb_reason_testing, dataset_comb_reason_testing$Klachtenzz)

Pearson's Chi-squared test

data: dataset_comb_reason_testing$comb_reason_testing and dataset_comb_reason_testing$Klachtenzz

X-squared = 3828.8, df = 8, p-value < 2.2e-16

> chisq.test(dataset_comb_reason_testing$comb_reason_testing, dataset_comb_reason_testing$Uitslag_PCR)

Pearson's Chi-squared test

data: dataset_comb_reason_testing$comb_reason_testing and dataset_comb_reason_testing$Uitslag_PCR

X-squared = 242.92, df = 8, p-value < 2.2e-16

> ct.aov <- aov(Ct ~ comb_reason_testing, data = subset_pos)

> summary(ct.aov)

Df Sum Sq Mean Sq F value Pr(>F)

comb_reason_testing 8 997 124.69 4.28 4.79e-05 ***

Residuals 738 21498 29.13

---

Signif. codes: 0 ‘***’ 0.001 ‘**’ 0.01 ‘*’ 0.05 ‘.’ 0.1 ‘ ’ 1

26 observations deleted due to missingness

> kruskal.test(Ct~ comb_reason_testing, data =subset_pos)

Kruskal-Wallis rank sum test

data: Ct by comb_reason_testing

Kruskal-Wallis chi-squared = 30.564, df = 8, p-value = 0.0001679

> kruskal.test(Leeftijd ~ comb_reason_testing, data = dataset_comb_reason_testing)

Kruskal-Wallis rank sum test

data: Leeftijd by comb_reason_testing

Kruskal-Wallis chi-squared = 198.86, df = 8, p-value < 2.2e-16

> age.aov <- aov(Leeftijd ~ comb_reason_testing, data = dataset_comb_reason_testing)

> summary(age.aov)

Df Sum Sq Mean Sq F value Pr(>F)

comb_reason_testing 8 48950 6119 26.76 <2e-16 ***

Residuals 7897 1805623 229

---

Signif. codes: 0 ‘***’ 0.001 ‘**’ 0.01 ‘*’ 0.05 ‘.’ 0.1 ‘ ’ 1

19 observations deleted due to missingness

> chisq.test(dataset_comb_reason_testing$comb_reason_testing, dataset_comb_reason_testing$Geslacht)

Pearson's Chi-squared test

data: dataset_comb_reason_testing$comb_reason_testing and dataset_comb_reason_testing$Geslacht

X-squared = 21.872, df = 8, p-value = 0.005158

> chisq.test(dataset_comb_reason_testing$comb_reason_testing, dataset_comb_reason_testing$location)

Pearson's Chi-squared test

data: dataset_comb_reason_testing$comb_reason_testing and dataset_comb_reason_testing$location

X-squared = 612.98, df = 16, p-value < 2.2e-16

> chisq.test(dataset_comb_reason_testing$comb_reason_testing, dataset_comb_reason_testing$Gevaccineerd)

Pearson's Chi-squared test

data: dataset_comb_reason_testing$comb_reason_testing and dataset_comb_reason_testing$Gevaccineerd

X-squared = 122.41, df = 8, p-value < 2.2e-16

> chisq.test(dataset_comb_reason_testing$comb_reason_testing, dataset_comb_reason_testing$Eerdercorona)

Pearson's Chi-squared test

data: dataset_comb_reason_testing$comb_reason_testing and dataset_comb_reason_testing$Eerdercorona

X-squared = 15.197, df = 8, p-value = 0.05542

> #ad-hoc analyses

> sig<- .05

> sig_test<- table(dataset_comb_reason_testing$comb_reason_testing, dataset_comb_reason_testing$Uitslag_PCR)

> sigadj<-sig/(nrow(sig_test)*ncol(sig_test))

> qnorm(sigadj/2)

[1] -2.991316

> chisq.test(sig_test, correct= FALSE)$stdres

Negative Positive

DCT 2.88787810 -2.88787810

Housemate -13.96986149 13.96986149

Index 0.67458339 -0.67458339

MCT 1.12608177 -1.12608177

Other 2.83907141 -2.83907141

Self 0.44978670 -0.44978670

Symptoms 8.24540279 -8.24540279

Unknown notification -4.02899587 4.02899587

Unknown reason -0.04517268 0.04517268

> sig_age <- pairwise.t.test(dataset_comb_reason_testing$Leeftijd, dataset_comb_reason_testing$comb_reason_testing,

+ p.adj = "bonferroni", pool.sd = FALSE)

> print(sig_age$p.value < 0.05)

DCT Housemate Index MCT Other Self Symptoms Unknown notification

Housemate TRUE NA NA NA NA NA NA NA

Index TRUE FALSE NA NA NA NA NA NA

MCT FALSE FALSE FALSE NA NA NA NA NA

Other TRUE TRUE TRUE TRUE NA NA NA NA

Self FALSE TRUE TRUE FALSE TRUE NA NA NA

Symptoms FALSE TRUE TRUE FALSE TRUE FALSE NA NA

Unknown notification FALSE TRUE TRUE FALSE TRUE FALSE FALSE NA

Unknown reason FALSE TRUE TRUE TRUE FALSE TRUE TRUE TRUE

> sig_ct <- pairwise.t.test(dataset_comb_reason_testing$Ct, dataset_comb_reason_testing$comb_reason_testing,

+ p.adj = "bonferroni", pool.sd = FALSE)

> print(sig_ct$p.value < 0.05)

DCT Housemate Index MCT Other Self Symptoms Unknown notification

Housemate FALSE NA NA NA NA NA NA NA

Index FALSE FALSE NA NA NA NA NA NA

MCT FALSE FALSE FALSE NA NA NA NA NA

Other FALSE FALSE FALSE FALSE NA NA NA NA

Self FALSE FALSE FALSE FALSE FALSE NA NA NA

Symptoms FALSE FALSE TRUE FALSE FALSE FALSE NA NA

Unknown notification FALSE FALSE FALSE FALSE FALSE FALSE FALSE NA

Unknown reason FALSE FALSE FALSE FALSE FALSE FALSE FALSE FALSE

> sig_gender<- table(dataset_comb_reason_testing$comb_reason_testing, dataset_comb_reason_testing$Geslacht)

> sigadj<-sig/(nrow(sig_gender)*ncol(sig_gender))

> qnorm(sigadj/2)

[1] -2.991316

> chisq.test(sig_gender, correct= FALSE)$stdres

Female Male

DCT 0.3694169 -0.3694169

Housemate 0.5865229 -0.5865229

Index -0.7781620 0.7781620

MCT 0.8427012 -0.8427012

Other -2.6234326 2.6234326

Self 1.2789471 -1.2789471

Symptoms 2.5773335 -2.5773335

Unknown notification -2.8228162 2.8228162

Unknown reason -1.1175617 1.1175617

> sig_location<- table(dataset_comb_reason_testing$comb_reason_testing, dataset_comb_reason_testing$location)

> sigadj<-sig/(nrow(sig_location)*ncol(sig_location))

> qnorm(sigadj/2)

[1] -3.113017

> chisq.test(sig_location, correct= FALSE)$stdres

IJsselland Rotterdam-Rijnmond West-Brabant

DCT -3.0543843 0.6220602 3.0474875

Housemate -7.5547578 3.7002733 4.7582767

Index -8.4499812 1.9298242 8.1623151

MCT 6.9820193 -3.7020349 -4.0345869

Other 1.3163238 -1.1624365 -0.1634105

Self -1.9195620 -1.7291866 4.6412468

Symptoms 17.6670308 -4.5043765 -16.4618550

Unknown notification -12.2519874 5.1758518 8.7776641

Unknown reason 0.4104019 -1.4023340 1.2861497

> sig_vac<- table(dataset_comb_reason_testing$comb_reason_testing, dataset_comb_reason_testing$Gevaccineerd)

> sigadj<-sig/(nrow(sig_vac)*ncol(sig_vac))

> qnorm(sigadj/2)

[1] -2.991316

> chisq.test(sig_vac, correct= FALSE)$stdres

ja nee

DCT 2.3859175 -2.3859175

Housemate -1.8217100 1.8217100

Index -4.6688949 4.6688949

MCT 4.2622439 -4.2622439

Other 5.4383103 -5.4383103

Self -0.7446034 0.7446034

Symptoms -1.4614618 1.4614618

Unknown notification 1.4361629 -1.4361629

Unknown reason 6.8792931 -6.8792931

> sig_prior<- table(dataset_comb_reason_testing$comb_reason_testing, dataset_comb_reason_testing$Eerdercorona)

> sigadj<-sig/(nrow(sig_prior)*ncol(sig_prior))

> qnorm(sigadj/2)

[1] -2.991316

> chisq.test(sig_prior, correct= FALSE)$stdres

ja nee

DCT -0.98825914 0.98825914

Housemate -0.55269082 0.55269082

Index 1.86099959 -1.86099959

MCT 1.55503660 -1.55503660

Other 1.56617744 -1.56617744

Self -1.77865842 1.77865842

Symptoms -1.63491761 1.63491761

Unknown notification 0.06247611 -0.06247611

Unknown reason 1.40933182 -1.40933182

> #mean interval calculations for table 3

> subset_compint$Klachtenzz[subset_compint$Klachtenzz == "0"] <- "No"

> subset_compint$Klachtenzz[subset_compint$Klachtenzz == "1"] <- "Yes"

> summary_table <- subset_compint %>%

+ group_by(exp_notif) %>%

+ summarise(

+ cases = n(),

+ prc_case = scales::percent(cases/3172 , accuracy = 0.01L),

+ all = round(mean(int_exp_test, na.rm = T), digits=2),

+ all_sd = round(sd(int_exp_test, na.rm = T), digits=2),

+ symp_no = round(mean(int_exp_test [Klachtenzz== "No"], na.rm = T), digits = 2),

+ symp_no_sd = round(sd(int_exp_test [Klachtenzz== "No"], na.rm = T), digits = 2),

+ symp_yes = round(mean(int_exp_test [Klachtenzz== "Yes"], na.rm = T), digits = 2),

+ symp_yes_sd = round(sd(int_exp_test [Klachtenzz== "Yes"], na.rm = T), digits = 2),

+ neg_test= round(mean(int_exp_test [Uitslag_PCR == "Negative"], na.rm = T), digits = 2),

+ neg_sd= round(sd(int_exp_test [Uitslag_PCR == "Negative"], na.rm = T), digits = 2),

+ pos_test= round(mean(int_exp_test [Uitslag_PCR == "Positive"], na.rm = T), digits = 2),

+ pos_sd= round(sd(int_exp_test [Uitslag_PCR == "Positive"], na.rm = T), digits = 2),

+ age_1 = round(mean(int_exp_test [agegrp == "16-29"], na.rm = T), digits = 2),

+ age_1_sd = round(sd(int_exp_test [agegrp == "16-29"], na.rm = T), digits = 2),

+ age_2 = round(mean(int_exp_test [agegrp == "30-44"], na.rm = T), digits = 2),

+ age_2_sd = round(sd(int_exp_test [agegrp == "30-44"], na.rm = T), digits = 2),

+ age_3 = round(mean(int_exp_test [agegrp == "45-59"], na.rm = T), digits = 2),

+ age_3_sd = round(sd(int_exp_test [agegrp == "45-59"], na.rm = T), digits = 2),

+ age_4 = round(mean(int_exp_test [agegrp == "60+"], na.rm = T), digits = 2),

+ age_4_sd = round(sd(int_exp_test [agegrp == "60+"], na.rm = T), digits = 2),

+ female = round(mean(int_exp_test [Geslacht == "Female"], na.rm = T), digits = 2),

+ female_sd= round(sd(int_exp_test [Geslacht == "Female"], na.rm = T), digits = 2),

+ male= round(mean(int_exp_test [Geslacht == "Male"], na.rm = T), digits = 2),

+ male_sd= round(sd(int_exp_test [Geslacht == "Male"], na.rm = T), digits = 2),

+ BRA = round(mean(int_exp_test [location == "West-Brabant"], na.rm = T), digits = 2),

+ BRA_sd = round(sd(int_exp_test [location == "West-Brabant"], na.rm = T), digits = 2),

+ ROT = round(mean(int_exp_test [location == "Rotterdam-Rijnmond"], na.rm = T), digits = 2),

+ ROT_sd = round(sd(int_exp_test [location == "Rotterdam-Rijnmond"], na.rm = T), digits = 2),

+ ZWOL = round(mean(int_exp_test [location == "IJsselland"], na.rm = T), digits = 2),

+ ZWOL_sd = round(sd(int_exp_test [location == "IJsselland"], na.rm = T), digits = 2),

+ vac_no = round(mean(int_exp_test [Gevaccineerd== "nee"], na.rm = T), digits = 2),

+ vac_no_sd = round(sd(int_exp_test [Gevaccineerd== "nee"], na.rm = T), digits = 2),

+ vac_yes = round(mean(int_exp_test [Gevaccineerd== "ja"], na.rm = T), digits = 2),

+ vac_yes_sd = round(sd(int_exp_test [Gevaccineerd== "ja"], na.rm = T), digits = 2),

+ prior_no = round(mean(int_exp_test [Eerdercorona == "nee" ], na.rm = T), digits = 2),

+ prior_no_sd = round(sd(int_exp_test [Eerdercorona == "nee" ], na.rm = T), digits = 2),

+ prior_yes = round(mean(int_exp_test [Eerdercorona == "ja" ], na.rm = T), digits = 2),

+ prior_yes_sd = round(sd(int_exp_test [Eerdercorona == "ja" ], na.rm = T), digits = 2),)

> #num missings

> sum(is.na(subset_compint$Klachtenzz))

[1] 13

> sum(is.na(subset_compint$Uitslag_PCR))

[1] 0

> sum(is.na(subset_compint$Leeftijd))

[1] 6

> sum(is.na(subset_compint$Geslacht))

[1] 9

> sum(is.na(subset_compint$location))

[1] 0

> sum(is.na(subset_compint$Gevaccineerd))

[1] 1

> sum(is.na(subset_compint$Eerdercorona))

[1] 9

> #releveling data to set reference group

> subset_compint$W_app[subset_compint$W_app == "0"] <- "No"

> subset_compint$W_app[subset_compint$W_app == "1"] <- "Yes"

> subset_compint$W_GGD[subset_compint$W_GGD == "0"] <- "No"

> subset_compint$W_GGD[subset_compint$W_GGD == "10"] <- "Yes"

> subset_compint$W_huisgenoot[subset_compint$W_huisgenoot == "0"] <- "No"

> subset_compint$W_huisgenoot[subset_compint$W_huisgenoot == "100"] <- "Yes"

> subset_compint$W_pers[subset_compint$W_pers == "0"] <- "No"

> subset_compint$W_pers[subset_compint$W_pers == "1000"] <- "Yes"

> subset_compint$W_self [subset_compint$W_self == "0"] <- "No"

> subset_compint$W_self[subset_compint$W_self == "100000"] <- "Yes"

> subset_compint$W_unknown<- ifelse(subset_compint$comb_reason_testing=="Unknown notification", "Yes", "No")

> subset_compint$comb_reason_testing <- as.factor(subset_compint$comb_reason_testing)

> subset_compint$comb_reason_testing <- relevel(subset_compint$comb_reason_testing, ref = 2)

> subset_compint$location <- as.factor(subset_compint$location)

> subset_compint$location <- relevel(subset_compint$location, ref = 3)

> subset_compint$Gevaccineerd <- as.factor(subset_compint$Gevaccineerd)

> subset_compint$Gevaccineerd<- relevel(subset_compint$Gevaccineerd, ref=2)

> subset_compint$Eerdercorona <- as.factor(subset_compint$Eerdercorona)

> subset_compint$Eerdercorona<- relevel(subset_compint$Eerdercorona, ref=2)

> subset_compint$int_exp_test_plus1<- subset_compint$int_exp_test + 1

> #Univariate Weibull regression model

> occ<-survreg(Surv(int_exp_test_plus1, status)~ W_app,

+ data= subset_compint, dist='weibull')

> summary(occ)

Call:

survreg(formula = Surv(int_exp_test_plus1, status) ~ W_app, data = subset_compint,

dist = "weibull")

Value Std. Error z p

(Intercept) 1.77323 0.00678 261.63 <2e-16

W_appYes 0.06883 0.02574 2.67 0.0075

Log(scale) -1.03387 0.01418 -72.91 <2e-16

Scale= 0.356

Weibull distribution

Loglik(model)= -6825 Loglik(intercept only)= -6828.7

Chisq= 7.54 on 1 degrees of freedom, p= 0.006

Number of Newton-Raphson Iterations: 7

n= 3171

> ConvertWeibull(occ, conf.level = 0.95)

$vars

Estimate SE

lambda 0.006831744 0.0005329707

gamma 2.811923384 0.0398751238

W_appYes -0.193545621 0.0724864349

$HR

HR LB UB

W_appYes 0.8240322 0.7148973 0.9498276

$ETR

ETR LB UB

W_appYes 1.071254 1.018543 1.126694

> occ<-survreg(Surv(int_exp_test_plus1, status)~ W_GGD,

+ data= subset_compint, dist='weibull')

> summary(occ)

Call:

survreg(formula = Surv(int_exp_test_plus1, status) ~ W_GGD, data = subset_compint,

dist = "weibull")

Value Std. Error z p

(Intercept) 1.77312 0.00687 258.08 <2e-16

W_GGDYes 0.05037 0.02196 2.29 0.022

Log(scale) -1.03338 0.01417 -72.92 <2e-16

Scale= 0.356

Weibull distribution

Loglik(model)= -6826 Loglik(intercept only)= -6828.7

Chisq= 5.46 on 1 degrees of freedom, p= 0.02

Number of Newton-Raphson Iterations: 7

n= 3171

> ConvertWeibull(occ, conf.level = 0.95)

$vars

Estimate SE

lambda 0.006850468 0.0005341121

gamma 2.810552127 0.0398285996

W_GGDYes -0.141554184 0.0617806254

$HR

HR LB UB

W_GGDYes 0.8680081 0.7690172 0.9797416

$ETR

ETR LB UB

W_GGDYes 1.051655 1.007357 1.097902

> occ<-survreg(Surv(int_exp_test_plus1, status)~ W_pers,

+ data= subset_compint, dist='weibull')

> summary(occ)

Call:

survreg(formula = Surv(int_exp_test_plus1, status) ~ W_pers,

data = subset_compint, dist = "weibull")

Value Std. Error z p

(Intercept) 1.7620 0.0105 167.77 <2e-16

W_persYes 0.0251 0.0131 1.92 0.055

Log(scale) -1.0309 0.0142 -72.84 <2e-16

Scale= 0.357

Weibull distribution

Loglik(model)= -6826.9 Loglik(intercept only)= -6828.7

Chisq= 3.68 on 1 degrees of freedom, p= 0.055

Number of Newton-Raphson Iterations: 7

n= 3171

> ConvertWeibull(occ, conf.level = 0.95)

$vars

Estimate SE

lambda 0.007154129 0.0005835611

gamma 2.803699588 0.0396795696

W_persYes -0.070338293 0.0365636868

$HR

HR LB UB

W_persYes 0.9320785 0.8676197 1.001326

$ETR

ETR LB UB

W_persYes 1.025405 0.9994996 1.051982

> occ<-survreg(Surv(int_exp_test_plus1, status)~ W_huisgenoot,

+ data= subset_compint, dist='weibull')

> summary(occ)

Call:

survreg(formula = Surv(int_exp_test_plus1, status) ~ W_huisgenoot,

data = subset_compint, dist = "weibull")

Value Std. Error z p

(Intercept) 1.80902 0.00745 242.76 <2e-16

W_huisgenootYes -0.14633 0.01480 -9.89 <2e-16

Log(scale) -1.02968 0.01407 -73.21 <2e-16

Scale= 0.357

Weibull distribution

Loglik(model)= -6782.2 Loglik(intercept only)= -6828.7

Chisq= 93.16 on 1 degrees of freedom, p= 4.8e-22

Number of Newton-Raphson Iterations: 8

n= 3171

> ConvertWeibull(occ, conf.level = 0.95)

$vars

Estimate SE

lambda 0.006310478 0.000490926

gamma 2.800164783 0.039385616

W_huisgenootYes 0.409759280 0.040960162

$HR

HR LB UB

W_huisgenootYes 1.506455 1.390243 1.632381

$ETR

ETR LB UB

W_huisgenootYes 0.8638691 0.8391649 0.8893006

> occ<-survreg(Surv(int_exp_test_plus1, status)~ W_self,

+ data= subset_compint, dist='weibull')

> summary(occ)

Call:

survreg(formula = Surv(int_exp_test_plus1, status) ~ W_self,

data = subset_compint, dist = "weibull")

Value Std. Error z p

(Intercept) 1.8264 0.0268 68.04 <2e-16

W_selfNo -0.0516 0.0275 -1.87 0.061

Log(scale) -1.0327 0.0142 -72.94 <2e-16

Scale= 0.356

Weibull distribution

Loglik(model)= -6826.9 Loglik(intercept only)= -6828.7

Chisq= 3.66 on 1 degrees of freedom, p= 0.056

Number of Newton-Raphson Iterations: 7

n= 3171

> ConvertWeibull(occ, conf.level = 0.95)

$vars

Estimate SE

lambda 0.005918271 0.0006434232

gamma 2.808603344 0.0397623118

W_selfNo 0.144894354 0.0774062823

$HR

HR LB UB

W_selfNo 1.155917 0.993204 1.345288

$ETR

ETR LB UB

W_selfNo 0.9497187 0.8998083 1.002397

> occ<-survreg(Surv(int_exp_test_plus1, status)~ W_unknown,

+ data= subset_compint, dist='weibull')

> summary(occ)

Call:

survreg(formula = Surv(int_exp_test_plus1, status) ~ W_unknown,

data = subset_compint, dist = "weibull")

Value Std. Error z p

(Intercept) 1.77503 0.00717 247.58 <2e-16

W_unknownYes 0.01670 0.01723 0.97 0.33

Log(scale) -1.03155 0.01415 -72.92 <2e-16

Scale= 0.356

Weibull distribution

Loglik(model)= -6828.3 Loglik(intercept only)= -6828.7

Chisq= 0.95 on 1 degrees of freedom, p= 0.33

Number of Newton-Raphson Iterations: 7

n= 3171

> ConvertWeibull(occ, conf.level = 0.95)

$vars

Estimate SE

lambda 0.006876149 0.0005379969

gamma 2.805408088 0.0396840730

W_unknownYes -0.046861776 0.0483395916

$HR

HR LB UB

W_unknownYes 0.9542193 0.8679635 1.049047

$ETR

ETR LB UB

W_unknownYes 1.016844 0.9830705 1.051779

> occ<-survreg(Surv(int_exp_test_plus1, status)~ Klachtenzz,

+ data= subset_compint, dist='weibull')

> summary(occ)

Call:

survreg(formula = Surv(int_exp_test_plus1, status) ~ Klachtenzz,

data = subset_compint, dist = "weibull")

Value Std. Error z p

(Intercept) 1.7854 0.0078 228.95 <2e-16

KlachtenzzYes -0.0249 0.0138 -1.81 0.07

Log(scale) -1.0305 0.0142 -72.67 <2e-16

Scale= 0.357

Weibull distribution

Loglik(model)= -6799.6 Loglik(intercept only)= -6801.2

Chisq= 3.26 on 1 degrees of freedom, p= 0.071

Number of Newton-Raphson Iterations: 7

n=3158 (13 observations deleted due to missingness)

> ConvertWeibull(occ, conf.level = 0.95)

$vars

Estimate SE

lambda 0.00671433 0.0005253168

gamma 2.80241429 0.0397376342

KlachtenzzYes 0.06982463 0.0385176420

$HR

HR LB UB

KlachtenzzYes 1.07232 0.9943475 1.156407

$ETR

ETR LB UB

KlachtenzzYes 0.975392 0.9494242 1.00207

> occ<-survreg(Surv(int_exp_test_plus1, status)~ Uitslag_PCR,

+ data= subset_compint, dist='weibull')

> summary(occ)

Call:

survreg(formula = Surv(int_exp_test_plus1, status) ~ Uitslag_PCR,

data = subset_compint, dist = "weibull")

Value Std. Error z p

(Intercept) 1.78674 0.00699 255.51 < 2e-16

Uitslag_PCRPositive -0.07764 0.01897 -4.09 4.3e-05

Log(scale) -1.03039 0.01412 -72.97 < 2e-16

Scale= 0.357

Weibull distribution

Loglik(model)= -6820.8 Loglik(intercept only)= -6828.7

Chisq= 15.97 on 1 degrees of freedom, p= 6.4e-05

Number of Newton-Raphson Iterations: 7

n= 3171

> ConvertWeibull(occ, conf.level = 0.95)

$vars

Estimate SE

lambda 0.006692739 0.0005203114

gamma 2.802163168 0.0395686345

Uitslag_PCRPositive 0.217558192 0.0529961720

$HR

HR LB UB

Uitslag_PCRPositive 1.243038 1.120402 1.379097

$ETR

ETR LB UB

Uitslag_PCRPositive 0.9252981 0.8915266 0.9603487

> occ<-survreg(Surv(int_exp_test_plus1, status)~ agegrp,

+ data= subset_compint, dist='weibull')

> summary(occ)

Call:

survreg(formula = Surv(int_exp_test_plus1, status) ~ agegrp,

data = subset_compint, dist = "weibull")

Value Std. Error z p

(Intercept) 1.76684 0.00949 186.24 <2e-16

agegrp30-44 0.02854 0.01591 1.79 0.073

agegrp45-59 0.00612 0.01674 0.37 0.715

agegrp60+ 0.03004 0.02298 1.31 0.191

Log(scale) -1.03249 0.01420 -72.70 <2e-16

Scale= 0.356

Weibull distribution

Loglik(model)= -6815.8 Loglik(intercept only)= -6817.9

Chisq= 4.21 on 3 degrees of freedom, p= 0.24

Number of Newton-Raphson Iterations: 7

n=3165 (6 observations deleted due to missingness)

> ConvertWeibull(occ, conf.level = 0.95)

$vars

Estimate SE

lambda 0.007003096 0.0005561081

gamma 2.808061385 0.0398825235

agegrp30-44 -0.080145843 0.0447390084

agegrp45-59 -0.017193492 0.0470108358

agegrp60+ -0.084340339 0.0645143633

$HR

HR LB UB

agegrp30-44 0.9229817 0.8454953 1.007570

agegrp45-59 0.9829535 0.8964318 1.077826

agegrp60+ 0.9191184 0.8099472 1.043004

$ETR

ETR LB UB

agegrp30-44 1.028953 0.9973636 1.061542

agegrp45-59 1.006142 0.9736690 1.039697

agegrp60+ 1.030491 0.9851158 1.077956

> occ<-survreg(Surv(int_exp_test_plus1, status)~ Geslacht,

+ data= subset_compint, dist='weibull')

> summary(occ)

Call:

survreg(formula = Surv(int_exp_test_plus1, status) ~ Geslacht,

data = subset_compint, dist = "weibull")

Value Std. Error z p

(Intercept) 1.78352 0.00899 198.31 <2e-16

GeslachtMale -0.01216 0.01270 -0.96 0.34

Log(scale) -1.03026 0.01417 -72.70 <2e-16

Scale= 0.357

Weibull distribution

Loglik(model)= -6812.2 Loglik(intercept only)= -6812.6

Chisq= 0.92 on 1 degrees of freedom, p= 0.34

Number of Newton-Raphson Iterations: 7

n=3162 (9 observations deleted due to missingness)

> ConvertWeibull(occ, conf.level = 0.95)

$vars

Estimate SE

lambda 0.006757968 0.0005382991

gamma 2.801780528 0.0397046391

GeslachtMale 0.034071179 0.0355897098

$HR

HR LB UB

GeslachtMale 1.034658 0.9649458 1.109407

$ETR

ETR LB UB

GeslachtMale 0.9879131 0.9636178 1.012821

> occ<-survreg(Surv(int_exp_test_plus1, status)~ location,

+ data= subset_compint, dist='weibull')

> summary(occ)

Call:

survreg(formula = Surv(int_exp_test_plus1, status) ~ location,

data = subset_compint, dist = "weibull")

Value Std. Error z p

(Intercept) 1.79168 0.01253 143.05 <2e-16

locationIJsselland -0.03733 0.01725 -2.16 0.03

locationRotterdam-Rijnmond -0.00795 0.01558 -0.51 0.61

Log(scale) -1.03181 0.01415 -72.91 <2e-16

Scale= 0.356

Weibull distribution

Loglik(model)= -6826 Loglik(intercept only)= -6828.7

Chisq= 5.45 on 2 degrees of freedom, p= 0.065

Number of Newton-Raphson Iterations: 7

n= 3171

> ConvertWeibull(occ, conf.level = 0.95)

$vars

Estimate SE

lambda 0.006553606 0.0005418818

gamma 2.806153938 0.0397096858

locationIJsselland 0.104755102 0.0483713788

locationRotterdam-Rijnmond 0.022312957 0.0436955987

$HR

HR LB UB

locationIJsselland 1.110439 1.0099986 1.220867

locationRotterdam-Rijnmond 1.022564 0.9386348 1.113997

$ETR

ETR LB UB

locationIJsselland 0.9633577 0.9313301 0.9964867

locationRotterdam-Rijnmond 0.9920801 0.9622515 1.0228333

> occ<-survreg(Surv(int_exp_test_plus1, status)~ Gevaccineerd,

+ data= subset_compint, dist='weibull')

> summary(occ)

Call:

survreg(formula = Surv(int_exp_test_plus1, status) ~ Gevaccineerd,

data = subset_compint, dist = "weibull")

Value Std. Error z p

(Intercept) 1.77371 0.00693 255.82 <2e-16

Gevaccineerdja 0.03787 0.02089 1.81 0.07

Log(scale) -1.03200 0.01415 -72.94 <2e-16

Scale= 0.356

Weibull distribution

Loglik(model)= -6825 Loglik(intercept only)= -6826.7

Chisq= 3.38 on 1 degrees of freedom, p= 0.066

Number of Newton-Raphson Iterations: 7

n=3170 (1 observation deleted due to missingness)

> ConvertWeibull(occ, conf.level = 0.95)

$vars

Estimate SE

lambda 0.006886312 0.0005367685

gamma 2.806677953 0.0397092891

Gevaccineerdja -0.106291654 0.0586443890

$HR

HR LB UB

Gevaccineerdja 0.8991624 0.8015303 1.008687

$ETR

ETR LB UB

Gevaccineerdja 1.038597 0.9969306 1.082005

> occ<-survreg(Surv(int_exp_test_plus1, status)~ Eerdercorona,

+ data= subset_compint, dist='weibull')

> summary(occ)

Call:

survreg(formula = Surv(int_exp_test_plus1, status) ~ Eerdercorona,

data = subset_compint, dist = "weibull")

Value Std. Error z p

(Intercept) 1.77744 0.00691 257.39 <2e-16

Eerdercoronaja -0.00124 0.02189 -0.06 0.95

Log(scale) -1.03202 0.01417 -72.81 <2e-16

Scale= 0.356

Weibull distribution

Loglik(model)= -6807.8 Loglik(intercept only)= -6807.8

Chisq= 0 on 1 degrees of freedom, p= 0.95

Number of Newton-Raphson Iterations: 7

n=3162 (9 observations deleted due to missingness)

> ConvertWeibull(occ, conf.level = 0.95)

$vars

Estimate SE

lambda 0.006813815 0.0005327859

gamma 2.806731730 0.0397833034

Eerdercoronaja 0.003473939 0.0614261592

$HR

HR LB UB

Eerdercoronaja 1.00348 0.8896571 1.131865

$ETR

ETR LB UB

Eerdercoronaja 0.998763 0.9568277 1.042536

> #Multivariable Weibull regression model

> occ<-survreg(Surv(int_exp_test_plus1, status)~ W_app + W_GGD + W_pers + W_huisgenoot + W_self + W_unknown + Klachtenzz + Uitslag_PCR + agegrp + Geslacht + location + Gevaccineerd + Eerdercorona,

+ data= subset_compint, dist='weibull')

> summary(occ)

Call:

survreg(formula = Surv(int_exp_test_plus1, status) ~ W_app +

W_GGD + W_pers + W_huisgenoot + W_self + W_unknown + Klachtenzz +

Uitslag_PCR + agegrp + Geslacht + location + Gevaccineerd +

Eerdercorona, data = subset_compint, dist = "weibull")

Value Std. Error z p

(Intercept) 1.86187 0.03095 60.15 < 2e-16

W_appYes 0.08377 0.02742 3.05 0.0023

W_GGDYes 0.09644 0.02303 4.19 2.8e-05

W_persYes 0.00849 0.02134 0.40 0.6908

W_huisgenootYes -0.16298 0.01894 -8.61 < 2e-16

W_selfNo -0.04074 0.03403 -1.20 0.2313

W_unknownYes -0.00462 0.02742 -0.17 0.8662

KlachtenzzYes -0.01814 0.01454 -1.25 0.2122

Uitslag_PCRPositive -0.04671 0.02009 -2.33 0.0201

agegrp30-44 0.00618 0.01615 0.38 0.7019

agegrp45-59 0.00617 0.01690 0.36 0.7152

agegrp60+ -0.02258 0.02562 -0.88 0.3782

GeslachtMale -0.00962 0.01278 -0.75 0.4514

locationIJsselland -0.05650 0.01779 -3.18 0.0015

locationRotterdam-Rijnmond -0.00595 0.01571 -0.38 0.7050

Gevaccineerdja 0.03223 0.02350 1.37 0.1701

Eerdercoronaja -0.00622 0.02210 -0.28 0.7784

Log(scale) -1.03673 0.01424 -72.82 < 2e-16

Scale= 0.355

Weibull distribution

Loglik(model)= -6691.3 Loglik(intercept only)= -6768.4

Chisq= 154.07 on 16 degrees of freedom, p= 1.2e-24

Number of Newton-Raphson Iterations: 8

n=3142 (29 observations deleted due to missingness)

> ConvertWeibull(occ, conf.level = 0.95)

$vars

Estimate SE

lambda 0.005245369 0.0006095708

gamma 2.819967728 0.0401457548

W_appYes -0.236215286 0.0774870371

W_GGDYes -0.271955397 0.0650109204

W_persYes -0.023939111 0.0601856316

W_huisgenootYes 0.459593414 0.0530435866

W_selfNo 0.114880168 0.0960017872

W_unknownYes 0.013026344 0.0773295610

KlachtenzzYes 0.051159327 0.0409780310

Uitslag_PCRPositive 0.131731243 0.0566064083

agegrp30-44 -0.017431275 0.0455563433

agegrp45-59 -0.017386372 0.0476605660

agegrp60+ 0.063671913 0.0722618185

GeslachtMale 0.027134510 0.0360292589

locationIJsselland 0.159328350 0.0501060481

locationRotterdam-Rijnmond 0.016772739 0.0442982658

Gevaccineerdja -0.090898257 0.0662659669

Eerdercoronaja 0.017536338 0.0623112841

$HR

HR LB UB

W_appYes 0.7896107 0.6783533 0.9191155

W_GGDYes 0.7618882 0.6707395 0.8654235

W_persYes 0.9763452 0.8677073 1.0985845

W_huisgenootYes 1.5834301 1.4270793 1.7569106

W_selfNo 1.1217390 0.9293407 1.3539689

W_unknownYes 1.0131116 0.8706312 1.1789091

KlachtenzzYes 1.0524906 0.9712648 1.1405092

Uitslag_PCRPositive 1.1408017 1.0210022 1.2746578

agegrp30-44 0.9827198 0.8987772 1.0745022

agegrp45-59 0.9827639 0.8951183 1.0789913

agegrp60+ 1.0657427 0.9250027 1.2278964

GeslachtMale 1.0275060 0.9574502 1.1026877

locationIJsselland 1.1727229 1.0630289 1.2937363

locationRotterdam-Rijnmond 1.0169142 0.9323470 1.1091520

Gevaccineerdja 0.9131106 0.8018954 1.0397504

Eerdercoronaja 1.0176910 0.9006923 1.1498876

$ETR

ETR LB UB

W_appYes 1.0873736 1.0304749 1.1474140

W_GGDYes 1.1012426 1.0526355 1.1520942

W_persYes 1.0085253 0.9672038 1.0516121

W_huisgenootYes 0.8496097 0.8186507 0.8817394

W_selfNo 0.9600805 0.8981331 1.0263006

W_unknownYes 0.9953913 0.9433046 1.0503541

KlachtenzzYes 0.9820218 0.9544264 1.0104150

Uitslag_PCRPositive 0.9543606 0.9175097 0.9926915

agegrp30-44 1.0062005 0.9748506 1.0385586

agegrp45-59 1.0061845 0.9734051 1.0400678

agegrp60+ 0.9776740 0.9297844 1.0280303

GeslachtMale 0.9904239 0.9659275 1.0155414

locationIJsselland 0.9450664 0.9126738 0.9786087

locationRotterdam-Rijnmond 0.9940698 0.9639233 1.0251591

Gevaccineerdja 1.0327589 0.9862789 1.0814294

Eerdercoronaja 0.9938007 0.9516807 1.0377848

> #Asymptomatic Weibull regression model

> asymp<- subset_compint %>%

+ filter(subset_compint$Klachtenzz == "No")

> occ<-survreg(Surv(int_exp_test_plus1, status)~ W_app + W_GGD+ W_pers + W_huisgenoot + W_self + W_unknown + Uitslag_PCR + agegrp + Geslacht + location + Gevaccineerd + Eerdercorona,

+ data= asymp, dist='weibull')

> summary(occ)

Call:

survreg(formula = Surv(int_exp_test_plus1, status) ~ W_app +

W_GGD + W_pers + W_huisgenoot + W_self + W_unknown + Uitslag_PCR +

agegrp + Geslacht + location + Gevaccineerd + Eerdercorona,

data = asymp, dist = "weibull")

Value Std. Error z p

(Intercept) 1.89e+00 3.39e-02 55.81 <2e-16

W_appYes 3.87e-02 2.79e-02 1.39 0.1657

W_GGDYes 7.49e-02 2.42e-02 3.10 0.0020

W_persYes -8.21e-05 2.24e-02 0.00 0.9971

W_huisgenootYes -1.00e-01 2.05e-02 -4.89 1e-06

W_selfNo -5.57e-02 3.74e-02 -1.49 0.1365

W_unknownYes 2.61e-02 2.88e-02 0.91 0.3646

Uitslag_PCRPositive -8.18e-02 2.71e-02 -3.02 0.0025

agegrp30-44 -9.97e-04 1.77e-02 -0.06 0.9551

agegrp45-59 -6.57e-03 1.80e-02 -0.36 0.7158

agegrp60+ -4.60e-02 2.56e-02 -1.80 0.0717

GeslachtMale -1.20e-02 1.38e-02 -0.88 0.3813

locationIJsselland -3.74e-02 1.94e-02 -1.93 0.0535

locationRotterdam-Rijnmond -2.01e-02 1.68e-02 -1.20 0.2312

Gevaccineerdja 3.90e-02 2.41e-02 1.62 0.1053

Eerdercoronaja -2.30e-02 2.35e-02 -0.98 0.3282

Log(scale) -1.15e+00 1.69e-02 -68.02 <2e-16

Scale= 0.316

Weibull distribution

Loglik(model)= -4478.4 Loglik(intercept only)= -4512.4

Chisq= 68 on 15 degrees of freedom, p= 1e-08

Number of Newton-Raphson Iterations: 8

n=2168 (10 observations deleted due to missingness)

> ConvertWeibull(occ, conf.level = 0.95)

$vars

Estimate SE

lambda 0.0025118522 0.000377281

gamma 3.1648974266 0.053606924

W_appYes -0.1224204121 0.088364084

W_GGDYes -0.2369957543 0.076477759

W_persYes 0.0002599963 0.071018355

W_huisgenootYes 0.3175263509 0.064482301

W_selfNo 0.1762055086 0.118450079

W_unknownYes -0.0827312312 0.091282377

Uitslag_PCRPositive 0.2588142064 0.085686892

agegrp30-44 0.0031542528 0.055965102

agegrp45-59 0.0207865708 0.057095761

agegrp60+ 0.1457170685 0.080927096

GeslachtMale 0.0381299428 0.043536202

locationIJsselland 0.1184428184 0.061242202

locationRotterdam-Rijnmond 0.0637464612 0.053211911

Gevaccineerdja -0.1233878758 0.076205180

Eerdercoronaja 0.0727749606 0.074451144

$HR

HR LB UB

W_appYes 0.8847763 0.7440769 1.0520809

W_GGDYes 0.7889946 0.6791662 0.9165835

W_persYes 1.0002600 0.8702860 1.1496452

W_huisgenootYes 1.3737254 1.2106330 1.5587892

W_selfNo 1.1926831 0.9455842 1.5043537

W_unknownYes 0.9205985 0.7697870 1.1009561

Uitslag_PCRPositive 1.2953931 1.0951277 1.5322809

agegrp30-44 1.0031592 0.8989433 1.1194571

agegrp45-59 1.0210041 0.9129090 1.1418985

agegrp60+ 1.1568688 0.9871857 1.3557181

GeslachtMale 1.0388662 0.9538971 1.1314040

locationIJsselland 1.1257425 0.9984116 1.2693124

locationRotterdam-Rijnmond 1.0658221 0.9602641 1.1829838

Gevaccineerdja 0.8839207 0.7612852 1.0263117

Eerdercoronaja 1.0754885 0.9294646 1.2444536

$ETR

ETR LB UB

W_appYes 1.0394385 0.9841178 1.0978690

W_GGDYes 1.0777576 1.0278682 1.1300685

W_persYes 0.9999179 0.9568941 1.0448760

W_huisgenootYes 0.9045411 0.8688842 0.9416613

W_selfNo 0.9458465 0.8790139 1.0177606

W_unknownYes 1.0264849 0.9700908 1.0861573

Uitslag_PCRPositive 0.9214779 0.8738747 0.9716742

agegrp30-44 0.9990039 0.9649716 1.0342363

agegrp45-59 0.9934537 0.9589367 1.0292131

agegrp60+ 0.9550022 0.9083354 1.0040666

GeslachtMale 0.9880245 0.9617319 1.0150359

locationIJsselland 0.9632677 0.9273637 1.0005618

locationRotterdam-Rijnmond 0.9800598 0.9482697 1.0129156

Gevaccineerdja 1.0397563 0.9918426 1.0899846

Eerdercoronaja 0.9772679 0.9332473 1.0233650

> #Linear model with tobit

> occ <- vglm(int_exp_test ~ W_app,

+ family= tobit(Lower = 0, Upper = 14), data = subset_compint)

> summary(occ)

Call:

vglm(formula = int_exp_test ~ W_app, family = tobit(Lower = 0,

Upper = 14), data = subset_compint)

Coefficients:

Estimate Std. Error z value Pr(>|z|)

(Intercept):1 4.21840 0.04006 105.310 <2e-16 ***

(Intercept):2 0.77855 0.01282 60.709 <2e-16 ***

W_appYes 0.38365 0.15783 2.431 0.0151 *

---

Signif. codes: 0 ‘***’ 0.001 ‘**’ 0.01 ‘*’ 0.05 ‘.’ 0.1 ‘ ’ 1

Names of linear predictors: mu, loglink(sd)

Log-likelihood: -6784.252 on 6339 degrees of freedom

Number of Fisher scoring iterations: 5

No Hauck-Donner effect found in any of the estimates

> b <- coef(occ)

> se <- sqrt(diag(vcov(occ)))

> cbind(LL = b - qnorm(0.975) * se, UL = b + qnorm(0.975) * se)

LL UL

(Intercept):1 4.1398877 4.2969077

(Intercept):2 0.7534104 0.8036800

W_appYes 0.0743187 0.6929844

> occ <- vglm(int_exp_test ~ W_GGD,

+ family= tobit(Lower = 0, Upper = 14), data = subset_compint)

> summary(occ)

Call:

vglm(formula = int_exp_test ~ W_GGD, family = tobit(Lower = 0,

Upper = 14), data = subset_compint)

Coefficients:

Estimate Std. Error z value Pr(>|z|)

(Intercept):1 4.22715 0.04067 103.94 <2e-16 ***

(Intercept):2 0.77918 0.01282 60.76 <2e-16 ***

W_GGDYes 0.17506 0.13466 1.30 0.194

---

Signif. codes: 0 ‘***’ 0.001 ‘**’ 0.01 ‘*’ 0.05 ‘.’ 0.1 ‘ ’ 1

Names of linear predictors: mu, loglink(sd)

Log-likelihood: -6786.353 on 6339 degrees of freedom

Number of Fisher scoring iterations: 5

No Hauck-Donner effect found in any of the estimates

> b <- coef(occ)

> se <- sqrt(diag(vcov(occ)))

> cbind(LL = b - qnorm(0.975) * se, UL = b + qnorm(0.975) * se)

LL UL

(Intercept):1 4.1474368 4.3068542

(Intercept):2 0.7540450 0.8043149

W_GGDYes -0.0888783 0.4389986

> occ <- vglm(int_exp_test ~ W_pers,

+ family= tobit(Lower = 0, Upper = 14), data = subset_compint)

> summary(occ)

Call:

vglm(formula = int_exp_test ~ W_pers, family = tobit(Lower = 0,

Upper = 14), data = subset_compint)

Coefficients:

Estimate Std. Error z value Pr(>|z|)

(Intercept):1 3.92384 0.06244 62.84 < 2e-16 ***

(Intercept):2 0.77379 0.01283 60.33 < 2e-16 ***

W_persYes 0.51519 0.07939 6.49 8.61e-11 ***

---

Signif. codes: 0 ‘***’ 0.001 ‘**’ 0.01 ‘*’ 0.05 ‘.’ 0.1 ‘ ’ 1

Names of linear predictors: mu, loglink(sd)

Log-likelihood: -6766.386 on 6339 degrees of freedom

Number of Fisher scoring iterations: 5

No Hauck-Donner effect found in any of the estimates

> b <- coef(occ)

> se <- sqrt(diag(vcov(occ)))

> cbind(LL = b - qnorm(0.975) * se, UL = b + qnorm(0.975) * se)

LL UL

(Intercept):1 3.8014560 4.0462263

(Intercept):2 0.7486486 0.7989222

W_persYes 0.3595900 0.6707828

> occ <- vglm(int_exp_test ~ W_huisgenoot,

+ family= tobit(Lower = 0, Upper = 14), data = subset_compint)

> summary(occ)

Call:

vglm(formula = int_exp_test ~ W_huisgenoot, family = tobit(Lower = 0,

Upper = 14), data = subset_compint)

Coefficients:

Estimate Std. Error z value Pr(>|z|)

(Intercept):1 4.68479 0.04212 111.23 <2e-16 ***

(Intercept):2 0.71853 0.01284 55.97 <2e-16 ***

W_huisgenootYes -1.77730 0.08443 -21.05 <2e-16 ***

---

Signif. codes: 0 ‘***’ 0.001 ‘**’ 0.01 ‘*’ 0.05 ‘.’ 0.1 ‘ ’ 1

Names of linear predictors: mu, loglink(sd)

Log-likelihood: -6579.972 on 6339 degrees of freedom

Number of Fisher scoring iterations: 6

No Hauck-Donner effect found in any of the estimates

> b <- coef(occ)

> se <- sqrt(diag(vcov(occ)))

> cbind(LL = b - qnorm(0.975) * se, UL = b + qnorm(0.975) * se)

LL UL

(Intercept):1 4.6022446 4.7673442

(Intercept):2 0.6933679 0.7436895

W_huisgenootYes -1.9427720 -1.6118259

> occ <- vglm(int_exp_test ~ W_self,

+ family= tobit(Lower = 0, Upper = 14), data = subset_compint)

> summary(occ)

Call:

vglm(formula = int_exp_test ~ W_self, family = tobit(Lower = 0,

Upper = 14), data = subset_compint)

Coefficients:

Estimate Std. Error z value Pr(>|z|)

(Intercept):1 4.45292 0.16404 27.146 <2e-16 ***

(Intercept):2 0.77922 0.01282 60.762 <2e-16 ***

W_selfNo -0.22228 0.16882 -1.317 0.188

---

Signif. codes: 0 ‘***’ 0.001 ‘**’ 0.01 ‘*’ 0.05 ‘.’ 0.1 ‘ ’ 1

Names of linear predictors: mu, loglink(sd)

Log-likelihood: -6786.329 on 6339 degrees of freedom

Number of Fisher scoring iterations: 5

No Hauck-Donner effect found in any of the estimates

> b <- coef(occ)

> se <- sqrt(diag(vcov(occ)))

> cbind(LL = b - qnorm(0.975) * se, UL = b + qnorm(0.975) * se)

LL UL

(Intercept):1 4.1314177 4.7744242

(Intercept):2 0.7540872 0.8043573

W_selfNo -0.5531519 0.1086017

> occ <- vglm(int_exp_test ~ W_unknown,

+ family= tobit(Lower = 0, Upper = 14), data = subset_compint)

> summary(occ)

Call:

vglm(formula = int_exp_test ~ W_unknown, family = tobit(Lower = 0,

Upper = 14), data = subset_compint)

Coefficients:

Estimate Std. Error z value Pr(>|z|)

(Intercept):1 4.20991 0.04231 99.496 <2e-16 ***

(Intercept):2 0.77888 0.01282 60.735 <2e-16 ***

W_unknownYes 0.20615 0.10547 1.955 0.0506 .

---

Signif. codes: 0 ‘***’ 0.001 ‘**’ 0.01 ‘*’ 0.05 ‘.’ 0.1 ‘ ’ 1

Names of linear predictors: mu, loglink(sd)

Log-likelihood: -6785.291 on 6339 degrees of freedom

Number of Fisher scoring iterations: 5

No Hauck-Donner effect found in any of the estimates

> b <- coef(occ)

> se <- sqrt(diag(vcov(occ)))

> cbind(LL = b - qnorm(0.975) * se, UL = b + qnorm(0.975) * se)

LL UL

(Intercept):1 4.1269749403 4.2928356

(Intercept):2 0.7537454014 0.8040153

W_unknownYes -0.0005657843 0.4128566

> occ <- vglm(int_exp_test ~ Klachtenzz,

+ family= tobit(Lower = 0, Upper = 14), data = subset_compint)

> summary(occ)

Call:

vglm(formula = int_exp_test ~ Klachtenzz, family = tobit(Lower = 0,

Upper = 14), data = subset_compint)

Coefficients:

Estimate Std. Error z value Pr(>|z|)

(Intercept):1 4.40212 0.04652 94.625 < 2e-16 ***

(Intercept):2 0.77400 0.01285 60.232 < 2e-16 ***

KlachtenzzYes -0.50843 0.08358 -6.084 1.18e-09 ***

---

Signif. codes: 0 ‘***’ 0.001 ‘**’ 0.01 ‘*’ 0.05 ‘.’ 0.1 ‘ ’ 1

Names of linear predictors: mu, loglink(sd)

Log-likelihood: -6741.271 on 6313 degrees of freedom

Number of Fisher scoring iterations: 5

No Hauck-Donner effect found in any of the estimates

> b <- coef(occ)

> se <- sqrt(diag(vcov(occ)))

> cbind(LL = b - qnorm(0.975) * se, UL = b + qnorm(0.975) * se)

LL UL

(Intercept):1 4.3109418 4.4933044

(Intercept):2 0.7488122 0.7991848

KlachtenzzYes -0.6722360 -0.3446277

> occ <- vglm(int_exp_test ~ Uitslag_PCR,

+ family= tobit(Lower = 0, Upper = 14), data = subset_compint)

> summary(occ)

Call:

vglm(formula = int_exp_test ~ Uitslag_PCR, family = tobit(Lower = 0,

Upper = 14), data = subset_compint)

Coefficients:

Estimate Std. Error z value Pr(>|z|)

(Intercept):1 4.37330 0.04107 106.473 <2e-16 ***

(Intercept):2 0.76822 0.01283 59.893 <2e-16 ***

Uitslag_PCRPositive -1.01693 0.11468 -8.867 <2e-16 ***

---

Signif. codes: 0 ‘***’ 0.001 ‘**’ 0.01 ‘*’ 0.05 ‘.’ 0.1 ‘ ’ 1

Names of linear predictors: mu, loglink(sd)

Log-likelihood: -6748.798 on 6339 degrees of freedom

Number of Fisher scoring iterations: 5

No Hauck-Donner effect found in any of the estimates

> b <- coef(occ)

> se <- sqrt(diag(vcov(occ)))

> cbind(LL = b - qnorm(0.975) * se, UL = b + qnorm(0.975) * se)

LL UL

(Intercept):1 4.2927922 4.4538007

(Intercept):2 0.7430765 0.7933554

Uitslag_PCRPositive -1.2417047 -0.7921620

> occ <- vglm(int_exp_test ~ agegrp,

+ family= tobit(Lower = 0, Upper = 14), data = subset_compint)

> summary(occ)

Call:

vglm(formula = int_exp_test ~ agegrp, family = tobit(Lower = 0,

Upper = 14), data = subset_compint)

Coefficients:

Estimate Std. Error z value Pr(>|z|)

(Intercept):1 4.29630 0.05732 74.947 <2e-16 ***

(Intercept):2 0.77916 0.01284 60.694 <2e-16 ***

agegrp30-44 -0.13207 0.09743 -1.356 0.1752

agegrp45-59 -0.19172 0.10258 -1.869 0.0616 .

agegrp60+ 0.20103 0.14078 1.428 0.1533

---

Signif. codes: 0 ‘***’ 0.001 ‘**’ 0.01 ‘*’ 0.05 ‘.’ 0.1 ‘ ’ 1

Names of linear predictors: mu, loglink(sd)

Log-likelihood: -6772.037 on 6325 degrees of freedom

Number of Fisher scoring iterations: 5

No Hauck-Donner effect found in any of the estimates

> b <- coef(occ)

> se <- sqrt(diag(vcov(occ)))

> cbind(LL = b - qnorm(0.975) * se, UL = b + qnorm(0.975) * se)

LL UL

(Intercept):1 4.18394799 4.408656556

(Intercept):2 0.75400312 0.804325631

agegrp30-44 -0.32303899 0.058891537

agegrp45-59 -0.39276620 0.009324483

agegrp60+ -0.07489029 0.476953853

> occ <- vglm(int_exp_test ~ Geslacht,

+ family= tobit(Lower = 0, Upper = 14), data = subset_compint)

> summary(occ)

Call:

vglm(formula = int_exp_test ~ Geslacht, family = tobit(Lower = 0,

Upper = 14), data = subset_compint)

Coefficients:

Estimate Std. Error z value Pr(>|z|)

(Intercept):1 4.30342 0.05401 79.674 <2e-16 ***

(Intercept):2 0.78031 0.01284 60.751 <2e-16 ***

GeslachtMale -0.12721 0.07778 -1.635 0.102

---

Signif. codes: 0 ‘***’ 0.001 ‘**’ 0.01 ‘*’ 0.05 ‘.’ 0.1 ‘ ’ 1

Names of linear predictors: mu, loglink(sd)

Log-likelihood: -6769.614 on 6321 degrees of freedom

Number of Fisher scoring iterations: 5

No Hauck-Donner effect found in any of the estimates

> b <- coef(occ)

> se <- sqrt(diag(vcov(occ)))

> cbind(LL = b - qnorm(0.975) * se, UL = b + qnorm(0.975) * se)

LL UL

(Intercept):1 4.1975575 4.40928337

(Intercept):2 0.7551321 0.80548061

GeslachtMale -0.2796618 0.02524336

> occ <- vglm(int_exp_test ~ location,

+ family= tobit(Lower = 0, Upper = 14), data = subset_compint)

> summary(occ)

Call:

vglm(formula = int_exp_test ~ location, family = tobit(Lower = 0,

Upper = 14), data = subset_compint)

Coefficients:

Estimate Std. Error z value Pr(>|z|)

(Intercept):1 4.47566 0.07606 58.846 < 2e-16 ***

(Intercept):2 0.77663 0.01282 60.562 < 2e-16 ***

locationIJsselland -0.43348 0.10532 -4.116 3.86e-05 ***

locationRotterdam-Rijnmond -0.23978 0.09506 -2.522 0.0117 *

---

Signif. codes: 0 ‘***’ 0.001 ‘**’ 0.01 ‘*’ 0.05 ‘.’ 0.1 ‘ ’ 1

Names of linear predictors: mu, loglink(sd)

Log-likelihood: -6778.779 on 6338 degrees of freedom

Number of Fisher scoring iterations: 5

No Hauck-Donner effect found in any of the estimates

> b <- coef(occ)

> se <- sqrt(diag(vcov(occ)))

> cbind(LL = b - qnorm(0.975) * se, UL = b + qnorm(0.975) * se)

LL UL

(Intercept):1 4.3265908 4.62472732

(Intercept):2 0.7514967 0.80176488

locationIJsselland -0.6399116 -0.22705673

locationRotterdam-Rijnmond -0.4260917 -0.05346089

> occ <- vglm(int_exp_test ~ Gevaccineerd,

+ family= tobit(Lower = 0, Upper = 14), data = subset_compint)

> summary(occ)

Call:

vglm(formula = int_exp_test ~ Gevaccineerd, family = tobit(Lower = 0,

Upper = 14), data = subset_compint)

Coefficients:

Estimate Std. Error z value Pr(>|z|)

(Intercept):1 4.21765 0.04092 103.080 <2e-16 ***

(Intercept):2 0.77895 0.01283 60.730 <2e-16 ***

Gevaccineerdja 0.24335 0.12793 1.902 0.0571 .

---

Signif. codes: 0 ‘***’ 0.001 ‘**’ 0.01 ‘*’ 0.05 ‘.’ 0.1 ‘ ’ 1

Names of linear predictors: mu, loglink(sd)

Log-likelihood: -6783.369 on 6337 degrees of freedom

Number of Fisher scoring iterations: 5

No Hauck-Donner effect found in any of the estimates

> b <- coef(occ)

> se <- sqrt(diag(vcov(occ)))

> cbind(LL = b - qnorm(0.975) * se, UL = b + qnorm(0.975) * se)

LL UL

(Intercept):1 4.137452182 4.2978413

(Intercept):2 0.753814311 0.8040930

Gevaccineerdja -0.007385024 0.4940761

> occ <- vglm(int_exp_test ~ Eerdercorona,

+ family= tobit(Lower = 0, Upper = 14), data = subset_compint)

> summary(occ)

Call:

vglm(formula = int_exp_test ~ Eerdercorona, family = tobit(Lower = 0,

Upper = 14), data = subset_compint)

Coefficients:

Estimate Std. Error z value Pr(>|z|)

(Intercept):1 4.23194 0.04075 103.854 <2e-16 ***

(Intercept):2 0.77908 0.01284 60.663 <2e-16 ***

Eerdercoronaja 0.09343 0.13406 0.697 0.486

---

Signif. codes: 0 ‘***’ 0.001 ‘**’ 0.01 ‘*’ 0.05 ‘.’ 0.1 ‘ ’ 1

Names of linear predictors: mu, loglink(sd)

Log-likelihood: -6766.016 on 6321 degrees of freedom

Number of Fisher scoring iterations: 5

No Hauck-Donner effect found in any of the estimates

> b <- coef(occ)

> se <- sqrt(diag(vcov(occ)))

> cbind(LL = b - qnorm(0.975) * se, UL = b + qnorm(0.975) * se)

LL UL

(Intercept):1 4.1520732 4.3118054

(Intercept):2 0.7539056 0.8042485

Eerdercoronaja -0.1693251 0.3561908

> occ <- vglm(int_exp_test ~ W_app + W_GGD + W_pers + W_huisgenoot + W_self + W_unknown + Klachtenzz + Uitslag_PCR + agegrp + Geslacht + location + Gevaccineerd + Eerdercorona,

+ family= tobit(Lower = 0, Upper = 14), data = subset_compint)

> summary(occ)

Call:

vglm(formula = int_exp_test ~ W_app + W_GGD + W_pers + W_huisgenoot +

W_self + W_unknown + Klachtenzz + Uitslag_PCR + agegrp +

Geslacht + location + Gevaccineerd + Eerdercorona, family = tobit(Lower = 0,

Upper = 14), data = subset_compint)

Coefficients:

Estimate Std. Error z value Pr(>|z|)

(Intercept):1 5.21012 0.17799 29.273 < 2e-16 ***

(Intercept):2 0.69824 0.01290 54.140 < 2e-16 ***

W_appYes 0.31207 0.15663 1.992 0.04633 *

W_GGDYes 0.62692 0.13001 4.822 1.42e-06 ***

W_persYes 0.21843 0.11736 1.861 0.06272 .

W_huisgenootYes -1.77506 0.10344 -17.160 < 2e-16 ***

W_selfNo -0.16575 0.18406 -0.900 0.36786

W_unknownYes -0.09573 0.15243 -0.628 0.52999

KlachtenzzYes -0.32311 0.08195 -3.943 8.05e-05 ***

Uitslag_PCRPositive -0.50626 0.11399 -4.441 8.94e-06 ***

agegrp30-44 -0.24350 0.09075 -2.683 0.00729 **

agegrp45-59 -0.10967 0.09686 -1.132 0.25751

agegrp60+ -0.18854 0.14659 -1.286 0.19838

GeslachtMale -0.12506 0.07258 -1.723 0.08488 .

locationIJsselland -0.55560 0.10093 -5.505 3.70e-08 ***

locationRotterdam-Rijnmond -0.23069 0.08944 -2.579 0.00990 **

Gevaccineerdja 0.18299 0.13451 1.360 0.17369

Eerdercoronaja 0.01060 0.12541 0.085 0.93263

---

Signif. codes: 0 ‘***’ 0.001 ‘**’ 0.01 ‘*’ 0.05 ‘.’ 0.1 ‘ ’ 1

Names of linear predictors: mu, loglink(sd)

Log-likelihood: -6452.286 on 6266 degrees of freedom

Number of Fisher scoring iterations: 6

No Hauck-Donner effect found in any of the estimates

> b <- coef(occ)

> se <- sqrt(diag(vcov(occ)))

> cbind(LL = b - qnorm(0.975) * se, UL = b + qnorm(0.975) * se)

LL UL

(Intercept):1 4.861278664 5.55897015

(Intercept):2 0.672964458 0.72351972

W_appYes 0.005082827 0.61906355

W_GGDYes 0.372104868 0.88172752

W_persYes -0.011595799 0.44845040

W_huisgenootYes -1.977796997 -1.57231960

W_selfNo -0.526507626 0.19501241

W_unknownYes -0.394485837 0.20302838

KlachtenzzYes -0.483718162 -0.16249590

Uitslag_PCRPositive -0.729683961 -0.28284514

agegrp30-44 -0.421359039 -0.06563387

agegrp45-59 -0.299508055 0.08016544

agegrp60+ -0.475851915 0.09876975

GeslachtMale -0.267322429 0.01719653

locationIJsselland -0.753418494 -0.35778099

locationRotterdam-Rijnmond -0.406000007 -0.05538817

Gevaccineerdja -0.080638743 0.44661280

Eerdercoronaja -0.235192702 0.25639407
